# Temporally Controlled Supramolecular Catalysts with pH-Dependent Activity

**DOI:** 10.1021/acsomega.5c11122

**Published:** 2026-01-22

**Authors:** Giulio Pucciarelli, Francesco Ranieri, Alessandro Casnati, Stefano Di Stefano, Stefano Volpi, Riccardo Salvio

**Affiliations:** † Dipartimento di Scienze e Tecnologie Chimiche, Università“Tor Vergata”, Via Della Ricerca Scientifica, 1, 00133 Roma, Italy; ‡ Dipartimento di Chimica, Università di Roma La Sapienza, P.le A. Moro 5, 00185 Roma, Italy; § Dipartimento di Scienze Chimiche, Della Vita e Della Sostenibilità Ambientale, 9370 Università Degli Studi di Parma, Parco Area Delle Scienze, 17/A, 43124 Parma, Italy; ∥ ISB–CNR Sezione Meccanismi di Reazione, Università La Sapienza, P.le A. Moro 5, 00185 Roma, Italy

## Abstract

Controlling the activity of synthetic catalysts over time remains a key challenge for designing adaptive chemical systems. Supramolecular phosphodiesterase mimics can be particularly sensitive to pH, with some of them presenting active species that operate only under basic conditions. In this work, we have focused on a dissipative strategy that exploits activated carboxylic acids (ACAs) to temporally modulate pH and, consequently, the activity states of these catalysts. ACAs undergo combined acid–base and decarboxylation processes, enabling transient acidification followed by a spontaneous return to higher pH. We first analyze the acid–base behavior of a selected ACA through potentiometric studies to identify the parameters governing the lifetime of the dissipative state in semiaqueous media. Guided by these insights, we investigate the time-dependent catalytic performance of metal complexes based on a cyclic polyamine and a bifunctional calix[4]­arene bearing both a cyclic polyamine and a guanidinium group. This approach provides a programmable way to regulate phosphodiester cleavage catalysis, laying the foundations for future adaptive and temporally controlled chemical systems.

## Graphical abstract

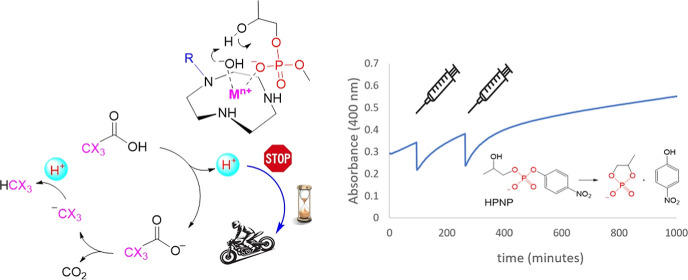

## Introduction

Living systems demonstrate the capacity of nature to realize complex and advanced functions such as stimuli responsiveness and adaptability. The execution of these functions is often sustained by the absorption of light and the consumption (dissipation) of chemical species.^1–14^ Inspired by these natural paradigms, numerous research groups have recently dedicated efforts to the design and investigation of artificial chemical systems engineered to persist in a dissipative state as long as the stimulus is present.^1–14^ In particular, dissipative systems have been used to regulate complexation processes,^15–20^ determine the state of molecular switches,^21–25^ or control signal activation and deactivation.^26,27^ In this context, the ability to design catalysts that can be reversibly switched between their inactive and active states represents a particularly intriguing prospect, as this aspect remains poorly explored in the field of dissipative systems.^28–35^

Within this framework, artificial phosphodiesterases^36–44^ represent an excellent platform for the development of stimuli-responsive catalytic systems, essentially for two reasons: (i) they have been thoroughly investigated by several groups, and a few highly efficient polyfunctional catalysts are available with well-characterized behaviors;^37,39,40,42–44^ (ii) in metal-based (ribo)­nucleases, the catalytic mechanism relies on the Lewis acid properties of the metal center, which coordinates a water molecule.^37,45,46^ This coordinated water features a p*K*_a_ in the range useful for the catalytic experiments, i.e., pH 7.0–9.0. Since the deprotonated form has been identified as the active species of these catalysts,^37^ pH variation exerts a significant influence on their catalytic performance.

Therefore, a possible strategy to design a stimuli-responsive system based on pH variation consists of the use of activated carboxylic acids (ACAs). The addition of a proper amount of these compounds in solution can reversibly change the pH with a sudden drop followed by a slow recovery of the initial condition.^47–49^ The latter step proceeds through a decarboxylation reaction, generating a strong base that retrieves the proton originally donated by the acid (Scheme 1). Recent literature reports indicate that these compounds can be successfully employed to achieve solutions with progressively increasing pH values, and the rate of pH variation can be modulated by altering the experimental conditions.^47,50–54^

**Scheme 1:**
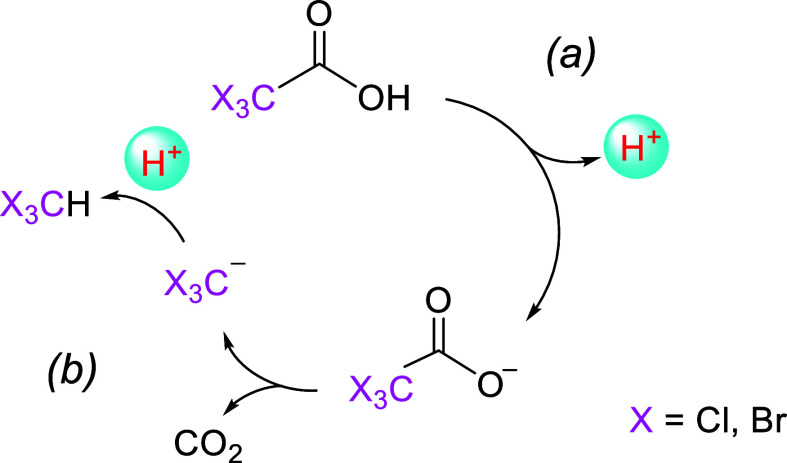
Mechanism of pH Variation in the Presence of TCA or TBA^a^

a The acid initially donates a proton in a diffusion-controlled step (a). Next, a slower reaction step leads to the formation of carbonic anhydride and a strong base, which recaptures the proton, causing a pH increase and the production of chloroform or bromoform as a waste (b).

In this paper, we show that trichloroacetic acid (TCA), a typical ACA, can be conveniently employed to temporally control the activation and deactivation of supramolecular catalysts with phosphodiesterase activity, i.e., the metal complex of a cyclic polyamine and a bifunctional calix[4]­arene.

## Results and Discussion

Study of pH variation: Understanding the acid–base behavior of the ACA, as well as the factors influencing its decarboxylation rate, is an essential prerequisite for a rigorous investigation of the catalytic properties. An 80:20 *v*/*v* mixture of dimethyl sulfoxide (DMSO) and water, hereafter referred to as 80% DMSO, was employed as the reaction medium. This semiaqueous solvent mixture is well suited for potentiometric experiments^55,56^ and has proven effective in investigations on phosphodiester bond cleavage because it improves the substrate–catalyst binding and slows down the spontaneous background hydrolysis.^37,42,57^ In this medium, the water autoprotolysis p*K*_w_ rises to 18.4, thereby shifting the neutrality value to 9.2.^58^ For the pH measurements, a glass pH electrode was employed with a proper calibration procedure as described in the Experimental Section (see also Section S4 in Supporting Information). This calibration procedure is based on the use of buffer solutions whose p*K*_a_ values had been previously determined in the literature under the exact same conditions used in the present work.^55^ This ensures good data linearity and quasi-Nernstian behavior.

When working with aqueous mixtures, tribromo- and trichloro, as well as nitroacetic acid,^47^ are the most suitable ACAs due to their better solubility. Preliminary experiments revealed that tribromoacetic acid (TBA) exhibits exceedingly high reactivity, with a half-life of only a few seconds, whereas the reactivity of nitroacetic acid is even more pronounced under the tested conditions (*vide infra*). In addition, dichloroacetic acid does not decarboxylate under the conditions adopted in the present investigation. In fact, we used 10 mM dichloroacetic acid together with 5 mM NMe_4_OH in this solvent mixture to calibrate our pH electrode (Section S4), and a stable reading was rapidly achieved with no evidence of decarboxylation. Derivatives of the 2-cyano-2-phenylpropanoic acid are known to undergo decarboxylation; however, they are almost insoluble in aqueous or semiaqueous media. For this reason, they were excluded from the initial ACA screening. Considering the response time of the pH meter, which requires several seconds, the half-life of the ACA should fall within the time scale of several minutes to hours.

Accordingly, the experiments were performed in the presence of TCA, which shows a decarboxylation process occurring over time scales from minutes to a few hours (Scheme 1).

A series of experiments were performed in the presence of KH_2_PO_4_ (1–10 mM) buffered at pH between 8.0 and 9.0, to which TCA was added at 5–50 mM final concentrations (see Section S1 in Supporting Information). Analogous experiments were performed in the presence of diisopropylethylamine and perchloric acid as buffers in similar concentrations. Given the incomplete recovery of the initial pH observed in this series of experiments,^59^ potassium carbonate was subsequently employed as a buffering salt. In this case, nearly full restoration of the initial pH value can be achieved, *vide infra*. In addition, potassium carbonate buffer ensures stable pH measurement in either acidic or basic conditions as well as around neutrality due to its equilibrium with the carbonic anhydride produced by the decarboxylation process or possibly absorbed from the atmosphere. Therefore, the carbonate buffer proved to be more robust and better suited to our purposes.

Several parameters can be changed such as the buffer concentration, initial pH value of the solution, and amount and number of TCA additions. In Figure 1, a plot of the variation of the pH versus time is reported for a 10 mM solution of potassium carbonate buffered at pH = 12 through the addition of perchloric acid. Addition of TCA at a final concentration of 10 mM results in an instantaneous drop to pH 2.3. The solution remains under pH 4, exhibiting a slow increase for approximately 30 min, a phase during which the system can be classified as being in a dissipative state. After this period, however, it starts to rise much more rapidly, eventually reaching a value of about 8. A second addition of the same amount of TCA causes a similar drop. However, the duration of the dissipative state is around 50 min. After the second addition, the pH goes back to a pH value very similar to that reached after the first addition. The third acid addition results in the same response as that of the second, both in behavior and in the duration of the dissipative state.

**Figure 1:**
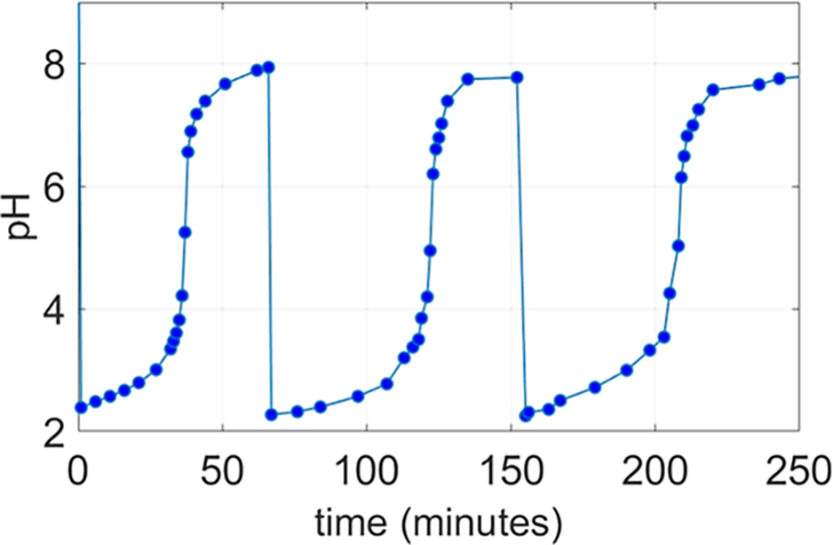
pH versus time profile of a 10 mM potassium carbonate solution in 80% DMSO upon three equimolar consecutive additions of 10 mM TCA each, *T* = 25 °C.

Across all experiments (see Section S1 in the Supporting Information), the initial addition of TCA consistently leads to a shorter-lived dissipative state than those observed in the subsequent additions, even though the amount of TCA added remains constant. This is probably due to the formation of CO_2_, which initially alters the buffer composition. Conversely, in the following additions, an equilibrium is reached due to the saturation of the reaction medium, and as a result of that, these dissipative states have similar durations.

The decarboxylation rate—and consequently the duration of the dissipative state—may be influenced by the concentration of the species and the nature of the counterions associated with the trichloroacetate ion.^47^ For this reason, we conducted a series of experiments in which the concentrations of the components in solution were systematically varied (see Section S1 in the Supporting Information). In these experiments, the initial pH of the solution was not preadjusted with perchloric acid but was instead varied only by TCA additions. Consequently, a portion of TCA in the first addition was consumed, leading to a reduced duration of the first dissipative state. The results proved to be highly regular and more reproducible in comparison with those observed with a preadjustment of the pH with perchloric acid (see Figure S1.17 in Supporting Information), likely due to the inherent instability of measurements of the preadjusted pH under strongly basic conditions in this solvent mixture.

A plot for the duration of the dissipative state as a function of the TCA concentration is reported in Figure 2a. The plot indicates perfect linearity with an evident increase of the duration on increasing acid concentration. This evidence clearly points out the possibility of achieving temporal control of the pH and therefore of the catalytic activity, *vide infra*. In another series of experiments, the K_2_CO_3_ concentration varied from 5.0 to 15.0 mM (see Figure 2b) while maintaining constant TCA additions. The plot clearly highlights that higher concentrations of carbonate buffer shorten the dissipative state. This behavior, which may be consistent with a general-base mechanism, could arise from a faster decarboxylation when the proton is removed from the vicinity of the trichloroacetate ion by the basic component of the buffer, possibly leading to the formation of a solvent-separated ion pair. Therefore, the duration of the dissipative state is governed by the excess acid relative to the concentration of the basic buffer component. The system remains in the dissipative state as long as there is an excess acid. Once the excess acid runs out, decarboxylation and chloroform formation as waste materials accelerate significantly, causing a sharp rise in the pH value.

**Figure 2:**
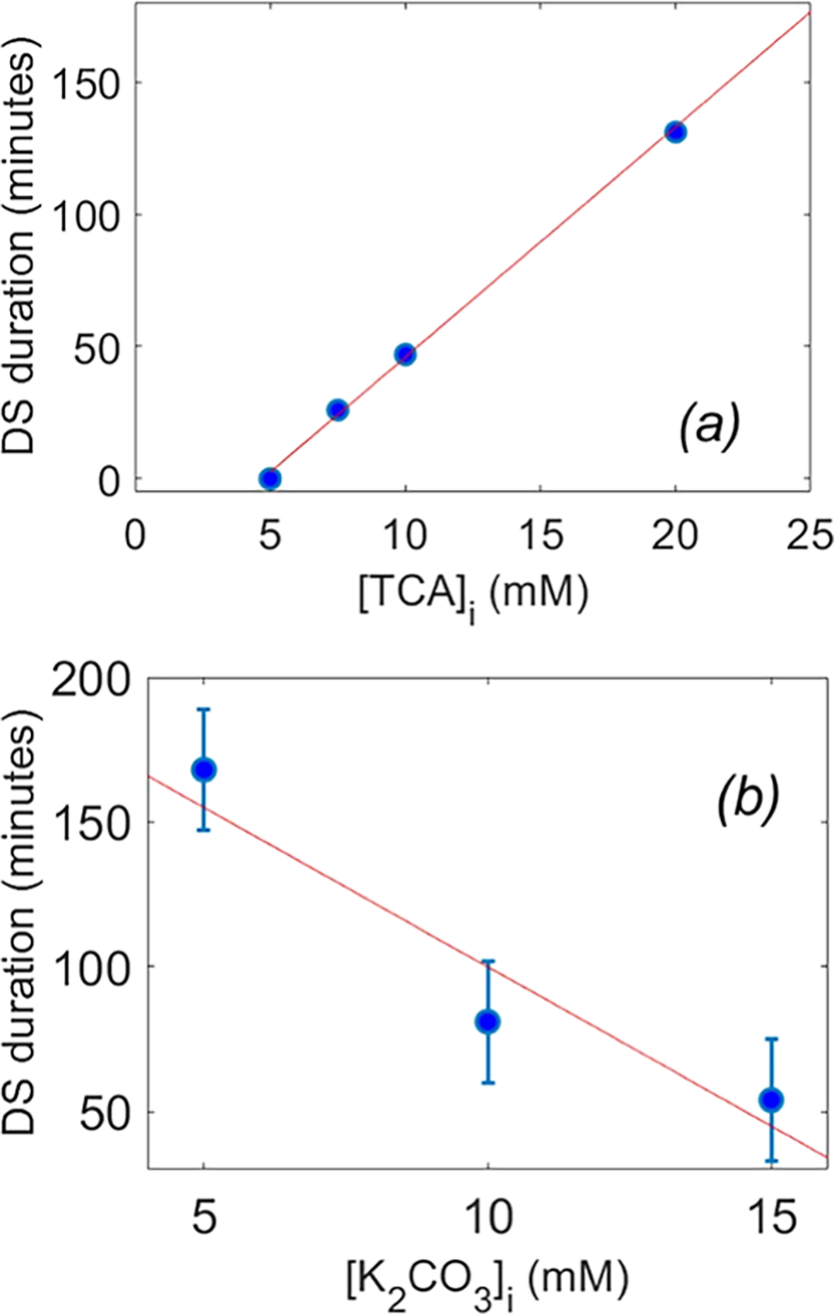
Plot of the duration of the dissipative state after the second addition versus the TCA initial concentration, 10 mM carbonate buffer (a), and initial potassium carbonate concentration, 10 mM additions of TCA (b); 80% DMSO, *T* = 25 °C.

In addition to potentiometric measurements, the decarboxylation reaction was also monitored spectrophotometrically by introducing 30 μM *p*-nitrophenol. This compound effectively functions as a pH indicator, allowing the temporal evolution of pH to be followed by recording the absorbance at 400 nm (Section S2 in the Supporting Information). The profiles and durations of the observed dissipative states were in close agreement with those obtained from potentiometric measurements. Moreover, this approach enabled the determination of the molar extinction coefficients for both the acid and the conjugate base forms of *p*-nitrophenol in this solvent mixture.

It is noteworthy to highlight that the “wastes” of the dissipative process, i.e., CO_2_ and CHCl_3_, do not affect the operation of the dissipative system. After the first ACA dissipative cycle and the associated CO_2_ release in carbonate buffered solutions, the pH differed substantially from the initial value (around pH 8, Figure 1), whereas subsequent TCA cycles restored the system to the same pH. This behavior is likely observed because gaseous CO_2_ saturation had already been reached so that the carbonic anhydride released in subsequent cycles did not significantly affect the system.

Given that the duration of the pH cycles upon successive TCA additions and the period of catalytic inhibition observed by UV/vis do not correlate with the number of performed cycles, the accumulation of chloroform does not appear to affect the system under the conditions employed in the measurements.

### Study of Catalyst Kinetic Behavior

Based on the results shown above, a series of kinetic experiments were carried out in the presence of metal complexes of compounds **1** and **2**. Compound **1** is triazacyclononane (TACN), a macrocyclic polyamine commonly used—along with similar cyclic ligands—to develop metal-based phosphodiesterases due to its strong binding affinity for cations such as Zn^2+^ and Cu^2+^, as well as its inherent chemical reactivity,^37,38^ even in the absence of any functionalization or additive. On the other hand, calix[4]­arene **2**, previously synthesized by our group, was selected due to its well-known efficiency as one of the most effective phosphodiesterases reported in the literature.^37,43^ The significant difference in reactivity between the two compounds—spanning over 3 orders of magnitude under optimal conditions—provides an excellent opportunity for a more in-depth investigation under dissipative conditions. In addition to that, potentiometric titration of the water molecule coordinated to the metal ion indicated a p*K*_a_ value of 8.4 and 8.8 for **1**-Cu^II^ and **2**-Cu^II^, respectively^43^ (see also Supporting Information). These values fall within the pH range observed after the dissipative state, as demonstrated in the potentiometric experiments with TCA shown above.
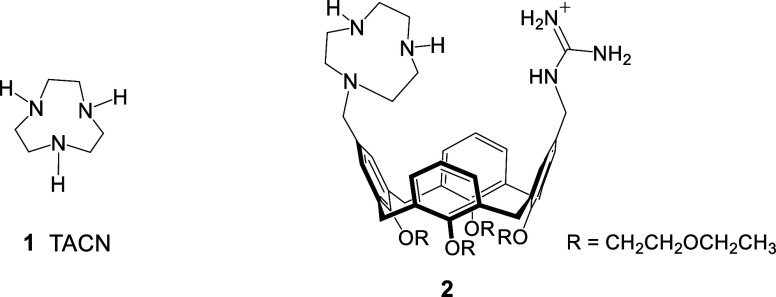


Cu^II^ complexes with TACN and its derivatives have been extensively investigated in previous studies,^37,60–64^ mainly through X-ray analysis, and are known to leave one coordination site available for interaction with water molecule(s). This evidence supports these titration results.

A first set of kinetic experiments was carried out in the same conditions as the potentiometric study, i.e., K_2_CO_3_ in 80% DMSO, in the presence of 2-hydroxypropyl *p*-nitrophenyl phosphate (HPNP). This substrate is a typical RNA model as it features in the 2′ position of the propyl chain a hydroxyl unit able to intramolecularly attack the phosphorus with the formation of a cyclic phosphate according to eq 1. The postulated mechanisms for the cleavage of HPNP promoted by metal complexes of **1** and **2** are depicted in Figure 3. The complexes were obtained by simply mixing CuCl_2_ with TACN and **2** in solution. The thermodynamic and kinetic features of the process ensure their rapid and quantitative formation.
1

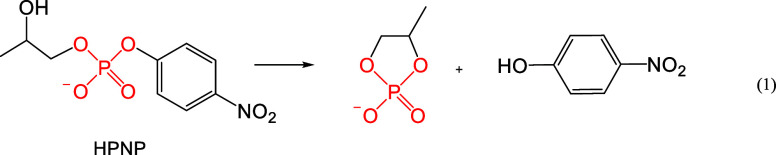




**Figure 3:**
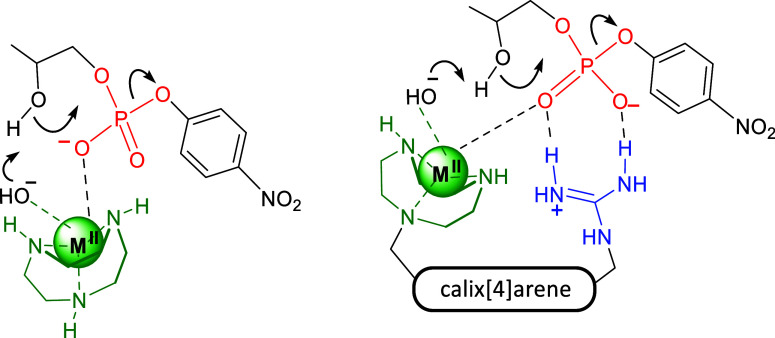
Postulated mono- and bifunctional mechanisms, involving a metal-coordinated hydroxide ion, for the cleavage of HPNP promoted by metal complexes of **1** (left) and **2** (right).

In Figure 4 are reported two kinetic runs of an 80% DMSO solution of 1.0 mM **1**-Cu^II^ initially buffered at pH 9.5 in 10 mM carbonate buffer. The UV–vis monitoring of *p*-nitrophenol liberation at 400 nm was started after the addition of 0.20 mM of HPNP. After approximately 100 min, a first aliquot of TCA was added, resulting in a noticeable decrease in absorbance due to the lower molar absorptivity of *p*-nitrophenol at acidic pH. Nevertheless, a dissipative state in which the catalyst remains inactive was clearly observed after addition of a 10 mM aliquot of acid to the solution (see Figure 4a). This is consistent with our previous study that indicates negligible catalyst activity below pH 6.^43^ The same holds true for subsequent additions of 10 mM TCA (Figure 4a). Conversely, the addition of 5.0 mM TCA led to an initial decrease in absorbance, followed by a rapid increase, without any evidence of a stationary state (Figure 4b). Repeated additions of the same 5.0 mM TCA produced consistent results.

**Figure 4:**
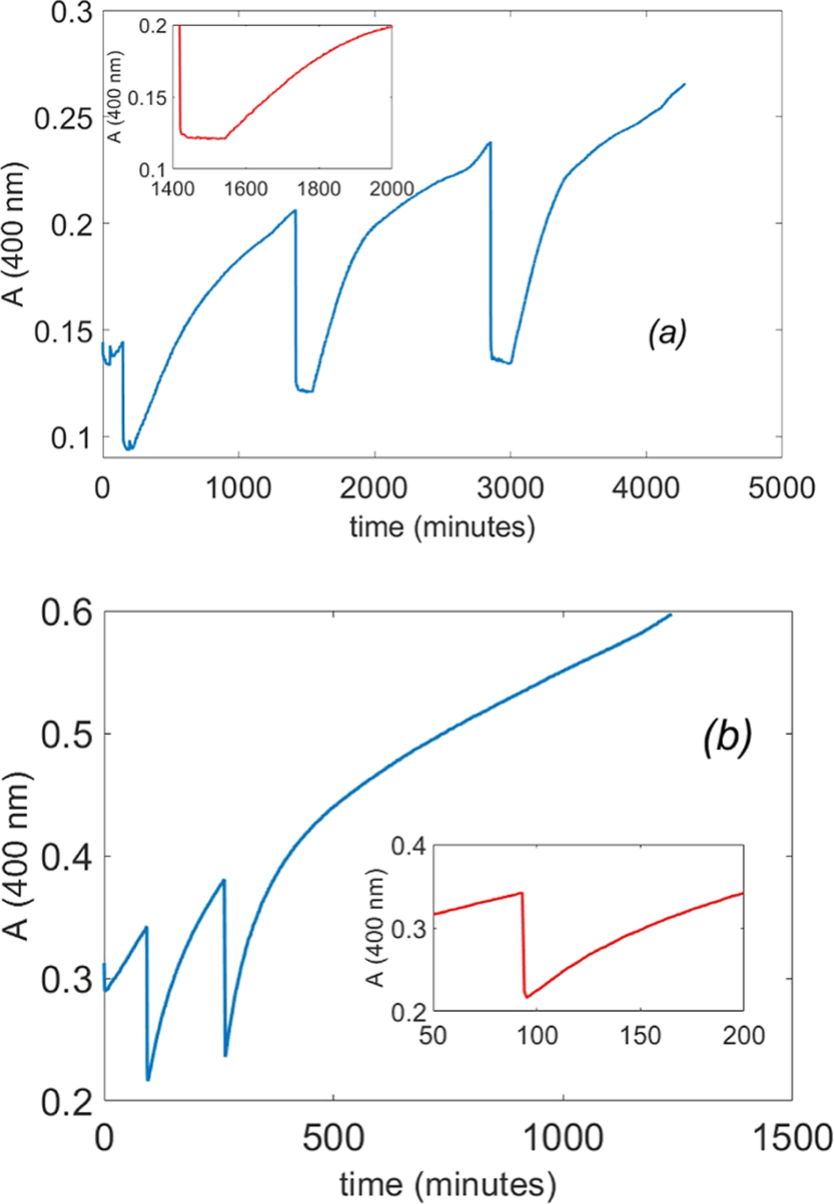
Monitoring of HPNP cleavage by UV–vis spectrophotometry at 400 nm from 1.0 mM solutions of **1**-Cu^II^, 10 mM carbonate buffer, and 0.20 mM HPNP upon subsequent additions of (a) 10 mM TCA and (b) 5.0 mM TCA. *T* = 25 °C in 80% DMSO. The insets show selected portions of the plots enlarged to highlight the shape of the curve during the dissipative phases.

An alternative approach involves starting from a solution pretreated with TCA and adding the substrate as the final component, rather than starting from a catalytically active state. Figure 5 shows the absorbance profiles for three different solutions containing 2.0 mM **1**-Cu^II^ and 10–20 mM TCA in 10 mM carbonate buffer. The acid additions were performed without prior pH adjustment. Data acquisition started after the addition of 0.20 mM HPNP as the final component. A clear absence of the dissipative state is observed at the lowest TCA concentration, whereas in the other two cases a dissipative state lasting approximately 23 and 40 min is evident (see Figure 5). These results clearly demonstrate that temporal control of the catalytic activity is achieved, which allows for programmed activation of the catalyst at a predetermined time.

**Figure 5:**
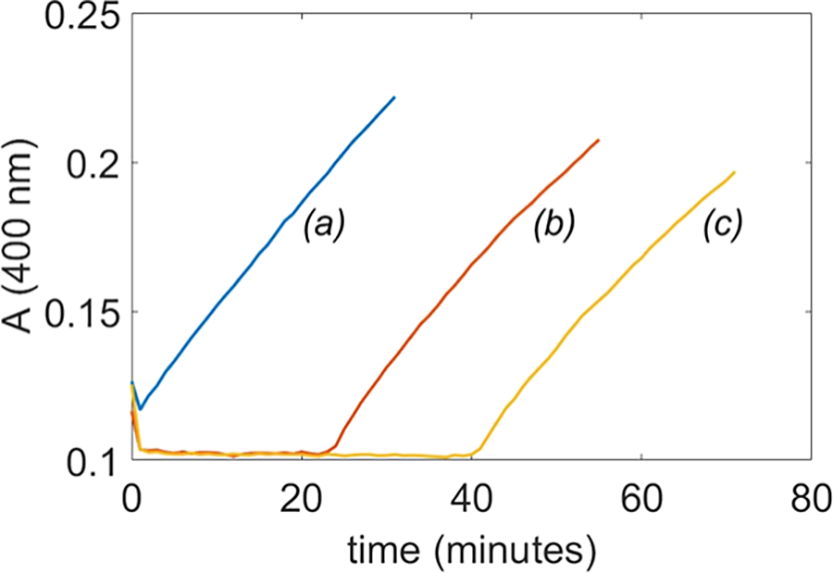
UV–vis monitoring of the cleavage of 0.20 mM HPNP at 400 nm in the presence of 2.0 mM **1**-Cu^II^, from three distinct solutions pretreated with 10 mM (a), 15 mM (b), and 20 mM (c) TCA. Reactions performed in 80% DMSO, 10 mM carbonate buffer, *T* = 25 °C.

It is noteworthy, as also supported by additional experiments, that increasing the catalyst concentration leads to a shorter duration of the dissipative state. This behavior is likely attributable to the same effect observed upon variation of the carbonate buffer concentration.

We focused on formulating a chemical kinetic model expressed in terms of the concentrations of the involved species. However, the decarboxylation rate is influenced by all species present in solution—particularly charged ones—in a non-negligible way. Consequently, the catalyst itself alters the decarboxylation kinetics, and the rate measured in its presence is intrinsically different from that observed in its absence. Moreover, the detection of the reaction product, *p*-nitrophenol, is pH-dependent: its protonation state—and therefore its extinction coefficient—varies with pH. This effect overlaps with the overall catalytic process and further complicates the kinetic analysis. For these reasons, deriving a simple time-dependent rate expression that explicitly includes the initial concentrations of all species is not feasible without introducing assumptions or strong approximations.

For a more comprehensive investigation, we have studied the catalytic behavior of the Cu^II^ complex of calix[4]­arene **2**, which showed a significantly higher catalytic efficiency than the bare TACN (**1**) metal complex.

In the presence of compound **1**-Cu^II^, a substrate concentration of 0.20 mM allowed approximately 10% of the reaction conversion to be monitored over several hours. On the other hand, for the Cu^II^ complex of compound **2**, a lower substrate concentration of 50 μM was employed, which enabled monitoring of a high percentage of reaction while avoiding excessively high absorbance values. In Figure 6, the cleavage of the RNA model compound HPNP is shown in the presence of the metal complex of bifunctional calix[4]­arene **2**. The figure compares two experiments carried out under the same conditions, differing only in the amounts of TCA added.

**Figure 6:**
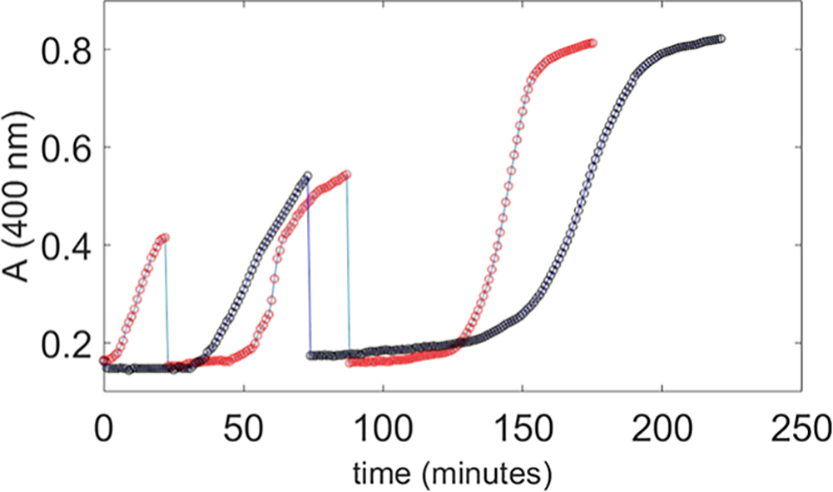
UV–vis absorption profiles at 400 nm of a 50 μM HPNP solution containing 1.0 mM **2**-Cu^II^, recorded after two sequential additions of TCA: 10 mM (red, three subsequent aliquots added) and 20 mM (black, two subsequent aliquots added). Conditions: 80% DMSO, 10 mM carbonate buffer, *T* = 25 °C.

Consistently with previous kinetic experiments, no preliminary pH adjustment was made. The first TCA addition was carried out after all other mixture components were introduced and immediately before the addition of the substrate. The kinetic monitoring started immediately after the addition of HPNP addition. In the presence of 10 mM TCA, no dissipative state is initially observed; instead, a gradual increase in absorbance occurs within the first few minutes, indicating an immediate, albeit progressive, activation of the catalyst. However, in following subsequent additions of the same amount of TCA, a dissipative state becomes evident and persists for approximately 30 min. In contrast, with 20 mM TCA, a dissipative state is already observed at the beginning of the UV–vis monitoring, and the second dissipative phase is again significantly prolonged, likely for the same reasons mentioned above. Notably, in this case, the absorbance versus time profile reaches a plateau after the second addition, indicating that the reaction proceeded nearly to completion, with an extent of conversion approaching 85–90% in about 3 h. This was made possible by the markedly enhanced reactivity of the bifunctional catalyst **2**-Cu^II^.

It is remarkable to note that in this solvent mixture, potentiometric titrations carried out in previous studies^43,65^ have allowed the determination of all the species present in solution. From these potentiometric experiments, we proved that the binding constant of **2** with Cu^II^ is extremely high (Log*K*_Cu_ = 9.2) and even higher for TACN (see Supporting Information Section S6). With such a high binding constant, we can conclude that at millimolar concentration or submillimolar concentration, the complex is completely formed. From the titration experiments, we know that the p*K*_a_ of the coordinated water molecule is 8.4 and 8.8 for TACN and compound **2**, respectively. These values fall in a range that is very close to the pH value of the carbonate buffer solution after the recovery from the dissipative state. This is a reason why the carbonate buffer is particularly suited for our purposes. Consequently, after the recovery there is a significant amount of active catalyst, i.e., the species featuring a coordinated hydroxide ion. From the titration curves mentioned above, we can also argue that the complex with Cu^II^ is formed even at low pH values. Basically, there is never a dissociation of the metal under the conditions of the experiments carried out in this study.

A further relevant point is that no evidence of catalyst degradation is observed. This is supported by the fact that the previously reported titration studies^43^ are reproducible and consistent and that the kinetic measurements performed both in this work and in earlier studies^43,66^ did not show any deviations from linearity in the plots of *k*_obs_ versus catalyst concentration.

## Conclusions

In summary, in this study, we were able to control the activation of the catalysis offered by a supramolecular catalyst in a dissipative fashion. In the first part of the investigation, the behavior of the ACA was studied in the solvent mixture suitable for potentiometric investigation and kinetic measurements. In these experiments, several significant findings emerged. First, the existence and duration of the dissipative state depend on the amount of TCA added. With the exception of the first addition of acid, which generates a shorter dissipative state, subsequent additions result in a constant and reproducible duration of the dissipative state, even for further additions to the same solution. The reason for this observation is that carbon dioxide produced during the decarboxylation step alters the ratio between the acidic and basic components of the buffer until a saturation value is reached. Moreover, the experiments indicate that the concentrations of buffer and catalyst affect the duration of the dissipative state by reducing it.

The kinetic analysis of the cleavage rate of the model compound HPNP, in the presence of the two artificial phosphodiesterases under consideration, clearly indicates the possibility of achieving temporal control over catalyst activation. This control can be exercised from either a deactivated or an activated state, corresponding to low and high pH values.

The findings presented in this work offer a contribution to the studies aimed at reproducing the behavior of reactions in living systems where enzymes and catalysts are frequently activated or deactivated by transient chemical signals. This approach prevents uncontrolled or constant activity, allowing for the fine, programmable, and reversible control of the catalytic function.

## Experimental Section

### Instruments

The pH measurements were carried out with an XS-Securelab GB50101102 pH meter provided with a 201 T DHS electrode. Since the electrode was used in 80% DMSO, a proper calibration procedure is necessary to obtain reliable measurements, *vide infra*. All kinetic runs described were monitored by a Shimadzu UV-2450 UV–vis Spectrophotometer Shimadzu UV-2450.

### Materials

Commercial DMSO RPE-ACS—Carlo Erba—was purged for 30 min with argon. Ultrapure mQ water for high-performance liquid chromatography was used to prepare the semiaqueous 80% DMSO employed in the kinetic and potentiometric experiments. TCA was supplied by Merck as a 6.1 N solution in water. HPNP was prepared as a barium salt according to a protocol reported in literature.^67^ The synthesis of calix[4]­arene **2** was carried out according to a literature procedure previously reported by our group.^43^ TACN was purchased from Merck. All other solvents and reagents were used as commercially available without any further purification.

### Potentiometric Measurements

Potentiometric measurements of the solution under the dissipative state were performed by the pH meter indicated above. The electrode was calibrated using a buffer solution whose pH is known from previous literature investigations^58^ carried out in the same solvent mixture. The time required to obtain a stable pH reading ranges from 2 to 6 min. The calibration plot of the real pH value (pH_corr_) versus experimental pH readings was linear in the investigated range. The real pH value was obtained from the following equation: pH_corr_ = – log­[H^+^] = *a* + *b* × pH_read_. The best fit values for the parameters are *a* = −0.3244 ± 5% and *b* = 0.968 ± 5%. The plot is reported in Section S4 of the Supporting Information. This calibration procedure is based on buffer solutions whose p*K*_a_ values had been previously determined in the literature^55^ under the exact same conditions used in the present work, i.e., mesitol, *p*-chlorophenol, *p*-cianophenol, acetic acid, and dichloroacetic acid. The plot mentioned above shows a linear trend. In the potentiometric experiments, a potassium carbonate solution (5–20 mM) was prepared by dissolving the salt in water and then adding dropwise under stirring the proper amount of DMSO to achieve an 80% v/v concentration. The commercial TCA solution was added directly to a 6 mL volume of this mixture, typically in a quantity of 5–10 μL, to achieve the acid concentration reported in the main text, 5–20 mM TCA. The additions were carried out with a 10 μL Hamilton syringe. In some of the experiments described in the text, the solution pH was adjusted using a 1.0 M solution of HClO_4_ prepared by carefully adding typical additions of commercial HClO_4_ ranging from 5 to 15 μL to the same ice-cooled semiaqueous mixture. Warning! The dilution of perchloric acid in DMSO is highly exothermic and can produce dangerous splashes. The operation was carried out in a fume hood with the appropriate personal protective equipment. No accident occurred in the course of the present work. In other experiments, no preadjustment of the pH was performed. After the final addition of TCA, the pH values were manually recorded at appropriate time intervals, depending on the rate of pH change. The durations of the dissipative states reported in the figures and discussed in the main text were determined from the time of the TCA addition to the inflection point of the curve. In the case where no inflection point of the curve is observed (e.g., Figure S1.1), the duration is set to zero (see Figure 2a).

### UV–Vis Spectrophotometric Experiments

The transesterification of HPNP was monitored through the formation of *p*-nitrophenol, see eq 1. The absorbance increase was followed at 400 nm. The absorbance of the solution at that wavelength also depends on the pH, as the *p*-nitrophenol/*p*-nitrophenolate ratio varies significantly across the investigated pH range. A spectrophotometric titration of *p*-nitrophenol affords the following molar extinction coefficients: ε_pNPhOH_ = 87.1 cm^–1^ M^–1^ and ε_pNPhO_ = 19040 cm^–1^ M^–1^ for *p*-nitrophenol and *p*-nitrophenolate, respectively. Based on these coefficients at 400 nm, a p*K*_a_ value of 8.02 was calculated. The kinetic runs were followed for a small reaction percentage of 5–10% for the catalytic measures in the presence of TACN-Cu^II^. On the contrary, in the presence of complex **2**-Cu^II^, a significant reaction percentage was followed. For the kinetic measurements, the durations of the stationary states reported in the main text were determined from the time of TCA addition to the point at which the absorbance value ceases to remain constant. In cases where no stationary region is observed (e.g., Figure 4b), the duration was set to zero.

The raw data of the measurements are deposited in Section S5 of the Supporting Information. The elaboration of the experimental data and the plots reported in the manuscript were performed using the MathWorks software package MATLAB, either version R2022a (9.12.0.1956245, 64 bit) or version R2025a (25.1.0.2943329, 64 bit).

## Supplementary Material



## Notes

Additional results are available including the following: plots of potentiometric experiments, plots of UV–vis spectrophotometry experiments, plots of the kinetic experiments, details about the pH meter calibration, and all the numeric raw data about the kinetic and potentiometric experiments (PDF)
