# Supplementary material for: Temporally Controlled Supramolecular Catalysts with pH-Dependent Activity
Source: ACS Omega. 2026 Jan 22;11(4):6353–61. doi: 10.1021/acsomega.5c11122 (PMC12878715; doi:10.1021/acsomega.5c11122)
Supplement: Supplementary file 1 [file ao5c11122_si_001.pdf]

Supporting Information for

Temporally Controlled Supramolecular Catalysts with pH-Dependent Activity

Giulio Pucciarelli,[a ≠] Francesco Ranieri,[b ≠] Alessandro Casnati[c] , Stefano Di Stefano,[b,d] * Stefano Volpi[c]* and Riccardo Salvio[a,d] *

[a] *Dipartimento di Scienze e Tecnologie Chimiche, Università "Tor Vergata", Via della Ricerca Scientifica, 1, 00133 Roma, Italy*

[b] *Dipartimento di Chimica, Università di Roma La Sapienza, P.le A. Moro 5, 00185, Roma, Italy*

[c] *Dipartimento di Scienze Chimiche, della Vita e della Sostenibilità Ambientale, Università degli Studi di Parma, Parco Area delle Scienze, 17/A, 43124, Parma, Italy*

[d] *ISB – CNR Sezione Meccanismi di Reazione, Università La Sapienza, P.le A. Moro 5, 00185 Roma, Italy.*

* To whom the correspondence should be addressed: riccardo.salvio@uniroma2.it, stefano.distefano@uniroma1.it , stefano.volpi@unipr.it.

≠ these authors equally contributed to the present work.

Table of Contents

S1 Potentiometric Experiments...p. S3

S2 Decarboxylation of TCA monitored with UV-Vis Spectrophotometry ....p. S21

S3 Kinetic Experiments in the presence of the Catalysts ....p. S22

S4 pH meter calibration in 80% DMSO ....p. S25

S5 Appendix 1 - Raw Potentiometric and UV-Vis Data ....p. S26

S6 Appendix 2 – Potentiometric titrations from previous investigations………………………p. S209

Section S1 Potentiometric Experiments

In the following pages are reported the plots and the experimental details of the potentiometric experiments carried out in the absence of any catalysts. Initial pH is adjusted with small aliquots of semi-aqueous $HClO_4$ 1.0 M, unless otherwise specified. Further details about the experiments and the definition of the DS duration are reported in the main text and in the Experimental Section of the article. In these experiments the pH read on the instrument is reported. In order to convert it to the real pH see the equation in the Experimental Section.

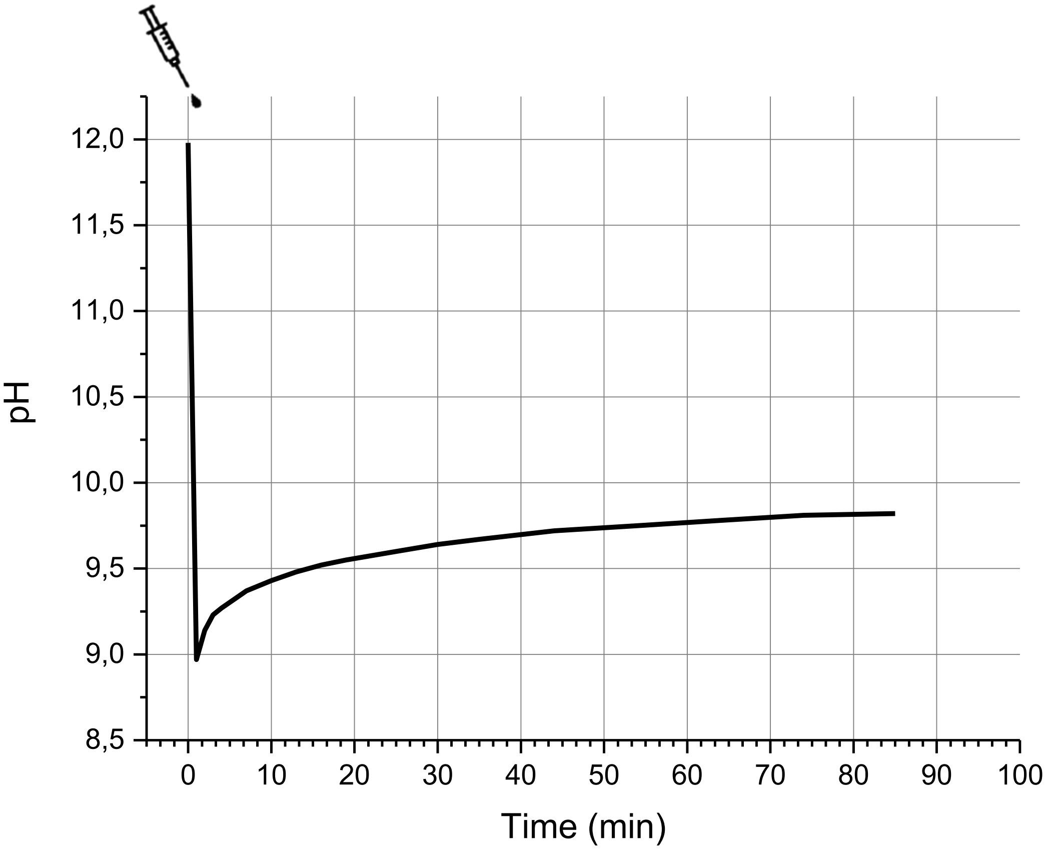

Figure S1.1

TCA inj. #	Initial pH	pH after TCA	pH plateau	DS duration (min)	[TCA] (mM)	[K_2CO_3] (mM)
1	11.98	8.97	9.82	n/a	5	10

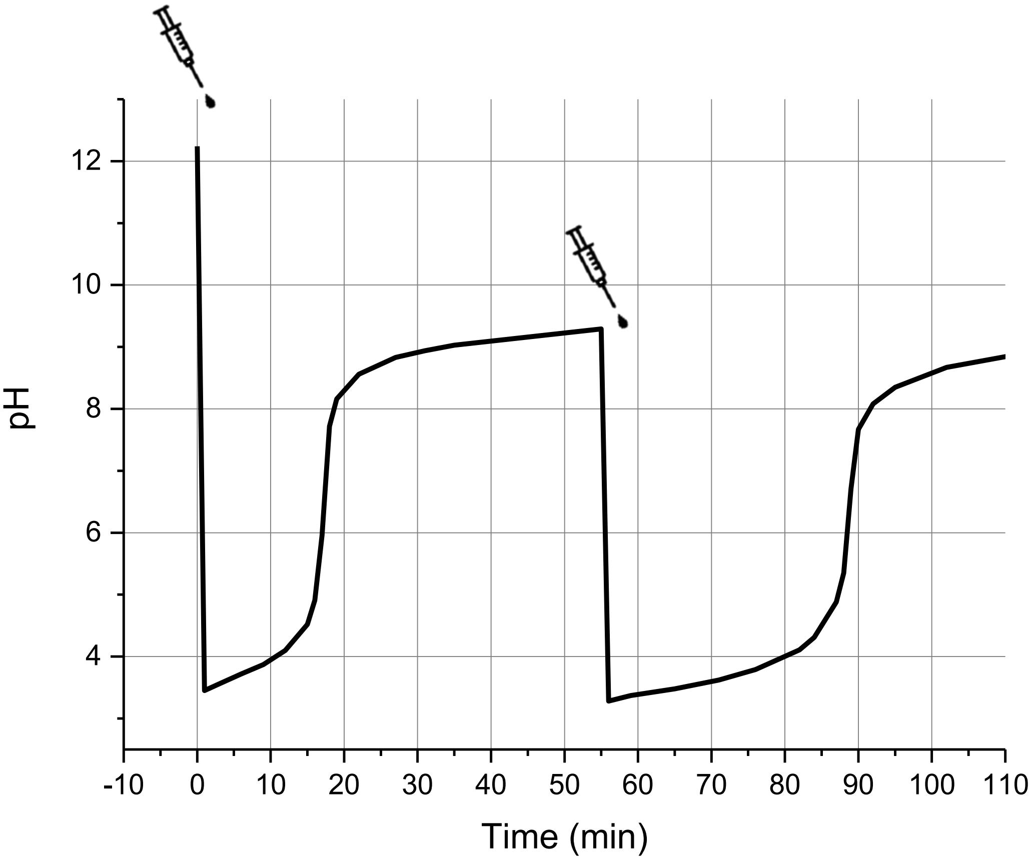

Figure S1.2

TCA inj. #	Initial pH	pH after TCA	pH plateau	DS duration (min)	[TCA] (mM)	[K_2CO_3] (mM)
1	12.24	3.45	9.29	14	7.5	10
2	9.29	3.28	8.95	30	7.5	10

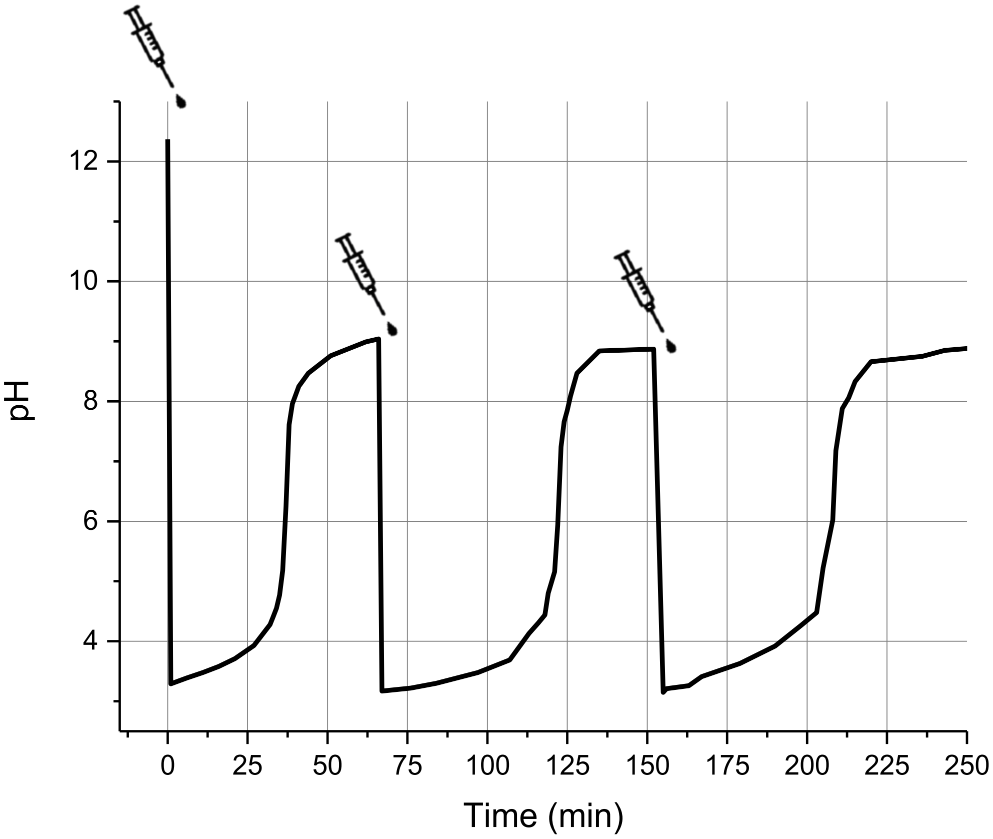

Figure S1.3

TCA inj. #	Initial pH	pH after TCA	pH plateau	DS duration (min)	[TCA] (mM)	[K_2CO_3] (mM)
1	12.37	3.29	9.04	33	10	10
2	9.04	3.17	8.87	50	10	10
3	8.87	3.15	8.9	48	10	10

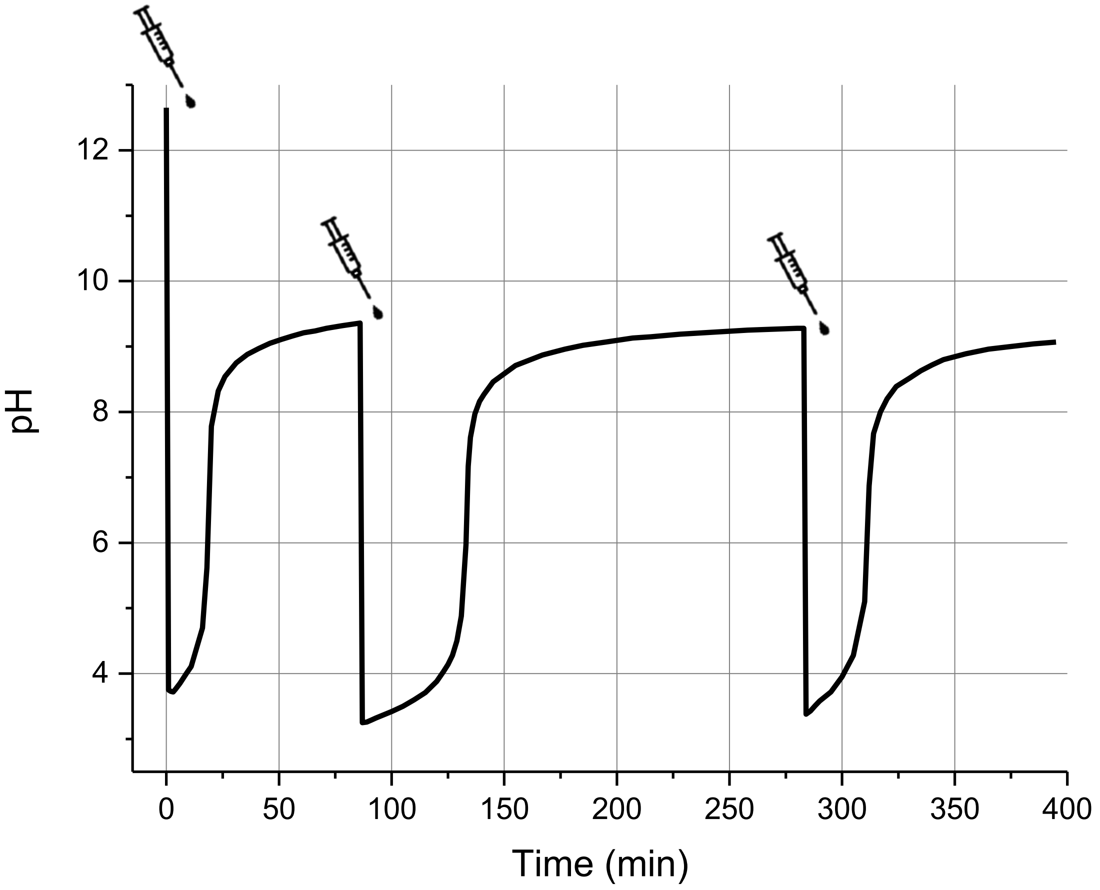

Figure S1.4

TCA inj. #	Initial pH	pH after TCA	pH plateau	DS duration (min)	[TCA] (mM)	[K_2CO_3] (mM)
1	12.65	3.72	9.36	15	10	10
2	9.36	3.25	9.28	43	10	10
3	9.28	3.38	9.07	26	10	10

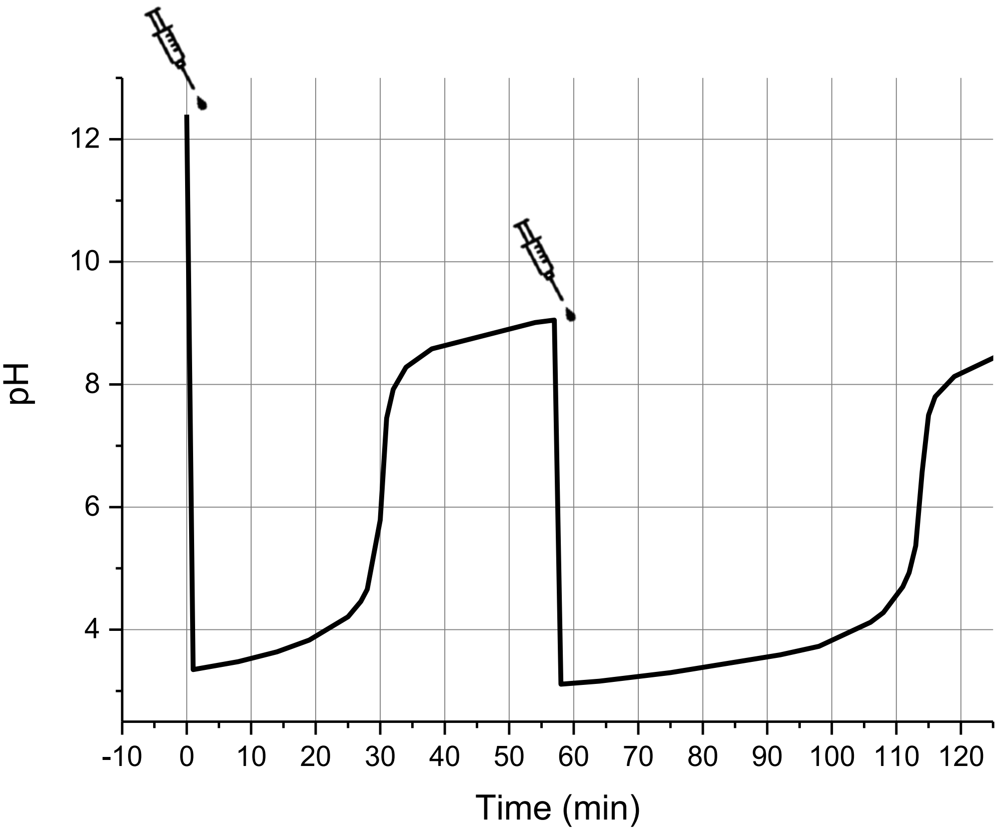

Figure S1.5

TCA inj. #	Initial pH	pH after TCA	pH plateau	DS duration (min)	[TCA] (mM)	[K_2CO_3] (mM)
1	12.4	3.35	9.05	27	15	10
2	9.05	3.11	8.66	52	15	10

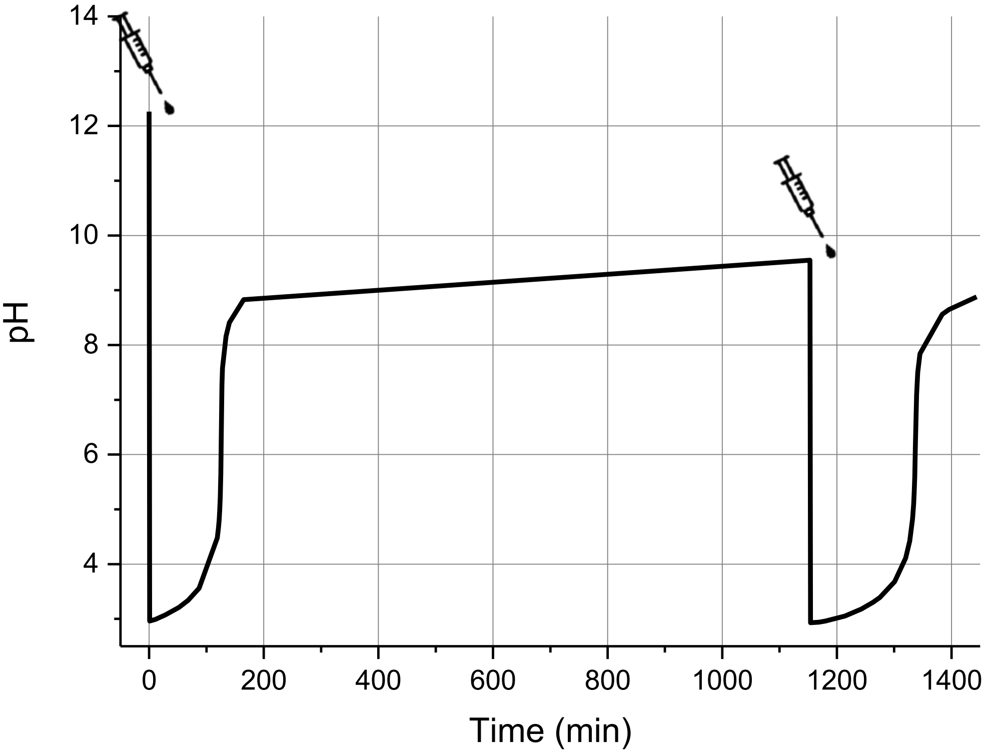

Figure S1.6

TCA inj. #	Initial pH	pH after TCA	pH plateau	DS duration (min)	[TCA] (mM)	$[K_2CO_3]$ (mM)
1	12.26	2.96	9.55	124	20	10
2	9.55	2.93	8.65	182	20	10

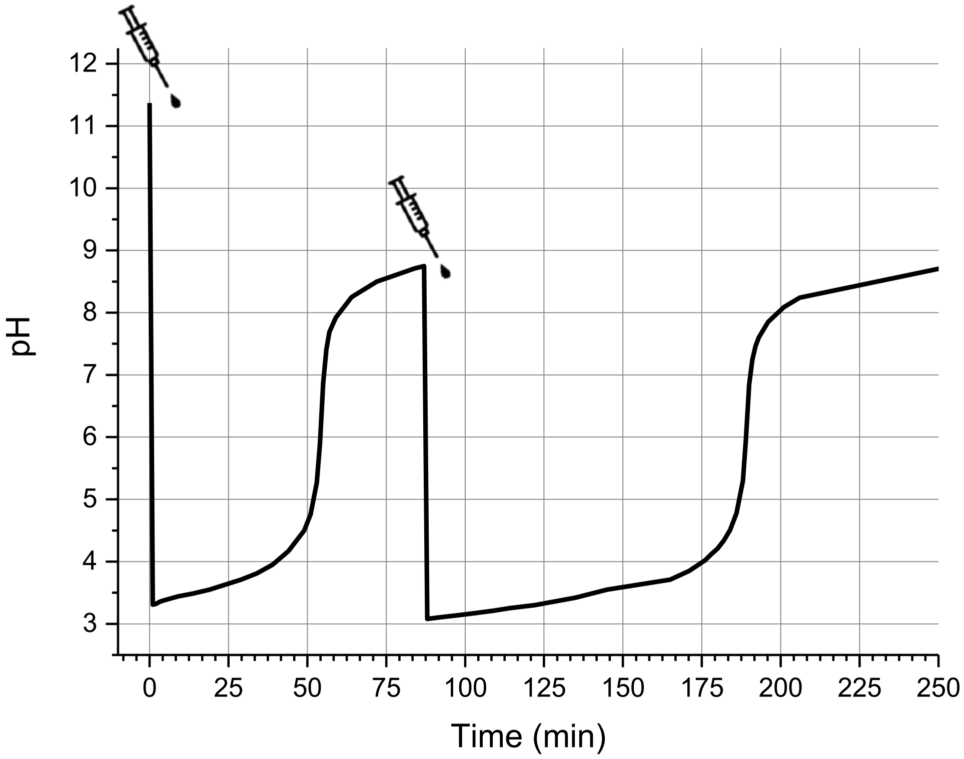

Figure S1.7

TCA inj. #	Initial pH	pH after TCA	pH plateau	DS duration (min)	[TCA] (mM)	[K_2CO_3] (mM)
1	11.37	3.31	8.75	50	10	10
2	8.75	3.08	8.77	97	10	10

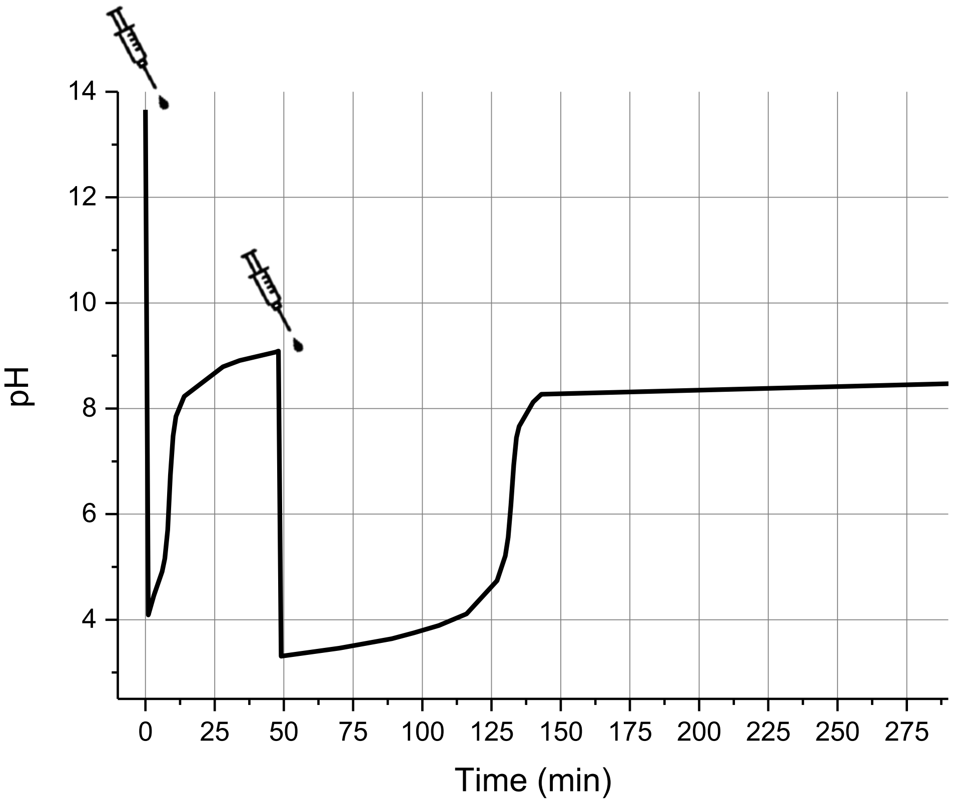

Figure S1.8

TCA inj. #	Initial pH	pH after TCA	pH plateau	DS duration (min)	[TCA] (mM)	[K_2CO_3] (mM)
1	13.66	4.09	9.09	7	10	10
2	9.09	3.31	9.49	81	10	10

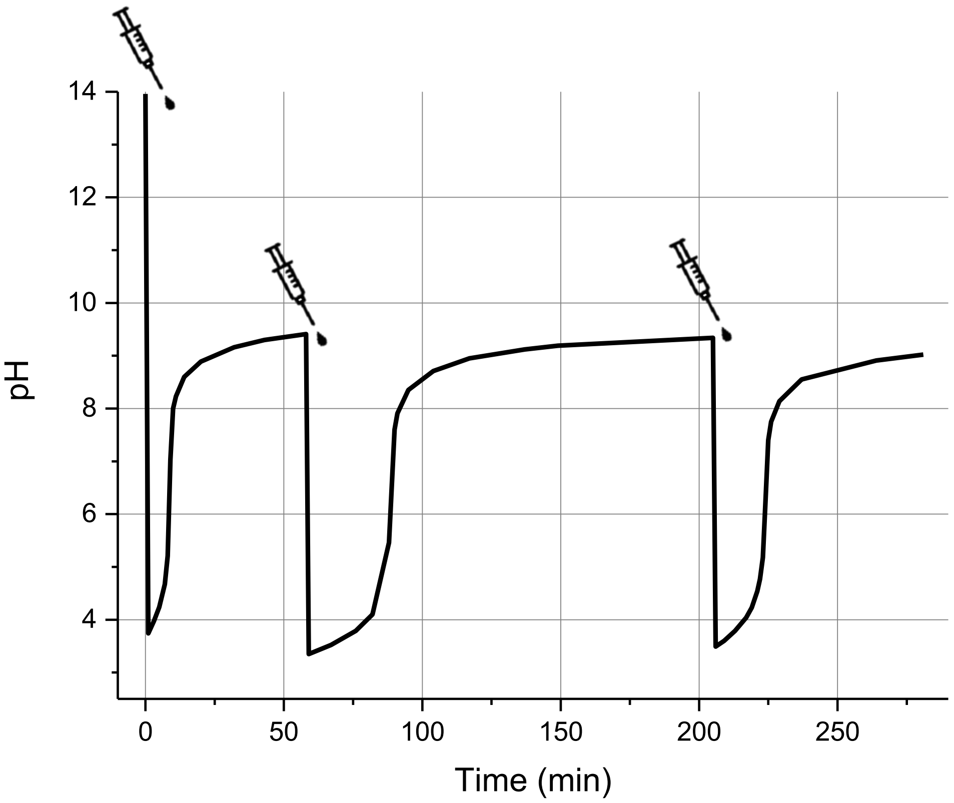

Figure S1.9

TCA inj. #	Initial pH	pH after TCA	pH plateau	DS duration (min)	[TCA] (mM)	[K_2CO_3] (mM)
1	13.96	3.74	9.41	8	10	10
2	9.41	3.35	9.34	31	10	10
3	9.34	3.35	9.12	20	10	10

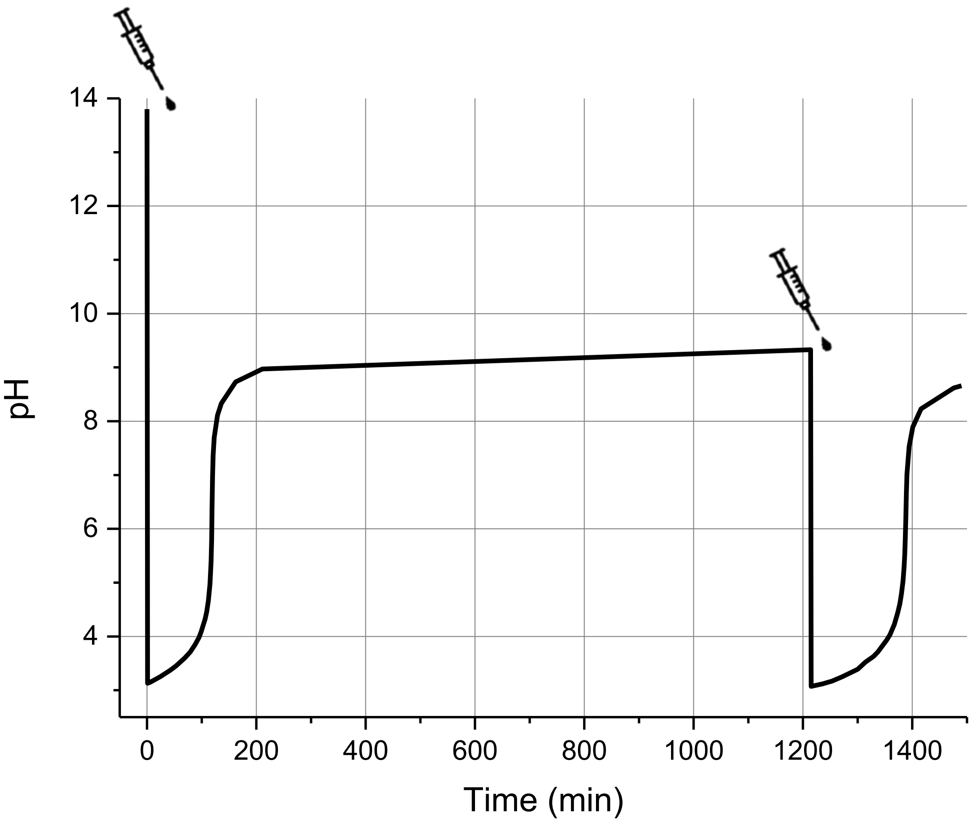

Figure S1.10

TCA inj. #	Initial pH	pH after TCA	pH plateau	DS duration (min)	[TCA] (mM)	[K_2CO_3] (mM)
1	13.8	3.13	9.33	115	10	5
2	9.33	3.07	8.66	168	10	5

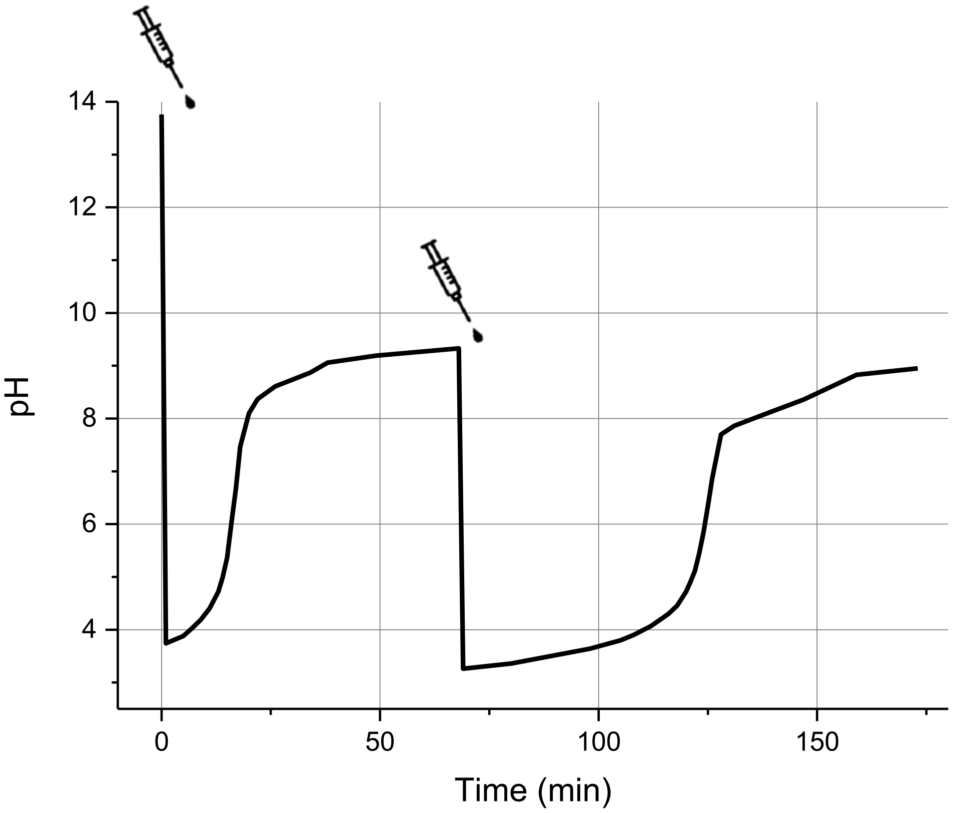

Figure S1.11

TCA inj. #	Initial pH	pH after TCA	pH plateau	DS duration (min)	[TCA] (mM)	[K_2CO_3] (mM)
1	13.76	3.74	9.33	13	10	15
2	9.33	3.26	8.95	54	10	15

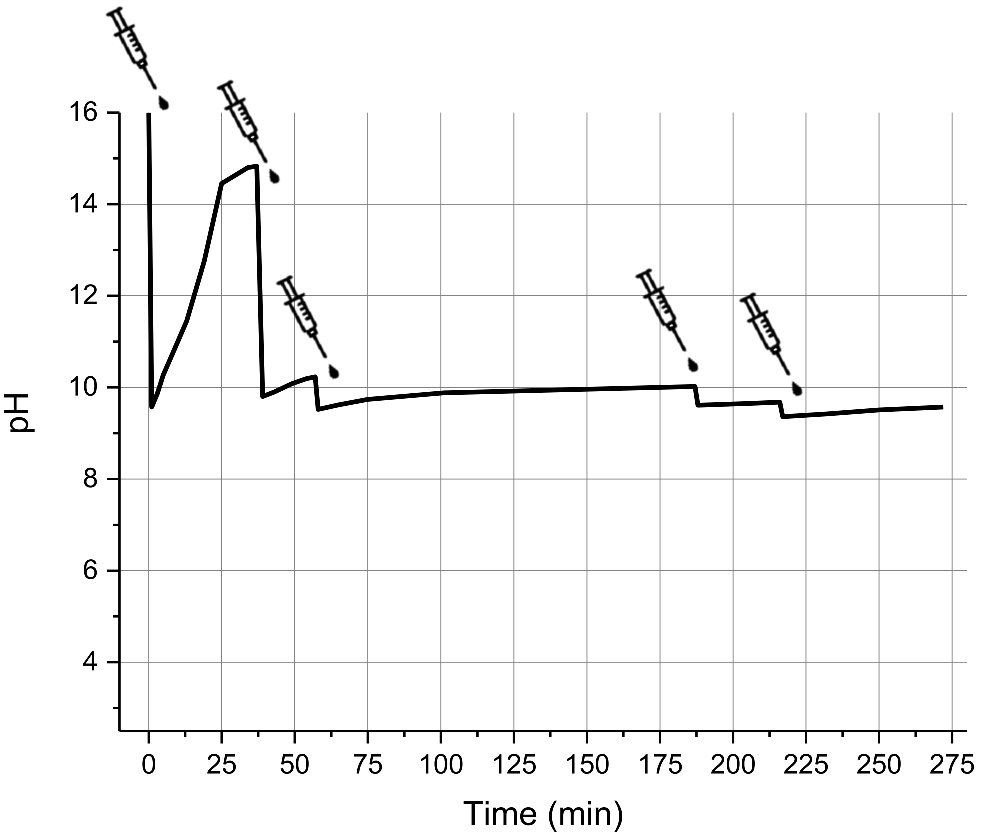

Figure S1.12

TCA inj. #	Initial pH	pH after TCA	pH plateau	DS duration (min)	[TCA] (mM)	[K_2CO_3] (mM)
1	16 [a]	9.57	14.83	n/a	5	10
2	14.83	9.8	10.23	n/a	5	10
3	10.23	9.52	10.02	n/a	5	10
4	10.02	9.61	9.68	n/a	5	10
5	9.68	9.36	9.57	n/a	5	10

[a] No pH pre-adjustment.

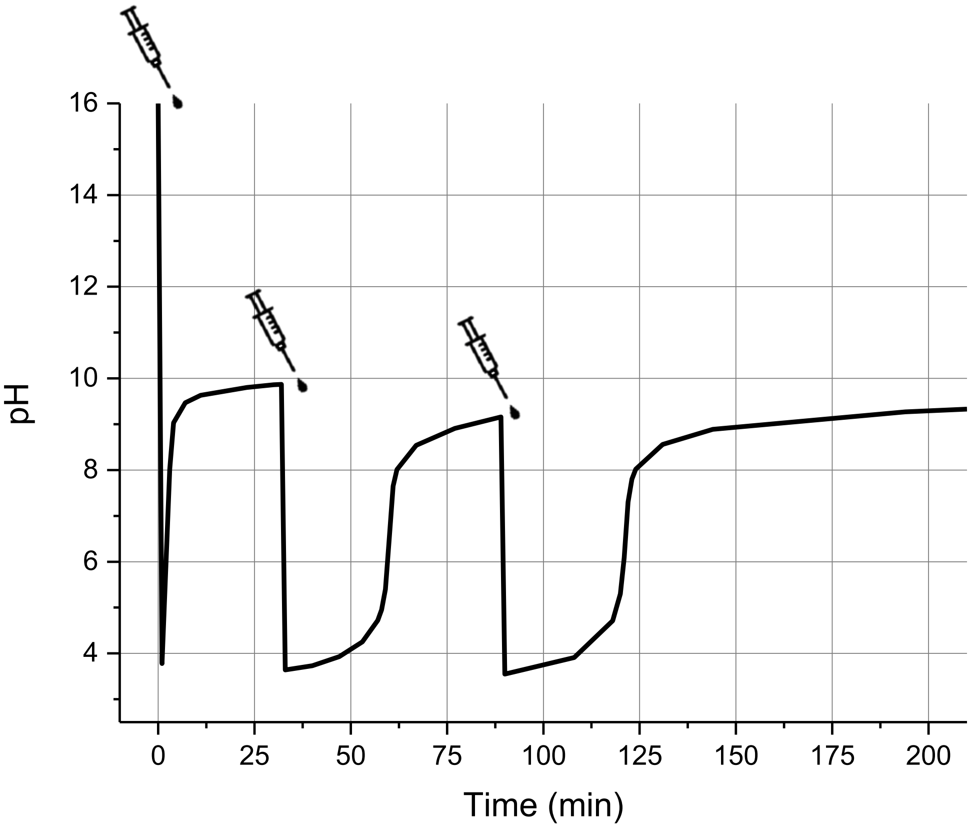

Figure S1.13

TCA inj. #	Initial pH	pH after TCA	pH plateau	DS duration (min)	[TCA] (mM)	[K_2CO_3] (mM)
1	16	3.78	9.87	1	7.5	10
2	9.87	3.64	9.16	26	7.5	10
3	9.16	3.55	9.33	30	7.5	10

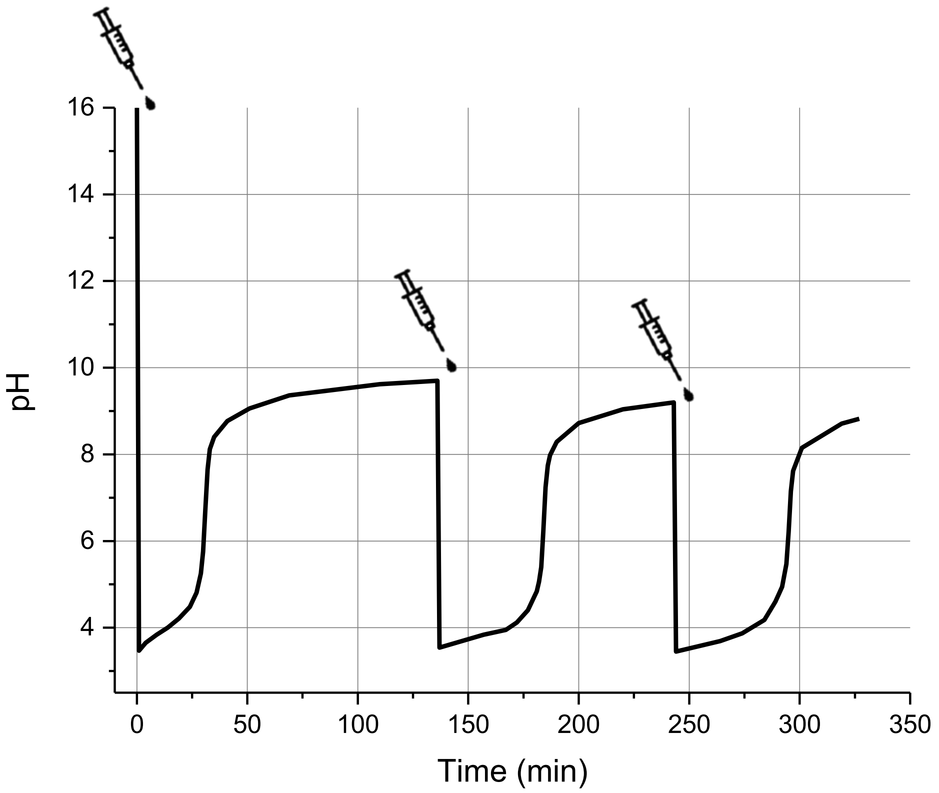

Figure S1.14

TCA inj. #	Initial pH	pH after TCA	pH plateau	DS duration (min)	[TCA] (mM)	[K_2CO_3] (mM)
1	16	3.47	9.7	29	10	10
2	9.7	3.54	9.2	47	10	10
3	9.2	3.45	9.39	51	10	10

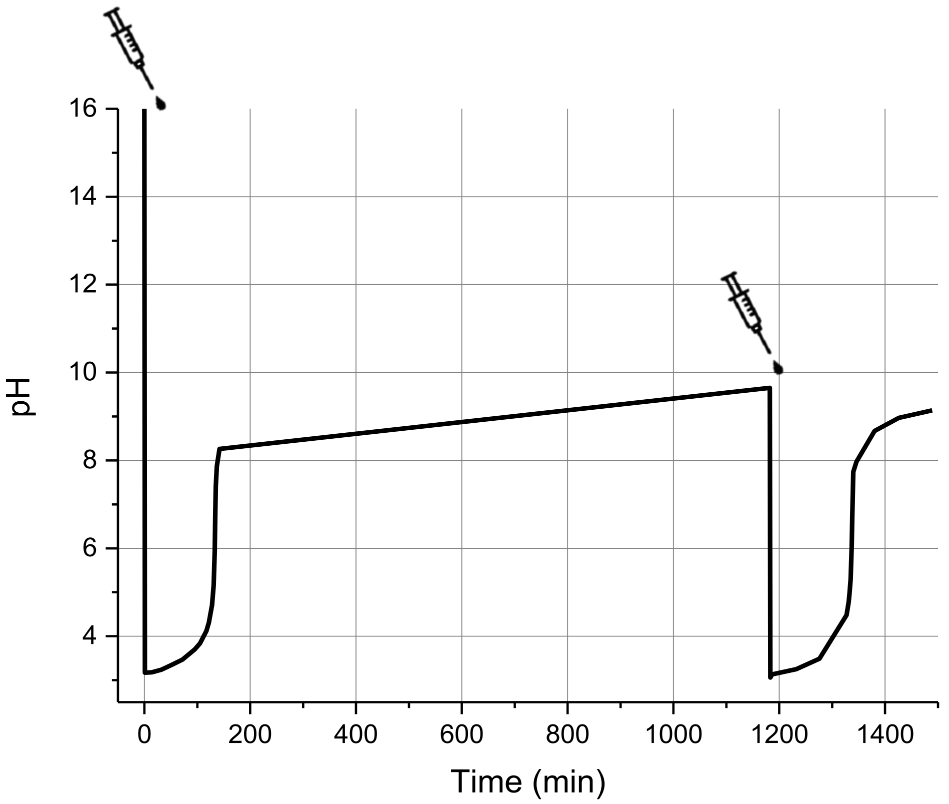

Figure S1.15

TCA inj. #	Initial pH	pH after TCA	pH plateau	DS duration (min)	[TCA] (mM)	[K_2CO_3] (mM)
1	16	3.17	9.65	131	20	10
2	9.65	3.06	9.14	153	20	10

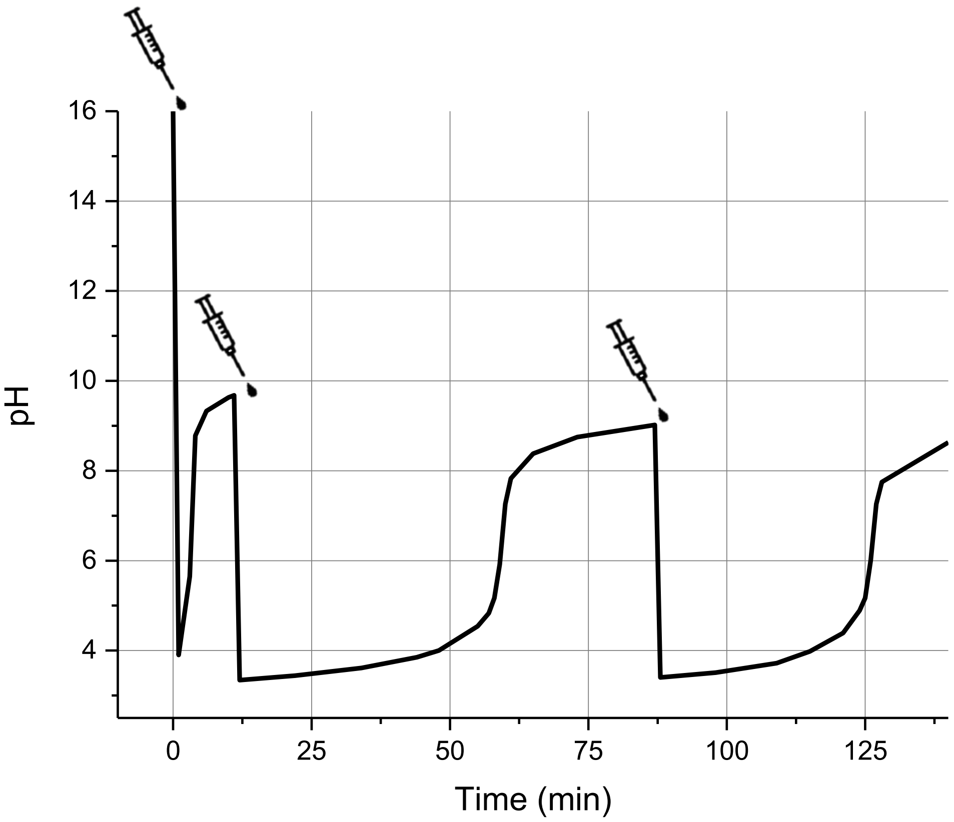

Figure S1.16

TCA inj. #	Initial pH	pH after TCA	pH plateau	DS duration (min)	[TCA] (mM)	[K_2CO_3] (mM)
1	16	3.9	9.68	2	7.5	10
2	9.68	3.34	9.02	46	10	10
3	9.02	3.4	8.63	38	8.75	10

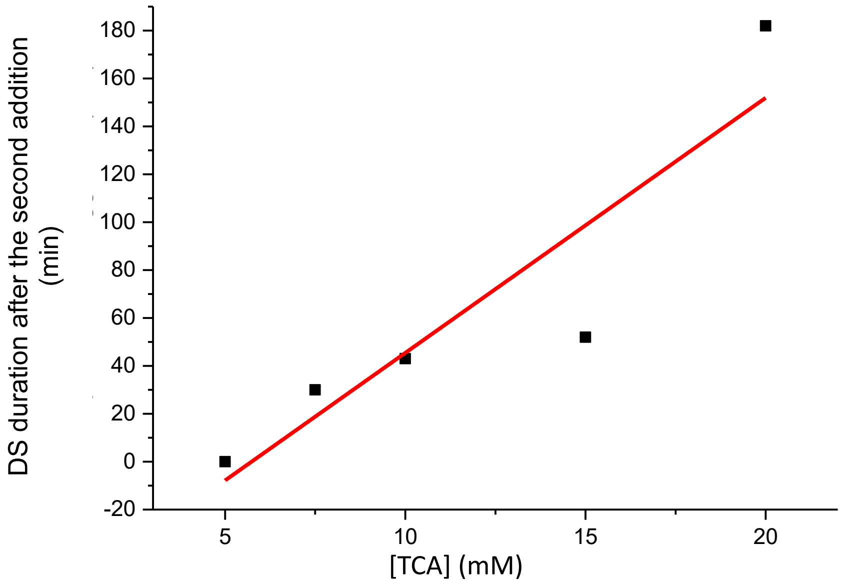

Figure S1.17 Plot of the duration of the dissipative state after the second addition of TCA versus the TCA concentration. In the corresponding experiments [K_2CO_3] = 10 mM The solution was pre-adjusted with $HClO_4$ at an approximate value of/ at about pH 12.5.

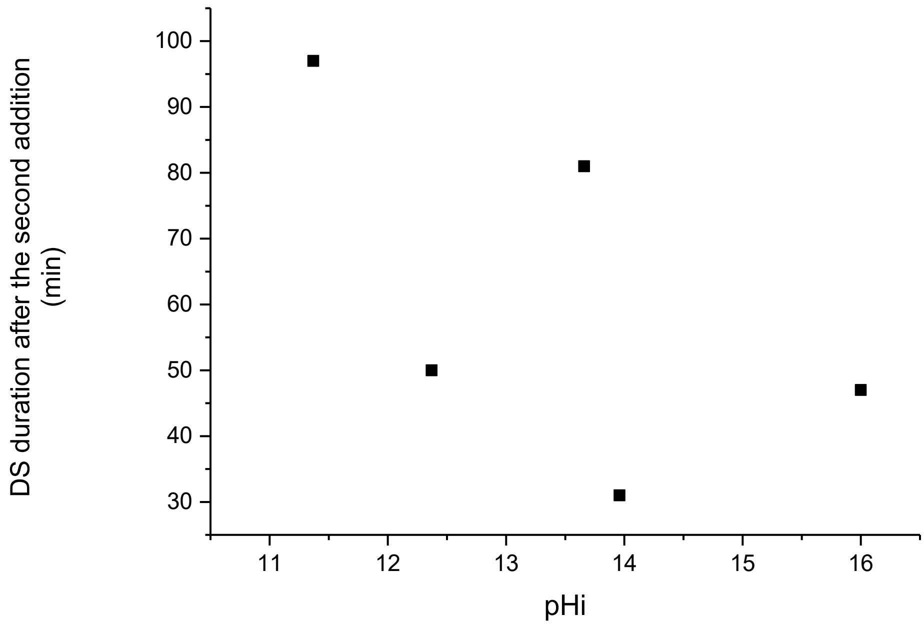

Figure S1.18 Plot of the duration of the dissipative state after the second addition of TCA versus the initial pH pre-adjusted with $HClO_4$ / the plateau pH at the end of the first dissipative cycle. [K_2CO_3] = 10 mM and [TCA] = 10 mM.

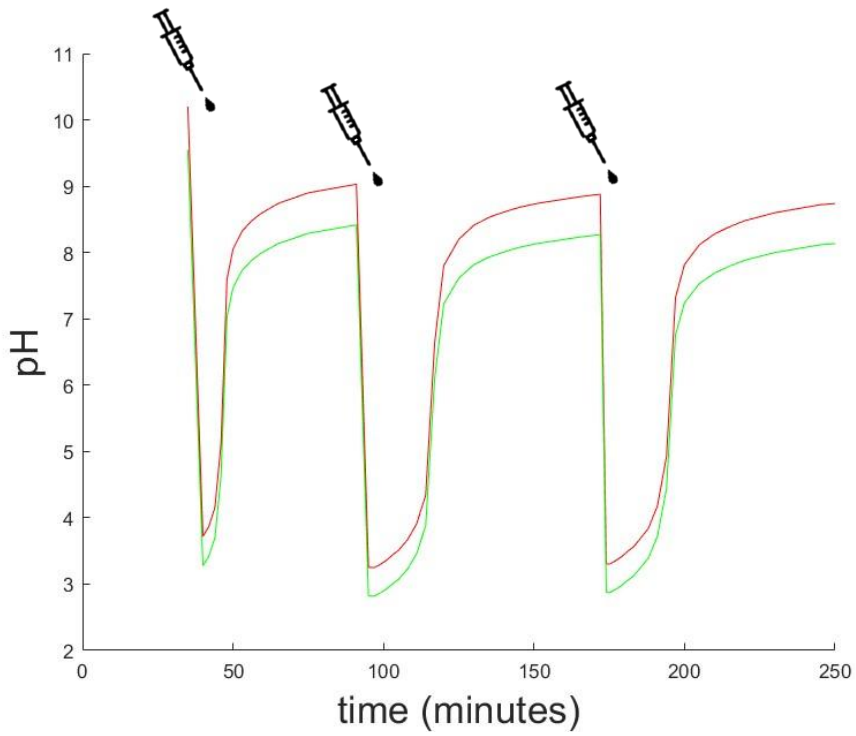

Figure S1.19 Plot of uncorrected (red) and corrected (green) pH. The pH values reported in the table below are corrected ones.

TCA inj. #	Initial pH	pH after TCA	pH plateau	DS duration (min)	[TCA] (mM)	[K_2HPO_4] (mM)
1	10.20	*3.72*	9.03	8	7.5	10
2	9.03	3.25	8.88	22	10	10
3	8.88	3.31	8.74	22	8.75	10

Section S2 Decarboxylation of TCA monitored with UV-Vis Spectrophotometry

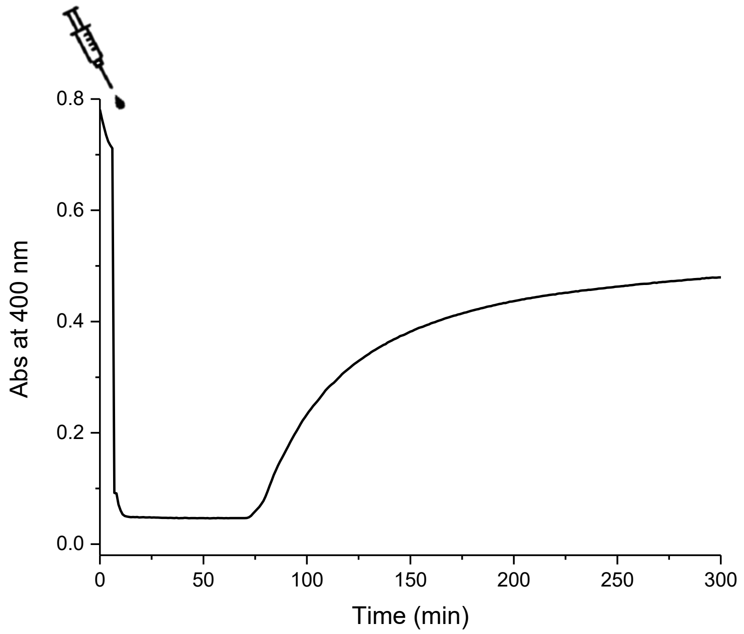

Figure S2.1 Plot of the absorbance at 400 nm versus time for a $5.0 \cdot 10^{-5}$ M *p*-nitrophenol solution in 80% DMSO, 10 mM K_2CO_3, upon an addition of 20.0 mM of trichloroacetic acid. This experiment provided the molar extinction coefficient for *p*-nitrophenol and *p*-nitrophenolate reported in the Experimental Section.

Section S3 Kinetic Experiments in the presence of the Catalysts

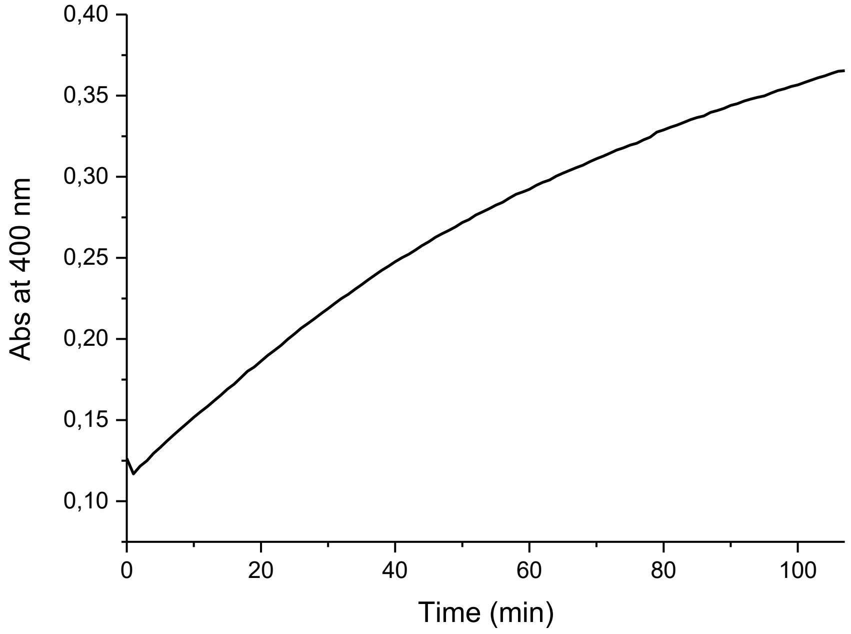

Figure S3.1 **1**-Cu^{II} 2.0 mM, HPNP 0.2 mM, TCA 10 mM, no prior pH adjustment.

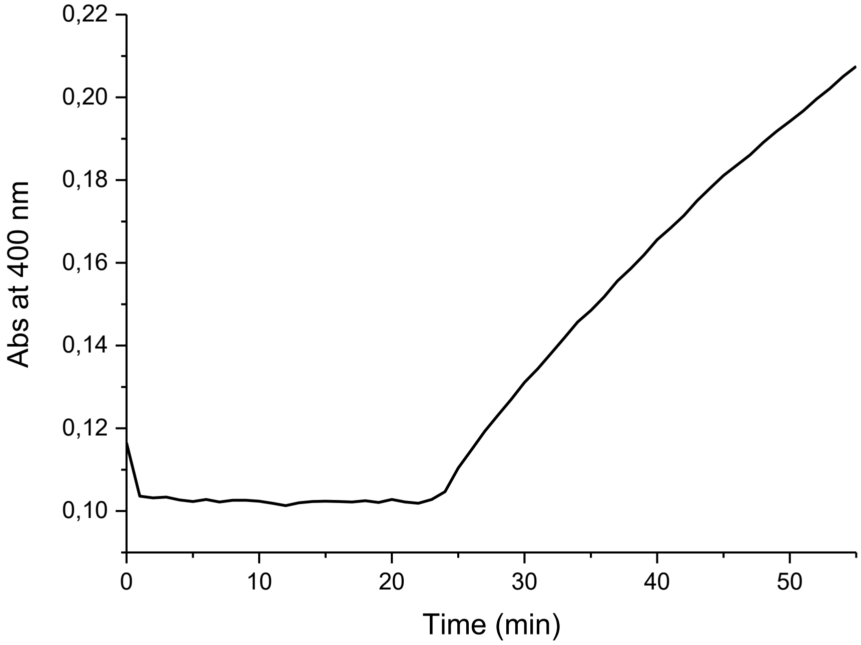

Figure S3.2 **1**-Cu^{II} 2.0 mM, HPNP 0.2 mM, TCA 15 mM, no prior pH adjustment.

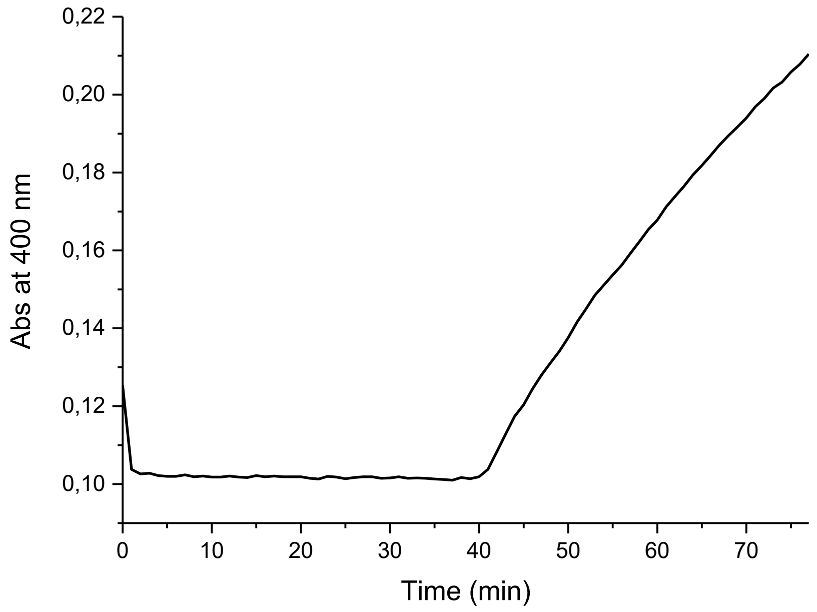

Figure S3.3 **1**-Cu^{II} 2.0 mM, HPNP 0.2 mM, TCA 20 mM, no prior pH adjustment.

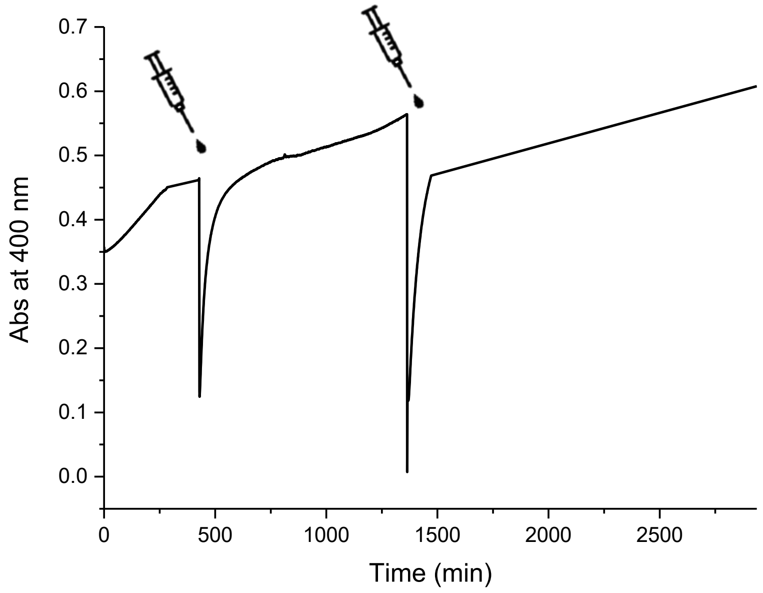

Figure S3.4 **1**-Cu^{II} 0.5 mM, HPNP 0.2 mM, TCA 10 mM; prior pH adjustment at 9.0.

This experiment, in comparison with that reported in Figure S3.5, demonstrates the influence of the catalyst concentration on the duration of the stationary state: the higher the catalyst concentration, the shorter the stationary state duration. It should be emphasized that both the occurrence and the persistence of the stationary state are highly dependent on the experimental conditions.

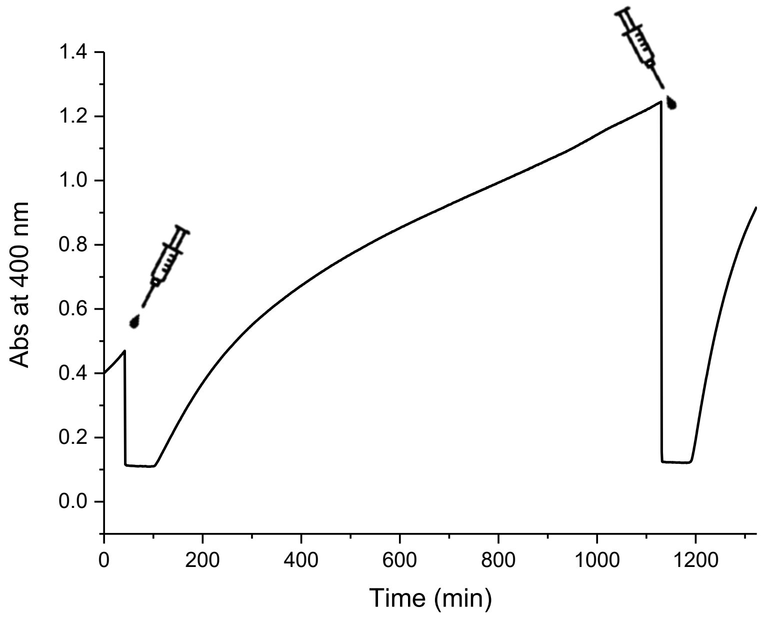

Figure S3.5 **1**-Cu^{II} 2.0 mM, HPNP 0.2 mM, TCA 10 mM; prior pH adjustment at 9.3.

This experiment, in comparison with that reported in Figure S3.4 demonstrates the influence of the catalyst concentration on the duration of the stationary state.

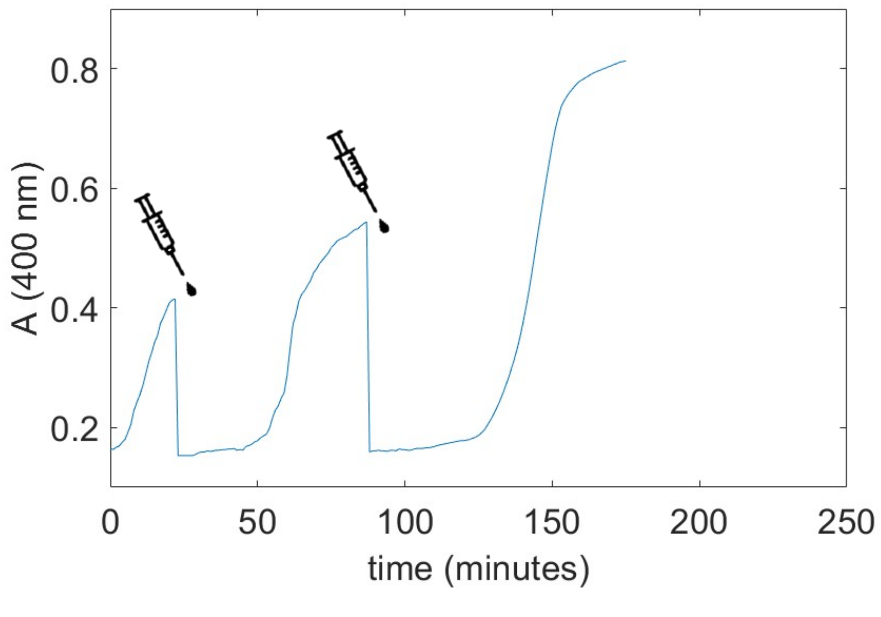

Figure S3.6 **2**-Cu^{II} 1.0 mM, HPNP 0.05 mM, TCA 10 mM, K_2CO_3 10 mM; no prior pH adjustment. The substrate was added as last component.

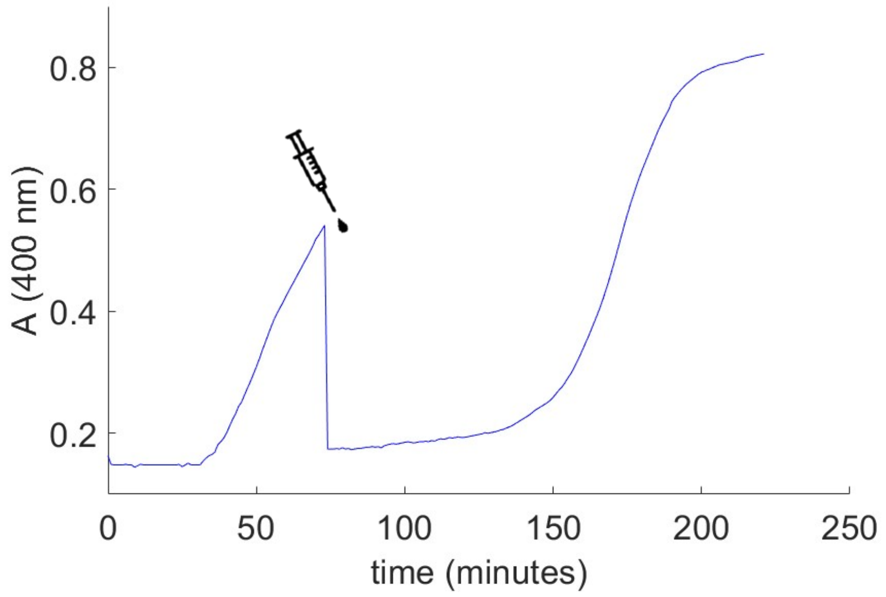

Figure S3.7 **2**-Cu^{II} 1.0 mM, HPNP 0.05 mM, TCA 20 mM, K_2CO_3 10 mM; no prior pH adjustment. The substrate was added as last component. Compare with Figure S3.6.

Section S4 pH meter calibration in 80% DMSO

The calibration of the electrode was carried out through a comparison with buffer solution of known pK_a values (see reference 58 in the main text), assumed to be the corrected pH values. The buffer solutions were mixtures of five different 10.0 mM commercial buffer acid components (see table below) and commercial 5.0 mM Tetramethylammonium hydroxide (NMe_4OH) as buffer basic component. The time elapsed to ensure a stable pH reading ranges from 5 to 10 minutes. Every measurement was repeated three times, and the obtained mean values are here reported.

Table S4.1

pH_{corr}	pH_{read}	Buffer acid component
13.83	14.55	Mesitol
11.98	12.64	*p*-chlorophenol
9.83	10.34	*p*-cianophenol
8.00	9.07	Acetic acid
3.87	4.16	Dichloroacetic acid

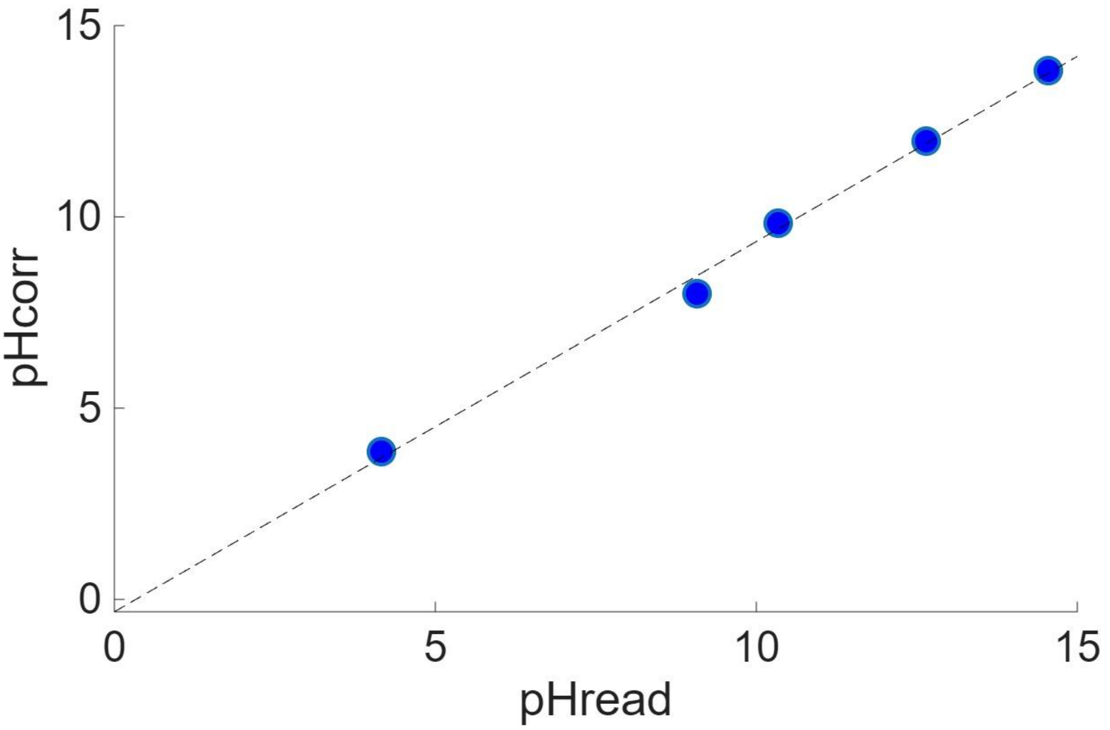

Figure S4.1 pH meter calibration curve obtained using different buffer solutions with known pK_a. See the Table reported above

Section S5 Raw Potentiometric and UV-Vis Data

TCA 10 mM, K_2CO_3 10 mM

time (min)	pH_{corr}
0	12.3700000000000
1	3.29000000000000
6	3.39000000000000
11	3.48000000000000
16	3.58000000000000
21	3.71000000000000
27	3.93000000000000
32	4.28000000000000
33	4.41000000000000
34	4.55000000000000
35	4.77000000000000

36	5.18000000000000
37	6.25000000000000
38	7.61000000000000
39	7.96000000000000
41	8.25000000000000
44	8.47000000000000
51	8.76000000000000
62	8.99000000000000
66	9.04000000000000
67	3.17000000000000
76	3.22000000000000
84	3.30000000000000
97	3.48000000000000
107	3.69000000000000
113	4.13000000000000
116	4.31000000000000
118	4.44000000000000
119	4.80000000000000
121	5.16000000000000
122	5.94000000000000
123	7.24000000000000
124	7.66000000000000
125	7.85000000000000
126	8.09000000000000
128	8.47000000000000
135	8.84000000000000
152	8.87000000000000
155	3.15000000000000
156	3.21000000000000
163	3.26000000000000
167	3.41000000000000
179	3.63000000000000
190	3.92000000000000
198	4.26000000000000

203	4.48000000000000
205	5.22000000000000
208	6.02000000000000
209	7.18000000000000
210	7.54000000000000
211	7.88000000000000
213	8.06000000000000
215	8.33000000000000
220	8.66000000000000
236	8.75000000000000
243	8.85000000000000
254	8.90000000000000

1-Cu^{II} 1.0 mM, K_2CO_3 10 mM, HPNP 0.20 mM, TCA 10 mM

t(min)	A_{400nm} (a.u.)
0	0.144300000000000
1	0.138900000000000
2	0.138200000000000
3	0.138000000000000
4	0.138000000000000
5	0.138400000000000
6	0.138100000000000
7	0.136600000000000
8	0.135900000000000
9	0.135800000000000
10	0.135900000000000
11	0.136000000000000
12	0.135800000000000
13	0.136000000000000
14	0.135900000000000
15	0.136000000000000
16	0.136100000000000

17	0.136700000000000
18	0.136500000000000
19	0.136500000000000
20	0.135400000000000
21	0.134900000000000
22	0.134600000000000
23	0.134500000000000
24	0.134400000000000
25	0.134400000000000
26	0.133800000000000
27	0.134100000000000
28	0.133800000000000
29	0.134000000000000
30	0.134200000000000
31	0.134000000000000
32	0.134100000000000
33	0.134000000000000
34	0.134000000000000
35	0.134600000000000
36	0.134700000000000
37	0.134500000000000
38	0.134300000000000
39	0.134000000000000
40	0.134000000000000
41	0.133400000000000
42	0.133500000000000
43	0.133900000000000
44	0.133800000000000
45	0.133700000000000
46	0.133900000000000
47	0.133400000000000
48	0.133800000000000
49	0.133800000000000
50	0.134200000000000

51	0.133600000000000
52	0.133700000000000
53	0.134100000000000
54	0.142800000000000
55	0.140900000000000
56	0.141300000000000
57	0.141300000000000
58	0.141400000000000
59	0.140700000000000
60	0.140500000000000
61	0.140000000000000
62	0.139500000000000
63	0.139000000000000
64	0.139100000000000
65	0.138400000000000
66	0.138700000000000
67	0.138700000000000
68	0.138200000000000
69	0.137500000000000
70	0.137600000000000
71	0.137800000000000
72	0.137700000000000
73	0.137600000000000
74	0.137700000000000
75	0.137600000000000
76	0.138100000000000
77	0.137800000000000
78	0.137900000000000
79	0.138000000000000
80	0.137400000000000
81	0.137900000000000
82	0.137700000000000
83	0.137500000000000
84	0.138000000000000

85	0.137800000000000
86	0.138400000000000
87	0.138600000000000
88	0.139000000000000
89	0.138600000000000
90	0.138800000000000
91	0.138600000000000
92	0.138700000000000
93	0.139000000000000
94	0.138900000000000
95	0.139100000000000
96	0.139600000000000
97	0.139200000000000
98	0.139300000000000
99	0.139300000000000
100	0.139600000000000
101	0.140300000000000
102	0.140300000000000
103	0.140000000000000
104	0.140400000000000
105	0.140100000000000
106	0.140100000000000
107	0.139800000000000
108	0.139500000000000
109	0.139400000000000
110	0.139600000000000
111	0.139600000000000
112	0.139500000000000
113	0.139900000000000
114	0.140500000000000
115	0.140500000000000
116	0.140800000000000
117	0.140500000000000
118	0.140900000000000

119	0.141100000000000
120	0.141100000000000
121	0.141100000000000
122	0.141500000000000
123	0.141900000000000
124	0.142200000000000
125	0.142200000000000
126	0.141900000000000
127	0.142000000000000
128	0.142500000000000
129	0.142500000000000
130	0.142200000000000
131	0.142500000000000
132	0.142500000000000
133	0.142500000000000
134	0.142800000000000
135	0.142500000000000
136	0.142800000000000
137	0.143200000000000
138	0.143400000000000
139	0.143600000000000
140	0.143600000000000
141	0.143900000000000
142	0.144100000000000
143	0.143800000000000
144	0.144300000000000
145	0.143800000000000
146	0.144200000000000
147	0.144000000000000
148	0.144700000000000
150	0.105300000000000
151	0.101100000000000
152	0.0977000000000000
153	0.0974000000000000

154	0.0972000000000000
155	0.0978000000000000
156	0.0978000000000000
157	0.0966000000000000
158	0.0968000000000000
159	0.0963000000000000
160	0.0960000000000000
161	0.0963000000000000
162	0.0960000000000000
163	0.0958000000000000
164	0.0953000000000000
165	0.0954000000000000
166	0.0954000000000000
167	0.0952000000000000
168	0.0950000000000000
169	0.0951000000000000
170	0.0945000000000000
171	0.0945000000000000
172	0.0946000000000000
173	0.0947000000000000
174	0.0947000000000000
175	0.0945000000000000
176	0.0942000000000000
177	0.0939000000000000
178	0.0939000000000000
179	0.0935000000000000
180	0.0938000000000000
181	0.0935000000000000
182	0.0939000000000000
183	0.0940000000000000
184	0.0944000000000000
185	0.0945000000000000
186	0.0942000000000000
187	0.0941000000000000

188	0.0941000000000000
189	0.0937000000000000
190	0.0936000000000000
191	0.0935000000000000
192	0.0937000000000000
193	0.0939000000000000
194	0.0938000000000000
196	0.0981000000000000
197	0.0984000000000000
198	0.0975000000000000
199	0.0975000000000000
200	0.0976000000000000
201	0.0967000000000000
202	0.0959000000000000
203	0.0963000000000000
204	0.0963000000000000
205	0.0958000000000000
206	0.0958000000000000
207	0.0955000000000000
208	0.0964000000000000
209	0.0951000000000000
210	0.0951000000000000
211	0.0946000000000000
212	0.0945000000000000
213	0.0951000000000000
214	0.0947000000000000
215	0.0940000000000000
216	0.0941000000000000
217	0.0944000000000000
218	0.0948000000000000
219	0.0949000000000000
220	0.0946000000000000
221	0.0949000000000000
222	0.0946000000000000

223	0.0948000000000000
224	0.0948000000000000
225	0.0949000000000000
226	0.0951000000000000
227	0.0957000000000000
228	0.0956000000000000
229	0.0957000000000000
230	0.0955000000000000
231	0.0956000000000000
232	0.0960000000000000
233	0.0961000000000000
234	0.0964000000000000
235	0.0968000000000000
236	0.0974000000000000
237	0.0970000000000000
238	0.0972000000000000
239	0.0975000000000000
240	0.0981000000000000
241	0.0981000000000000
242	0.0986000000000000
243	0.0989000000000000
244	0.0991000000000000
245	0.0999000000000000
246	0.0991000000000000
247	0.0994000000000000
248	0.0998000000000000
249	0.0991000000000000
250	0.0993000000000000
251	0.0992000000000000
252	0.0997000000000000
253	0.0999000000000000
254	0.100300000000000
255	0.100600000000000
256	0.101200000000000

257	0.101300000000000
258	0.100700000000000
259	0.101000000000000
260	0.101400000000000
261	0.101700000000000
262	0.101300000000000
263	0.101700000000000
264	0.101600000000000
265	0.102100000000000
266	0.102200000000000
267	0.102400000000000
268	0.102500000000000
269	0.102800000000000
270	0.102800000000000
271	0.103100000000000
272	0.103200000000000
273	0.103000000000000
274	0.103700000000000
275	0.103500000000000
276	0.103800000000000
277	0.104100000000000
278	0.104000000000000
279	0.104100000000000
280	0.104700000000000
281	0.104800000000000
282	0.104900000000000
283	0.105100000000000
284	0.105300000000000
285	0.105300000000000
286	0.105200000000000
287	0.104900000000000
288	0.105600000000000
289	0.105600000000000
290	0.105500000000000

291	0.105800000000000
292	0.105400000000000
293	0.106000000000000
294	0.106300000000000
295	0.105900000000000
296	0.106600000000000
297	0.107100000000000
298	0.107200000000000
299	0.107400000000000
300	0.107800000000000
301	0.107500000000000
302	0.107800000000000
303	0.107600000000000
304	0.108200000000000
305	0.107800000000000
306	0.108000000000000
307	0.108100000000000
308	0.108000000000000
309	0.108200000000000
310	0.108200000000000
311	0.108400000000000
312	0.108600000000000
313	0.109100000000000
314	0.109700000000000
315	0.110000000000000
316	0.110600000000000
317	0.110500000000000
318	0.110700000000000
319	0.110400000000000
320	0.110700000000000
321	0.110800000000000
322	0.110900000000000
323	0.110900000000000
324	0.111100000000000

325	0.111000000000000
326	0.111500000000000
327	0.111500000000000
328	0.111200000000000
329	0.111600000000000
330	0.111500000000000
331	0.112000000000000
332	0.111900000000000
333	0.112300000000000
334	0.112200000000000
335	0.112800000000000
336	0.113200000000000
337	0.113300000000000
338	0.113300000000000
339	0.113200000000000
340	0.113300000000000
341	0.113900000000000
342	0.114300000000000
343	0.114300000000000
344	0.114100000000000
345	0.114300000000000
346	0.114700000000000
347	0.114900000000000
348	0.115100000000000
349	0.115200000000000
350	0.115100000000000
351	0.115400000000000
352	0.115600000000000
353	0.116100000000000
354	0.116000000000000
355	0.116100000000000
356	0.115800000000000
357	0.116000000000000
358	0.116100000000000

359	0.116100000000000
360	0.116600000000000
361	0.116700000000000
362	0.116900000000000
363	0.117300000000000
364	0.117300000000000
365	0.117400000000000
366	0.117600000000000
367	0.117600000000000
368	0.117900000000000
369	0.117900000000000
370	0.118000000000000
371	0.118400000000000
372	0.118600000000000
373	0.118700000000000
374	0.119000000000000
375	0.119500000000000
376	0.119400000000000
377	0.119800000000000
378	0.119800000000000
379	0.119600000000000
380	0.119900000000000
381	0.119500000000000
382	0.120000000000000
383	0.120000000000000
384	0.120600000000000
385	0.120600000000000
386	0.120600000000000
387	0.121000000000000
388	0.120900000000000
389	0.121700000000000
390	0.122100000000000
391	0.122100000000000
392	0.122000000000000

393	0.122200000000000
394	0.121900000000000
395	0.122200000000000
396	0.122500000000000
397	0.122500000000000
398	0.123200000000000
399	0.123500000000000
400	0.123200000000000
401	0.123700000000000
402	0.124000000000000
403	0.123800000000000
404	0.124600000000000
405	0.124400000000000
406	0.125000000000000
407	0.125000000000000
408	0.125300000000000
409	0.125000000000000
410	0.125300000000000
411	0.125100000000000
412	0.125300000000000
413	0.125200000000000
414	0.125900000000000
415	0.125700000000000
416	0.126100000000000
417	0.126200000000000
418	0.126300000000000
419	0.126100000000000
420	0.126200000000000
421	0.126900000000000
422	0.127200000000000
423	0.127300000000000
424	0.127500000000000
425	0.127700000000000
426	0.128100000000000

427	0.128300000000000
428	0.128600000000000
429	0.128300000000000
430	0.128000000000000
431	0.128200000000000
432	0.128400000000000
433	0.128700000000000
434	0.128900000000000
435	0.128900000000000
436	0.128900000000000
437	0.129300000000000
438	0.129400000000000
439	0.129600000000000
440	0.129700000000000
441	0.129900000000000
442	0.130100000000000
443	0.130000000000000
444	0.130200000000000
445	0.130200000000000
446	0.130500000000000
447	0.130500000000000
448	0.130700000000000
449	0.130900000000000
450	0.130900000000000
451	0.131100000000000
452	0.131200000000000
453	0.131000000000000
454	0.131500000000000
455	0.131300000000000
456	0.131500000000000
457	0.131600000000000
458	0.132300000000000
459	0.132300000000000
460	0.132700000000000

461	0.132600000000000
462	0.132800000000000
463	0.132800000000000
464	0.132900000000000
465	0.133200000000000
466	0.133500000000000
467	0.133800000000000
468	0.133700000000000
469	0.133600000000000
470	0.133200000000000
471	0.133600000000000
472	0.133800000000000
473	0.134400000000000
474	0.134300000000000
475	0.134600000000000
476	0.134800000000000
477	0.134500000000000
478	0.135200000000000
479	0.135600000000000
480	0.135800000000000
481	0.135700000000000
482	0.135900000000000
483	0.136100000000000
484	0.136200000000000
485	0.136100000000000
486	0.136600000000000
487	0.136200000000000
488	0.136200000000000
489	0.136400000000000
490	0.136700000000000
491	0.136600000000000
492	0.137300000000000
493	0.137500000000000
494	0.137500000000000

495	0.137800000000000
496	0.138000000000000
497	0.138400000000000
498	0.138500000000000
499	0.138400000000000
500	0.138900000000000
501	0.138700000000000
502	0.139300000000000
503	0.139200000000000
504	0.139300000000000
505	0.139100000000000
506	0.139000000000000
507	0.138700000000000
508	0.138900000000000
509	0.139000000000000
510	0.139500000000000
511	0.139900000000000
512	0.139800000000000
513	0.140100000000000
514	0.140600000000000
515	0.140500000000000
516	0.140700000000000
517	0.140800000000000
518	0.141400000000000
519	0.141400000000000
520	0.141300000000000
521	0.141700000000000
522	0.141400000000000
523	0.141600000000000
524	0.141700000000000
525	0.141600000000000
526	0.141700000000000
527	0.141800000000000
528	0.141900000000000

529	0.142200000000000
530	0.142300000000000
531	0.142600000000000
532	0.142700000000000
533	0.142700000000000
534	0.142800000000000
535	0.143100000000000
536	0.143700000000000
537	0.143400000000000
538	0.143600000000000
539	0.143900000000000
540	0.143600000000000
541	0.143700000000000
542	0.144200000000000
543	0.143800000000000
544	0.143700000000000
545	0.143800000000000
546	0.144500000000000
547	0.144400000000000
548	0.144500000000000
549	0.144800000000000
550	0.144700000000000
551	0.144900000000000
552	0.145000000000000
553	0.145300000000000
554	0.145500000000000
555	0.145600000000000
556	0.145400000000000
557	0.145600000000000
558	0.146000000000000
559	0.146100000000000
560	0.146600000000000
561	0.146200000000000
562	0.146100000000000

563	0.146400000000000
564	0.146700000000000
565	0.146100000000000
566	0.146400000000000
567	0.146800000000000
568	0.146700000000000
569	0.147200000000000
570	0.147100000000000
571	0.147400000000000
572	0.147400000000000
573	0.147800000000000
574	0.147800000000000
575	0.147400000000000
576	0.148100000000000
577	0.148100000000000
578	0.147800000000000
579	0.148100000000000
580	0.148600000000000
581	0.148900000000000
582	0.148700000000000
583	0.149200000000000
584	0.148800000000000
585	0.149000000000000
586	0.149300000000000
587	0.149100000000000
588	0.149700000000000
589	0.149700000000000
590	0.149500000000000
591	0.149700000000000
592	0.150000000000000
593	0.150100000000000
594	0.150100000000000
595	0.150400000000000
596	0.149800000000000

597	0.150000000000000
598	0.150900000000000
599	0.150900000000000
600	0.151500000000000
601	0.151900000000000
602	0.151600000000000
603	0.151800000000000
604	0.151800000000000
605	0.151400000000000
606	0.151600000000000
607	0.151900000000000
608	0.152400000000000
609	0.151600000000000
610	0.151900000000000
611	0.151900000000000
612	0.151900000000000
613	0.152200000000000
614	0.152100000000000
615	0.152500000000000
616	0.152500000000000
617	0.152700000000000
618	0.152500000000000
619	0.152400000000000
620	0.152900000000000
621	0.153200000000000
622	0.153700000000000
623	0.153300000000000
624	0.153300000000000
625	0.153000000000000
626	0.153500000000000
627	0.153800000000000
628	0.153900000000000
629	0.154100000000000
630	0.154100000000000

631	0.154200000000000
632	0.154000000000000
633	0.153800000000000
634	0.154700000000000
635	0.154400000000000
636	0.154900000000000
637	0.154500000000000
638	0.154600000000000
639	0.155100000000000
640	0.155100000000000
641	0.155800000000000
642	0.155400000000000
643	0.155200000000000
644	0.155600000000000
645	0.155500000000000
646	0.155700000000000
647	0.155800000000000
648	0.156300000000000
649	0.156100000000000
650	0.156000000000000
651	0.156000000000000
652	0.156000000000000
653	0.156300000000000
654	0.156400000000000
655	0.156600000000000
656	0.156700000000000
657	0.157000000000000
658	0.157200000000000
659	0.157100000000000
660	0.156900000000000
661	0.157300000000000
662	0.157200000000000
663	0.157600000000000
664	0.157500000000000

665	0.157500000000000
666	0.157300000000000
667	0.157500000000000
668	0.157500000000000
669	0.157700000000000
670	0.158500000000000
671	0.158000000000000
672	0.158600000000000
673	0.158800000000000
674	0.158400000000000
675	0.158500000000000
676	0.158800000000000
677	0.158700000000000
678	0.158800000000000
679	0.159000000000000
680	0.159200000000000
681	0.159100000000000
682	0.159200000000000
683	0.159500000000000
684	0.159700000000000
685	0.159900000000000
686	0.160000000000000
687	0.159400000000000
688	0.159900000000000
689	0.159900000000000
690	0.159900000000000
691	0.160100000000000
692	0.160300000000000
693	0.160700000000000
694	0.160700000000000
695	0.160500000000000
696	0.160600000000000
697	0.161100000000000
698	0.160900000000000

699	0.160400000000000
700	0.161200000000000
701	0.161400000000000
702	0.161200000000000
703	0.160900000000000
704	0.161300000000000
705	0.161700000000000
706	0.161800000000000
707	0.161900000000000
708	0.161900000000000
709	0.161800000000000
710	0.162000000000000
711	0.161500000000000
712	0.161800000000000
713	0.161700000000000
714	0.162100000000000
715	0.162200000000000
716	0.162200000000000
717	0.162500000000000
718	0.162700000000000
719	0.162800000000000
720	0.162600000000000
721	0.162800000000000
722	0.162800000000000
723	0.162600000000000
724	0.163200000000000
725	0.163400000000000
726	0.163000000000000
727	0.163600000000000
728	0.163100000000000
729	0.163600000000000
730	0.163700000000000
731	0.163300000000000
732	0.163400000000000

733	0.163800000000000
734	0.164000000000000
735	0.163900000000000
736	0.164400000000000
737	0.164600000000000
738	0.164700000000000
739	0.164500000000000
740	0.165000000000000
741	0.164700000000000
742	0.164400000000000
743	0.164400000000000
744	0.164200000000000
745	0.164800000000000
746	0.164900000000000
747	0.165000000000000
748	0.164900000000000
749	0.165100000000000
750	0.165300000000000
751	0.165300000000000
752	0.165600000000000
753	0.166500000000000
754	0.165900000000000
755	0.165800000000000
756	0.165900000000000
757	0.166000000000000
758	0.166100000000000
759	0.166000000000000
760	0.165800000000000
761	0.166500000000000
762	0.166600000000000
763	0.166200000000000
764	0.166100000000000
765	0.166700000000000
766	0.166800000000000

767	0.166600000000000
768	0.166800000000000
769	0.166900000000000
770	0.166900000000000
771	0.166800000000000
772	0.166600000000000
773	0.167200000000000
774	0.167400000000000
775	0.167200000000000
776	0.168100000000000
777	0.168000000000000
778	0.167900000000000
779	0.167800000000000
780	0.167500000000000
781	0.167700000000000
782	0.168000000000000
783	0.167900000000000
784	0.168000000000000
785	0.168200000000000
786	0.168300000000000
787	0.168200000000000
788	0.168500000000000
789	0.168300000000000
790	0.168500000000000
791	0.168600000000000
792	0.168600000000000
793	0.168500000000000
794	0.168700000000000
795	0.168600000000000
796	0.168800000000000
797	0.168800000000000
798	0.169100000000000
799	0.169300000000000
800	0.169200000000000

801	0.169300000000000
802	0.169300000000000
803	0.169300000000000
804	0.169300000000000
805	0.169300000000000
806	0.169800000000000
807	0.169800000000000
808	0.169800000000000
809	0.169700000000000
810	0.170200000000000
811	0.170200000000000
812	0.170400000000000
813	0.170400000000000
814	0.170600000000000
815	0.170300000000000
816	0.170200000000000
817	0.170500000000000
818	0.170200000000000
819	0.170300000000000
820	0.170700000000000
821	0.170900000000000
822	0.171200000000000
823	0.171000000000000
824	0.170900000000000
825	0.171000000000000
826	0.171200000000000
827	0.171100000000000
828	0.171100000000000
829	0.171300000000000
830	0.171500000000000
831	0.171700000000000
832	0.171700000000000
833	0.171600000000000
834	0.171800000000000

835	0.171600000000000
836	0.172000000000000
837	0.171900000000000
838	0.171800000000000
839	0.172100000000000
840	0.172300000000000
841	0.172400000000000
842	0.172600000000000
843	0.172000000000000
844	0.172800000000000
845	0.172500000000000
846	0.172800000000000
847	0.173200000000000
848	0.173000000000000
849	0.172900000000000
850	0.173000000000000
851	0.173200000000000
852	0.173100000000000
853	0.173100000000000
854	0.173400000000000
855	0.173400000000000
856	0.172900000000000
857	0.173100000000000
858	0.173100000000000
859	0.173600000000000
860	0.173700000000000
861	0.173600000000000
862	0.173700000000000
863	0.174000000000000
864	0.174100000000000
865	0.174000000000000
866	0.174100000000000
867	0.174300000000000
868	0.174100000000000

869	0.174300000000000
870	0.174600000000000
871	0.174700000000000
872	0.174700000000000
873	0.174800000000000
874	0.175000000000000
875	0.175400000000000
876	0.175200000000000
877	0.175300000000000
878	0.175500000000000
879	0.175300000000000
880	0.175400000000000
881	0.175000000000000
882	0.175200000000000
883	0.175500000000000
884	0.175200000000000
885	0.175500000000000
886	0.175400000000000
887	0.175300000000000
888	0.175700000000000
889	0.175800000000000
890	0.175800000000000
891	0.175900000000000
892	0.176100000000000
893	0.176300000000000
894	0.176200000000000
895	0.176600000000000
896	0.176500000000000
897	0.176700000000000
898	0.176600000000000
899	0.176800000000000
900	0.176800000000000
901	0.177000000000000
902	0.177300000000000

903	0.177100000000000
904	0.177200000000000
905	0.177200000000000
906	0.177100000000000
907	0.177300000000000
908	0.177100000000000
909	0.177200000000000
910	0.177100000000000
911	0.177300000000000
912	0.177400000000000
913	0.177600000000000
914	0.177800000000000
915	0.177600000000000
916	0.177200000000000
917	0.177700000000000
918	0.177900000000000
919	0.177800000000000
920	0.178100000000000
921	0.178000000000000
922	0.178100000000000
923	0.177900000000000
924	0.178600000000000
925	0.178600000000000
926	0.178600000000000
927	0.178700000000000
928	0.179000000000000
929	0.178800000000000
930	0.179000000000000
931	0.178700000000000
932	0.178600000000000
933	0.178700000000000
934	0.179000000000000
935	0.178700000000000
936	0.179200000000000

937	0.178900000000000
938	0.179400000000000
939	0.179600000000000
940	0.179400000000000
941	0.179500000000000
942	0.179600000000000
943	0.179800000000000
944	0.179700000000000
945	0.179700000000000
946	0.179500000000000
947	0.179500000000000
948	0.180000000000000
949	0.180000000000000
950	0.180200000000000
951	0.180000000000000
952	0.180100000000000
953	0.180500000000000
954	0.180400000000000
955	0.180600000000000
956	0.180400000000000
957	0.180600000000000
958	0.180500000000000
959	0.180300000000000
960	0.180500000000000
961	0.180200000000000
962	0.181100000000000
963	0.181000000000000
964	0.180800000000000
965	0.181000000000000
966	0.180600000000000
967	0.181000000000000
968	0.181200000000000
969	0.181200000000000
970	0.181000000000000

971	0.181300000000000
972	0.181200000000000
973	0.181500000000000
974	0.181400000000000
975	0.181400000000000
976	0.181500000000000
977	0.181700000000000
978	0.181600000000000
979	0.181400000000000
980	0.181400000000000
981	0.181600000000000
982	0.181700000000000
983	0.182200000000000
984	0.181900000000000
985	0.182000000000000
986	0.182300000000000
987	0.182200000000000
988	0.182200000000000
989	0.182200000000000
990	0.182600000000000
991	0.182500000000000
992	0.182800000000000
993	0.182800000000000
994	0.182600000000000
995	0.182800000000000
996	0.182700000000000
997	0.182800000000000
998	0.182800000000000
999	0.182700000000000
1000	0.182600000000000
1001	0.182900000000000
1002	0.183000000000000
1003	0.183000000000000
1004	0.183200000000000

1005	0.183300000000000
1006	0.183300000000000
1007	0.183400000000000
1008	0.183000000000000
1009	0.183900000000000
1010	0.183400000000000
1011	0.183800000000000
1012	0.183400000000000
1013	0.183700000000000
1014	0.183800000000000
1015	0.183900000000000
1016	0.183900000000000
1017	0.184100000000000
1018	0.184200000000000
1019	0.184000000000000
1020	0.184300000000000
1021	0.184300000000000
1022	0.184100000000000
1023	0.184500000000000
1024	0.184400000000000
1025	0.184600000000000
1026	0.184800000000000
1027	0.184700000000000
1028	0.184500000000000
1029	0.184900000000000
1030	0.184800000000000
1031	0.185100000000000
1032	0.185000000000000
1033	0.184800000000000
1034	0.184800000000000
1035	0.185100000000000
1036	0.184700000000000
1037	0.184800000000000
1038	0.185000000000000

1039	0.185600000000000
1040	0.185300000000000
1041	0.185300000000000
1042	0.185600000000000
1043	0.185600000000000
1044	0.185900000000000
1045	0.185700000000000
1046	0.185800000000000
1047	0.185700000000000
1048	0.186100000000000
1049	0.185800000000000
1050	0.185900000000000
1051	0.185800000000000
1052	0.186000000000000
1053	0.186100000000000
1054	0.185800000000000
1055	0.185800000000000
1056	0.186300000000000
1057	0.186100000000000
1058	0.186400000000000
1059	0.186300000000000
1060	0.186200000000000
1061	0.186500000000000
1062	0.186400000000000
1063	0.186600000000000
1064	0.186500000000000
1065	0.186600000000000
1066	0.186900000000000
1067	0.187000000000000
1068	0.186700000000000
1069	0.186900000000000
1070	0.186900000000000
1071	0.187000000000000
1072	0.187100000000000

1073	0.186800000000000
1074	0.187000000000000
1075	0.187200000000000
1076	0.187500000000000
1077	0.187300000000000
1078	0.187300000000000
1079	0.187400000000000
1080	0.187500000000000
1081	0.187400000000000
1082	0.187600000000000
1083	0.187500000000000
1084	0.187700000000000
1085	0.187600000000000
1086	0.187900000000000
1087	0.187900000000000
1088	0.187900000000000
1089	0.188100000000000
1090	0.188100000000000
1091	0.188200000000000
1092	0.188300000000000
1093	0.188200000000000
1094	0.188200000000000
1095	0.188100000000000
1096	0.188000000000000
1097	0.188200000000000
1098	0.188600000000000
1099	0.188300000000000
1100	0.188600000000000
1101	0.188900000000000
1102	0.188600000000000
1103	0.188600000000000
1104	0.188700000000000
1105	0.188900000000000
1106	0.188600000000000

1107	0.189200000000000
1108	0.188900000000000
1109	0.189200000000000
1110	0.189200000000000
1111	0.189100000000000
1112	0.189000000000000
1113	0.189300000000000
1114	0.189400000000000
1115	0.189100000000000
1116	0.189300000000000
1117	0.189400000000000
1118	0.189200000000000
1119	0.189300000000000
1120	0.189700000000000
1121	0.190000000000000
1122	0.190000000000000
1123	0.189600000000000
1124	0.189700000000000
1125	0.190000000000000
1126	0.189900000000000
1127	0.190000000000000
1128	0.190300000000000
1129	0.189900000000000
1130	0.189700000000000
1131	0.190100000000000
1132	0.190200000000000
1133	0.190200000000000
1134	0.190300000000000
1135	0.190000000000000
1136	0.190400000000000
1137	0.190400000000000
1138	0.190300000000000
1139	0.190400000000000
1140	0.190400000000000

1141	0.190800000000000
1142	0.190500000000000
1143	0.190900000000000
1144	0.190700000000000
1145	0.190900000000000
1146	0.191400000000000
1147	0.190800000000000
1148	0.190800000000000
1149	0.191000000000000
1150	0.191000000000000
1151	0.190900000000000
1152	0.191400000000000
1153	0.190800000000000
1154	0.191100000000000
1155	0.190900000000000
1156	0.191000000000000
1157	0.191500000000000
1158	0.191100000000000
1159	0.190900000000000
1160	0.191000000000000
1161	0.191400000000000
1162	0.191500000000000
1163	0.191400000000000
1164	0.191500000000000
1165	0.191700000000000
1166	0.191400000000000
1167	0.192000000000000
1168	0.192100000000000
1169	0.191800000000000
1170	0.192200000000000
1171	0.191800000000000
1172	0.192400000000000
1173	0.192400000000000
1174	0.192300000000000

1175	0.192100000000000
1176	0.192200000000000
1177	0.192200000000000
1178	0.192300000000000
1179	0.192100000000000
1180	0.192400000000000
1181	0.192400000000000
1182	0.192700000000000
1183	0.192300000000000
1184	0.192500000000000
1185	0.192600000000000
1186	0.192800000000000
1187	0.192600000000000
1188	0.193000000000000
1189	0.193200000000000
1190	0.193000000000000
1191	0.192900000000000
1192	0.192900000000000
1193	0.193400000000000
1194	0.193000000000000
1195	0.193000000000000
1196	0.193400000000000
1197	0.193100000000000
1198	0.193400000000000
1199	0.193300000000000
1200	0.193300000000000
1201	0.193500000000000
1202	0.193700000000000
1203	0.193500000000000
1204	0.193600000000000
1205	0.193800000000000
1206	0.193400000000000
1207	0.193800000000000
1208	0.193400000000000

1209	0.193900000000000
1210	0.194200000000000
1211	0.194000000000000
1212	0.194000000000000
1213	0.193800000000000
1214	0.194100000000000
1215	0.194000000000000
1216	0.194200000000000
1217	0.194000000000000
1218	0.194100000000000
1219	0.194300000000000
1220	0.194400000000000
1221	0.194600000000000
1222	0.194200000000000
1223	0.194300000000000
1224	0.194800000000000
1225	0.194600000000000
1226	0.194700000000000
1227	0.194200000000000
1228	0.194300000000000
1229	0.194800000000000
1230	0.195300000000000
1231	0.195000000000000
1232	0.195300000000000
1233	0.195200000000000
1234	0.195300000000000
1235	0.195100000000000
1236	0.195300000000000
1237	0.195300000000000
1238	0.195200000000000
1239	0.195100000000000
1240	0.195400000000000
1241	0.195500000000000
1242	0.195000000000000

1243	0.195400000000000
1244	0.195300000000000
1245	0.195400000000000
1246	0.195700000000000
1247	0.196000000000000
1248	0.195900000000000
1249	0.195800000000000
1250	0.195500000000000
1251	0.195900000000000
1252	0.195800000000000
1253	0.195900000000000
1254	0.196000000000000
1255	0.196100000000000
1256	0.196200000000000
1257	0.196100000000000
1258	0.196600000000000
1259	0.196700000000000
1260	0.196600000000000
1261	0.196800000000000
1262	0.196500000000000
1263	0.196800000000000
1264	0.197300000000000
1265	0.197100000000000
1266	0.197000000000000
1267	0.197300000000000
1268	0.197200000000000
1269	0.197200000000000
1270	0.197300000000000
1271	0.197300000000000
1272	0.197400000000000
1273	0.197800000000000
1274	0.197400000000000
1275	0.197500000000000
1276	0.198000000000000

1277	0.197900000000000
1278	0.197800000000000
1279	0.198600000000000
1280	0.198100000000000
1281	0.197700000000000
1282	0.197800000000000
1283	0.198200000000000
1284	0.198000000000000
1285	0.198200000000000
1286	0.198500000000000
1287	0.198600000000000
1288	0.198400000000000
1289	0.198200000000000
1290	0.198300000000000
1291	0.198800000000000
1292	0.198600000000000
1293	0.198400000000000
1294	0.198900000000000
1295	0.198900000000000
1296	0.199200000000000
1297	0.199500000000000
1298	0.199200000000000
1299	0.199900000000000
1300	0.199700000000000
1301	0.199400000000000
1302	0.199800000000000
1303	0.199400000000000
1304	0.199400000000000
1305	0.199400000000000
1306	0.199700000000000
1307	0.199800000000000
1308	0.199800000000000
1309	0.199700000000000
1310	0.199700000000000

1311	0.199600000000000
1312	0.199900000000000
1313	0.200200000000000
1314	0.199800000000000
1315	0.200200000000000
1316	0.200300000000000
1317	0.199900000000000
1318	0.200400000000000
1319	0.200300000000000
1320	0.200600000000000
1321	0.200700000000000
1322	0.200600000000000
1323	0.200800000000000
1324	0.200900000000000
1325	0.200900000000000
1326	0.201100000000000
1327	0.200600000000000
1328	0.201300000000000
1329	0.200900000000000
1330	0.200900000000000
1331	0.201000000000000
1332	0.201000000000000
1333	0.201100000000000
1334	0.201300000000000
1335	0.201200000000000
1336	0.201200000000000
1337	0.201600000000000
1338	0.201400000000000
1339	0.201700000000000
1340	0.202300000000000
1341	0.201900000000000
1342	0.202000000000000
1343	0.202400000000000
1344	0.202300000000000

1345	0.202400000000000
1346	0.202500000000000
1347	0.202100000000000
1348	0.202300000000000
1349	0.202600000000000
1350	0.202500000000000
1351	0.202900000000000
1352	0.202400000000000
1353	0.202500000000000
1354	0.202400000000000
1355	0.203000000000000
1356	0.202800000000000
1357	0.202700000000000
1358	0.203000000000000
1359	0.203400000000000
1360	0.203000000000000
1361	0.203000000000000
1362	0.203400000000000
1363	0.203300000000000
1364	0.203100000000000
1365	0.203400000000000
1366	0.203400000000000
1367	0.203400000000000
1368	0.203600000000000
1369	0.203600000000000
1370	0.203800000000000
1371	0.203800000000000
1372	0.203800000000000
1373	0.203800000000000
1374	0.203700000000000
1375	0.204000000000000
1376	0.204100000000000
1377	0.203900000000000
1378	0.204000000000000

1379	0.204200000000000
1380	0.204300000000000
1381	0.204300000000000
1382	0.204700000000000
1383	0.204300000000000
1384	0.204800000000000
1385	0.204800000000000
1386	0.204500000000000
1387	0.204800000000000
1388	0.204800000000000
1389	0.204800000000000
1390	0.204600000000000
1391	0.204700000000000
1392	0.204800000000000
1393	0.204900000000000
1394	0.205200000000000
1395	0.205000000000000
1396	0.205200000000000
1397	0.205200000000000
1398	0.204900000000000
1399	0.205400000000000
1400	0.205700000000000
1401	0.205400000000000
1402	0.205300000000000
1403	0.205400000000000
1404	0.205700000000000
1405	0.205800000000000
1406	0.205700000000000
1407	0.205800000000000
1408	0.206000000000000
1409	0.206100000000000
1410	0.206100000000000
1411	0.206100000000000
1412	0.206000000000000

1413	0.205800000000000
1414	0.206000000000000
1415	0.206200000000000
1416	0.206400000000000
1417	0.206500000000000
1418	0.206200000000000
1419	0.206500000000000
1421	0.129000000000000
1422	0.126100000000000
1423	0.125000000000000
1424	0.124900000000000
1425	0.124100000000000
1426	0.123600000000000
1427	0.123900000000000
1428	0.124600000000000
1429	0.124300000000000
1430	0.124000000000000
1431	0.124200000000000
1432	0.123900000000000
1433	0.123700000000000
1434	0.123400000000000
1435	0.123000000000000
1436	0.122400000000000
1437	0.122500000000000
1438	0.122400000000000
1439	0.122300000000000
1440	0.121600000000000
1441	0.121800000000000
1442	0.121800000000000
1443	0.122200000000000
1444	0.121800000000000
1445	0.121900000000000
1446	0.121600000000000
1447	0.121500000000000

1448	0.121900000000000
1449	0.121900000000000
1450	0.122000000000000
1451	0.121900000000000
1452	0.122200000000000
1453	0.121700000000000
1454	0.121400000000000
1455	0.121500000000000
1456	0.121400000000000
1457	0.121700000000000
1458	0.121200000000000
1459	0.121300000000000
1460	0.121600000000000
1461	0.121500000000000
1462	0.121100000000000
1463	0.121300000000000
1464	0.121200000000000
1465	0.121200000000000
1466	0.121500000000000
1467	0.122400000000000
1468	0.122400000000000
1469	0.122600000000000
1470	0.122500000000000
1471	0.122100000000000
1472	0.122100000000000
1473	0.122000000000000
1474	0.121400000000000
1475	0.120900000000000
1476	0.120900000000000
1477	0.121100000000000
1478	0.121400000000000
1479	0.121600000000000
1480	0.121800000000000
1481	0.121700000000000

1482	0.121600000000000
1483	0.121600000000000
1484	0.121700000000000
1485	0.121300000000000
1486	0.121300000000000
1487	0.121600000000000
1488	0.121400000000000
1489	0.122100000000000
1490	0.121600000000000
1491	0.121800000000000
1492	0.121700000000000
1493	0.121200000000000
1494	0.121000000000000
1495	0.120800000000000
1496	0.120700000000000
1497	0.120500000000000
1498	0.121000000000000
1499	0.121300000000000
1500	0.121700000000000
1501	0.121500000000000
1502	0.121200000000000
1503	0.121100000000000
1504	0.120500000000000
1505	0.121200000000000
1506	0.121100000000000
1507	0.121400000000000
1508	0.121400000000000
1509	0.121800000000000
1510	0.121700000000000
1511	0.121400000000000
1512	0.121200000000000
1513	0.121300000000000
1514	0.121000000000000
1515	0.121400000000000

1516	0.121400000000000
1517	0.121300000000000
1518	0.121200000000000
1519	0.121400000000000
1520	0.121300000000000
1521	0.121000000000000
1522	0.120700000000000
1523	0.121000000000000
1524	0.120800000000000
1525	0.121000000000000
1526	0.120700000000000
1527	0.120700000000000
1528	0.121500000000000
1529	0.121400000000000
1530	0.121300000000000
1531	0.121000000000000
1532	0.121200000000000
1533	0.121000000000000
1534	0.121100000000000
1535	0.121400000000000
1536	0.121400000000000
1537	0.121300000000000
1538	0.121500000000000
1539	0.121200000000000
1540	0.120900000000000
1541	0.121000000000000
1542	0.121000000000000
1543	0.121100000000000
1544	0.121200000000000
1545	0.121400000000000
1546	0.121900000000000
1547	0.121900000000000
1548	0.122700000000000
1549	0.122800000000000

1550	0.123400000000000
1551	0.123200000000000
1552	0.123600000000000
1553	0.124000000000000
1554	0.124300000000000
1555	0.124800000000000
1556	0.124900000000000
1557	0.125500000000000
1558	0.125800000000000
1559	0.126000000000000
1560	0.126200000000000
1561	0.126300000000000
1562	0.126000000000000
1563	0.126400000000000
1564	0.126600000000000
1565	0.126500000000000
1566	0.126800000000000
1567	0.127400000000000
1568	0.127700000000000
1569	0.128100000000000
1570	0.129100000000000
1571	0.128800000000000
1572	0.128800000000000
1573	0.128900000000000
1574	0.129000000000000
1575	0.129200000000000
1576	0.129800000000000
1577	0.130100000000000
1578	0.130100000000000
1579	0.130200000000000
1580	0.130300000000000
1581	0.130700000000000
1582	0.131100000000000
1583	0.131100000000000

1584	0.131400000000000
1585	0.131400000000000
1586	0.131800000000000
1587	0.132200000000000
1588	0.132400000000000
1589	0.133000000000000
1590	0.133500000000000
1591	0.132900000000000
1592	0.133100000000000
1593	0.133800000000000
1594	0.134000000000000
1595	0.134200000000000
1596	0.134500000000000
1597	0.134500000000000
1598	0.134900000000000
1599	0.134800000000000
1600	0.135800000000000
1601	0.136000000000000
1602	0.136500000000000
1603	0.136900000000000
1604	0.137100000000000
1605	0.137200000000000
1606	0.137200000000000
1607	0.137400000000000
1608	0.137600000000000
1609	0.138600000000000
1610	0.138600000000000
1611	0.138500000000000
1612	0.138500000000000
1613	0.138700000000000
1614	0.138200000000000
1615	0.138400000000000
1616	0.138800000000000
1617	0.138800000000000

1618	0.139400000000000
1619	0.139300000000000
1620	0.140000000000000
1621	0.140000000000000
1622	0.141400000000000
1623	0.141500000000000
1624	0.141500000000000
1625	0.141500000000000
1626	0.141600000000000
1627	0.141900000000000
1628	0.142400000000000
1629	0.142700000000000
1630	0.142600000000000
1631	0.143300000000000
1632	0.143300000000000
1633	0.143500000000000
1634	0.143400000000000
1635	0.144100000000000
1636	0.144500000000000
1637	0.144500000000000
1638	0.144300000000000
1639	0.144400000000000
1640	0.144600000000000
1641	0.145200000000000
1642	0.145300000000000
1643	0.145500000000000
1644	0.145600000000000
1645	0.145900000000000
1646	0.146100000000000
1647	0.146500000000000
1648	0.147000000000000
1649	0.147200000000000
1650	0.146800000000000
1651	0.147300000000000

1652	0.147100000000000
1653	0.147700000000000
1654	0.147800000000000
1655	0.147700000000000
1656	0.147900000000000
1657	0.148500000000000
1658	0.148800000000000
1659	0.149000000000000
1660	0.149300000000000
1661	0.149600000000000
1662	0.150100000000000
1663	0.150000000000000
1664	0.150200000000000
1665	0.150600000000000
1666	0.150700000000000
1667	0.151500000000000
1668	0.151200000000000
1669	0.151000000000000
1670	0.151600000000000
1671	0.151600000000000
1672	0.151700000000000
1673	0.152300000000000
1674	0.152500000000000
1675	0.152900000000000
1676	0.152600000000000
1677	0.153300000000000
1678	0.153400000000000
1679	0.153600000000000
1680	0.153700000000000
1681	0.153700000000000
1682	0.153800000000000
1683	0.153900000000000
1684	0.154600000000000
1685	0.154700000000000

1686	0.155300000000000
1687	0.155400000000000
1688	0.155300000000000
1689	0.155700000000000
1690	0.155300000000000
1691	0.156000000000000
1692	0.155800000000000
1693	0.156400000000000
1694	0.156500000000000
1695	0.156800000000000
1696	0.157000000000000
1697	0.157200000000000
1698	0.157800000000000
1699	0.157600000000000
1700	0.158200000000000
1701	0.158400000000000
1702	0.158200000000000
1703	0.158300000000000
1704	0.158500000000000
1705	0.159000000000000
1706	0.158900000000000
1707	0.159600000000000
1708	0.159900000000000
1709	0.159900000000000
1710	0.160300000000000
1711	0.160200000000000
1712	0.160500000000000
1713	0.161000000000000
1714	0.161200000000000
1715	0.161700000000000
1716	0.162000000000000
1717	0.162500000000000
1718	0.162300000000000
1719	0.162500000000000

1720	0.162700000000000
1721	0.162500000000000
1722	0.163000000000000
1723	0.162900000000000
1724	0.163200000000000
1725	0.163400000000000
1726	0.163800000000000
1727	0.163800000000000
1728	0.163600000000000
1729	0.164500000000000
1730	0.164800000000000
1731	0.164900000000000
1732	0.165300000000000
1733	0.165200000000000
1734	0.165300000000000
1735	0.165500000000000
1736	0.165500000000000
1737	0.165800000000000
1738	0.166000000000000
1739	0.166400000000000
1740	0.167200000000000
1741	0.167100000000000
1742	0.166800000000000
1743	0.166700000000000
1744	0.166900000000000
1745	0.167000000000000
1746	0.167500000000000
1747	0.167700000000000
1748	0.168000000000000
1749	0.168000000000000
1750	0.168500000000000
1751	0.168800000000000
1752	0.169300000000000
1753	0.169100000000000

1754	0.169100000000000
1755	0.169700000000000
1756	0.169900000000000
1757	0.169600000000000
1758	0.169800000000000
1759	0.170000000000000
1760	0.170300000000000
1761	0.170400000000000
1762	0.170400000000000
1763	0.170300000000000
1764	0.170800000000000
1765	0.170900000000000
1766	0.171200000000000
1767	0.171600000000000
1768	0.171600000000000
1769	0.172200000000000
1770	0.172200000000000
1771	0.172700000000000
1772	0.172500000000000
1773	0.172800000000000
1774	0.172900000000000
1775	0.172900000000000
1776	0.173000000000000
1777	0.173000000000000
1778	0.173300000000000
1779	0.173500000000000
1780	0.173800000000000
1781	0.174100000000000
1782	0.174300000000000
1783	0.174500000000000
1784	0.174100000000000
1785	0.174700000000000
1786	0.174900000000000
1787	0.175000000000000

1788	0.175300000000000
1789	0.175000000000000
1790	0.175300000000000
1791	0.175400000000000
1792	0.175500000000000
1793	0.176100000000000
1794	0.176400000000000
1795	0.176600000000000
1796	0.177100000000000
1797	0.176900000000000
1798	0.176800000000000
1799	0.177000000000000
1800	0.177000000000000
1801	0.177300000000000
1802	0.177200000000000
1803	0.177400000000000
1804	0.177500000000000
1805	0.177700000000000
1806	0.177900000000000
1807	0.178000000000000
1808	0.178300000000000
1809	0.178100000000000
1810	0.178600000000000
1811	0.178800000000000
1812	0.178500000000000
1813	0.180000000000000
1814	0.180300000000000
1815	0.179500000000000
1816	0.179500000000000
1817	0.179400000000000
1818	0.180000000000000
1819	0.179800000000000
1820	0.179800000000000
1821	0.180000000000000

1822	0.180400000000000
1823	0.180500000000000
1824	0.180600000000000
1825	0.180800000000000
1826	0.181200000000000
1827	0.181100000000000
1828	0.181300000000000
1829	0.181600000000000
1830	0.181900000000000
1831	0.182200000000000
1832	0.182400000000000
1833	0.181800000000000
1834	0.182500000000000
1835	0.183000000000000
1836	0.182500000000000
1837	0.182500000000000
1838	0.182800000000000
1839	0.183200000000000
1840	0.183000000000000
1841	0.183500000000000
1842	0.183000000000000
1843	0.183600000000000
1844	0.184200000000000
1845	0.184300000000000
1846	0.184100000000000
1847	0.183800000000000
1848	0.184400000000000
1849	0.184300000000000
1850	0.184500000000000
1851	0.184700000000000
1852	0.185000000000000
1853	0.185100000000000
1854	0.185200000000000
1855	0.185400000000000

1856	0.185400000000000
1857	0.185400000000000
1858	0.185800000000000
1859	0.186100000000000
1860	0.186000000000000
1861	0.186400000000000
1862	0.186400000000000
1863	0.186000000000000
1864	0.186100000000000
1865	0.186600000000000
1866	0.186400000000000
1867	0.186500000000000
1868	0.186600000000000
1869	0.187100000000000
1870	0.187200000000000
1871	0.187300000000000
1872	0.187500000000000
1873	0.187300000000000
1874	0.187300000000000
1875	0.187400000000000
1876	0.187600000000000
1877	0.188100000000000
1878	0.188500000000000
1879	0.188200000000000
1880	0.188100000000000
1881	0.188000000000000
1882	0.188200000000000
1883	0.188900000000000
1884	0.188800000000000
1885	0.188900000000000
1886	0.189100000000000
1887	0.189200000000000
1888	0.189400000000000
1889	0.189100000000000

1890	0.189200000000000
1891	0.189000000000000
1892	0.189700000000000
1893	0.190100000000000
1894	0.189700000000000
1895	0.189700000000000
1896	0.190000000000000
1897	0.190600000000000
1898	0.190400000000000
1899	0.190000000000000
1900	0.190700000000000
1901	0.190500000000000
1902	0.190600000000000
1903	0.190600000000000
1904	0.190600000000000
1905	0.190700000000000
1906	0.191200000000000
1907	0.191700000000000
1908	0.192200000000000
1909	0.191600000000000
1910	0.191800000000000
1911	0.192100000000000
1912	0.192000000000000
1913	0.191800000000000
1914	0.191900000000000
1915	0.191800000000000
1916	0.192400000000000
1917	0.192700000000000
1918	0.192400000000000
1919	0.192200000000000
1920	0.192500000000000
1921	0.193500000000000
1922	0.193300000000000
1923	0.193200000000000

1924	0.193400000000000
1925	0.193300000000000
1926	0.193300000000000
1927	0.193200000000000
1928	0.192900000000000
1929	0.193600000000000
1930	0.193700000000000
1931	0.193700000000000
1932	0.193700000000000
1933	0.193800000000000
1934	0.194100000000000
1935	0.194200000000000
1936	0.194300000000000
1937	0.194300000000000
1938	0.194500000000000
1939	0.194500000000000
1940	0.194400000000000
1941	0.194300000000000
1942	0.194300000000000
1943	0.194900000000000
1944	0.194900000000000
1945	0.194500000000000
1946	0.194800000000000
1947	0.195300000000000
1948	0.195200000000000
1949	0.195200000000000
1950	0.195300000000000
1951	0.195300000000000
1952	0.196100000000000
1953	0.195500000000000
1954	0.195300000000000
1955	0.195200000000000
1956	0.195700000000000
1957	0.195800000000000

1958	0.196200000000000
1959	0.196300000000000
1960	0.196200000000000
1961	0.196100000000000
1962	0.196200000000000
1963	0.196200000000000
1964	0.196400000000000
1965	0.196300000000000
1966	0.196100000000000
1967	0.196400000000000
1968	0.196200000000000
1969	0.196400000000000
1970	0.196700000000000
1971	0.196900000000000
1972	0.197600000000000
1973	0.197300000000000
1974	0.196800000000000
1975	0.196500000000000
1976	0.197200000000000
1977	0.197100000000000
1978	0.197200000000000
1979	0.197400000000000
1980	0.197300000000000
1981	0.197200000000000
1982	0.197300000000000
1983	0.197700000000000
1984	0.197500000000000
1985	0.197600000000000
1986	0.198100000000000
1987	0.198600000000000
1988	0.198700000000000
1989	0.198100000000000
1990	0.198200000000000
1991	0.197800000000000

1992	0.198000000000000
1993	0.198000000000000
1994	0.198200000000000
1995	0.198200000000000
1996	0.198200000000000
1997	0.198600000000000
1998	0.198600000000000
1999	0.198200000000000
2000	0.198900000000000
2001	0.199300000000000
2002	0.199100000000000
2003	0.198800000000000
2004	0.198900000000000
2005	0.199100000000000
2006	0.198900000000000
2007	0.199300000000000
2008	0.199300000000000
2009	0.199200000000000
2010	0.199100000000000
2011	0.199100000000000
2012	0.199700000000000
2013	0.199600000000000
2014	0.199800000000000
2015	0.199800000000000
2016	0.200000000000000
2017	0.200200000000000
2018	0.200000000000000
2019	0.199700000000000
2020	0.199800000000000
2021	0.199800000000000
2022	0.199600000000000
2023	0.200000000000000
2024	0.199800000000000
2025	0.200100000000000

2026	0.199800000000000
2027	0.200500000000000
2028	0.200600000000000
2029	0.200300000000000
2030	0.200300000000000
2031	0.200500000000000
2032	0.201000000000000
2033	0.201000000000000
2034	0.200700000000000
2035	0.200600000000000
2036	0.200400000000000
2037	0.200600000000000
2038	0.200600000000000
2039	0.201000000000000
2040	0.200500000000000
2041	0.200400000000000
2042	0.200900000000000
2043	0.201400000000000
2044	0.201400000000000
2045	0.201400000000000
2046	0.201000000000000
2047	0.201000000000000
2048	0.201400000000000
2049	0.201400000000000
2050	0.201400000000000
2051	0.201400000000000
2052	0.201200000000000
2053	0.201500000000000
2054	0.201600000000000
2055	0.201300000000000
2056	0.201300000000000
2057	0.201300000000000
2058	0.201400000000000
2059	0.202000000000000

2060	0.201700000000000
2061	0.202100000000000
2062	0.201900000000000
2063	0.202300000000000
2064	0.202500000000000
2065	0.202400000000000
2066	0.202200000000000
2067	0.202900000000000
2068	0.202500000000000
2069	0.202200000000000
2070	0.202200000000000
2071	0.202300000000000
2072	0.202400000000000
2073	0.202500000000000
2074	0.202200000000000
2075	0.202400000000000
2076	0.202600000000000
2077	0.203000000000000
2078	0.202500000000000
2079	0.202600000000000
2080	0.202500000000000
2081	0.203100000000000
2082	0.202600000000000
2083	0.202600000000000
2084	0.203200000000000
2085	0.202900000000000
2086	0.203000000000000
2087	0.203000000000000
2088	0.203000000000000
2089	0.203500000000000
2090	0.203000000000000
2091	0.203400000000000
2092	0.203100000000000
2093	0.203800000000000

2094	0.203900000000000
2095	0.203400000000000
2096	0.203300000000000
2097	0.203700000000000
2098	0.203700000000000
2099	0.204100000000000
2100	0.204200000000000
2101	0.204200000000000
2102	0.203900000000000
2103	0.204200000000000
2104	0.204200000000000
2105	0.204000000000000
2106	0.204000000000000
2107	0.204100000000000
2108	0.204300000000000
2109	0.204700000000000
2110	0.204800000000000
2111	0.204900000000000
2112	0.205000000000000
2113	0.204500000000000
2114	0.204800000000000
2115	0.204900000000000
2116	0.204700000000000
2117	0.204900000000000
2118	0.205200000000000
2119	0.204800000000000
2120	0.205100000000000
2121	0.205000000000000
2122	0.204900000000000
2123	0.205000000000000
2124	0.205000000000000
2125	0.205000000000000
2126	0.205500000000000
2127	0.205500000000000

2128	0.205300000000000
2129	0.205200000000000
2130	0.205300000000000
2131	0.205400000000000
2132	0.205000000000000
2133	0.205500000000000
2134	0.205500000000000
2135	0.205700000000000
2136	0.205900000000000
2137	0.206200000000000
2138	0.206100000000000
2139	0.205800000000000
2140	0.206100000000000
2141	0.206100000000000
2142	0.205900000000000
2143	0.206400000000000
2144	0.206500000000000
2145	0.206100000000000
2146	0.206100000000000
2147	0.205900000000000
2148	0.206200000000000
2149	0.206100000000000
2150	0.206500000000000
2151	0.206400000000000
2152	0.206400000000000
2153	0.206300000000000
2154	0.206500000000000
2155	0.206700000000000
2156	0.206800000000000
2157	0.206600000000000
2158	0.206500000000000
2159	0.206500000000000
2160	0.206800000000000
2161	0.206500000000000

2162	0.206900000000000
2163	0.207000000000000
2164	0.207000000000000
2165	0.207000000000000
2166	0.207100000000000
2167	0.207400000000000
2168	0.207200000000000
2169	0.207400000000000
2170	0.207700000000000
2171	0.207800000000000
2172	0.207500000000000
2173	0.207600000000000
2174	0.207700000000000
2175	0.207600000000000
2176	0.207500000000000
2177	0.207400000000000
2178	0.207500000000000
2179	0.207800000000000
2180	0.208000000000000
2181	0.208100000000000
2182	0.208000000000000
2183	0.207900000000000
2184	0.208300000000000
2185	0.208000000000000
2186	0.208100000000000
2187	0.208500000000000
2188	0.208400000000000
2189	0.208300000000000
2190	0.208500000000000
2191	0.208300000000000
2192	0.208400000000000
2193	0.208300000000000
2194	0.208400000000000
2195	0.208600000000000

2196	0.208400000000000
2197	0.208700000000000
2198	0.209300000000000
2199	0.209500000000000
2200	0.208900000000000
2201	0.208600000000000
2202	0.208700000000000
2203	0.209000000000000
2204	0.209600000000000
2205	0.208900000000000
2206	0.209200000000000
2207	0.208800000000000
2208	0.208900000000000
2209	0.208900000000000
2210	0.209100000000000
2211	0.209200000000000
2212	0.209400000000000
2213	0.209600000000000
2214	0.209300000000000
2215	0.209600000000000
2216	0.209400000000000
2217	0.209900000000000
2218	0.209700000000000
2219	0.210000000000000
2220	0.209700000000000
2221	0.209700000000000
2222	0.209400000000000
2223	0.209700000000000
2224	0.209400000000000
2225	0.210200000000000
2226	0.209700000000000
2227	0.210100000000000
2228	0.209800000000000
2229	0.210000000000000

2230	0.210200000000000
2231	0.210400000000000
2232	0.210500000000000
2233	0.210500000000000
2234	0.210100000000000
2235	0.210200000000000
2236	0.210300000000000
2237	0.210900000000000
2238	0.210700000000000
2239	0.210500000000000
2240	0.210800000000000
2241	0.210900000000000
2242	0.210600000000000
2243	0.210800000000000
2244	0.210500000000000
2245	0.211000000000000
2246	0.211300000000000
2247	0.210900000000000
2248	0.211000000000000
2249	0.211100000000000
2250	0.211200000000000
2251	0.211400000000000
2252	0.211300000000000
2253	0.210900000000000
2254	0.211000000000000
2255	0.211200000000000
2256	0.211400000000000
2257	0.211400000000000
2258	0.211600000000000
2259	0.211500000000000
2260	0.211400000000000
2261	0.211400000000000
2262	0.211400000000000
2263	0.211700000000000

2264	0.211800000000000
2265	0.211800000000000
2266	0.211900000000000
2267	0.211900000000000
2268	0.212000000000000
2269	0.211700000000000
2270	0.211900000000000
2271	0.211700000000000
2272	0.211900000000000
2273	0.211700000000000
2274	0.212200000000000
2275	0.212100000000000
2276	0.211900000000000
2277	0.212300000000000
2278	0.212200000000000
2279	0.212100000000000
2280	0.212100000000000
2281	0.212500000000000
2282	0.212200000000000
2283	0.212400000000000
2284	0.212400000000000
2285	0.212600000000000
2286	0.212300000000000
2287	0.212700000000000
2288	0.212400000000000
2289	0.212600000000000
2290	0.212700000000000
2291	0.212700000000000
2292	0.212900000000000
2293	0.213000000000000
2294	0.212700000000000
2295	0.213000000000000
2296	0.212800000000000
2297	0.212900000000000

2298	0.212700000000000
2299	0.212900000000000
2300	0.213100000000000
2301	0.213000000000000
2302	0.213100000000000
2303	0.213000000000000
2304	0.213200000000000
2305	0.213300000000000
2306	0.213400000000000
2307	0.213200000000000
2308	0.213300000000000
2309	0.213300000000000
2310	0.213900000000000
2311	0.213900000000000
2312	0.214000000000000
2313	0.214300000000000
2314	0.213800000000000
2315	0.213700000000000
2316	0.213700000000000
2317	0.214000000000000
2318	0.214100000000000
2319	0.214300000000000
2320	0.214100000000000
2321	0.213900000000000
2322	0.214100000000000
2323	0.214300000000000
2324	0.214400000000000
2325	0.214600000000000
2326	0.214200000000000
2327	0.214000000000000
2328	0.214000000000000
2329	0.214600000000000
2330	0.214200000000000
2331	0.214600000000000

2332	0.214300000000000
2333	0.214400000000000
2334	0.214500000000000
2335	0.214800000000000
2336	0.214600000000000
2337	0.214400000000000
2338	0.214600000000000
2339	0.214800000000000
2340	0.214900000000000
2341	0.215100000000000
2342	0.215100000000000
2343	0.215200000000000
2344	0.214800000000000
2345	0.214800000000000
2346	0.214800000000000
2347	0.214600000000000
2348	0.215000000000000
2349	0.215100000000000
2350	0.215100000000000
2351	0.215400000000000
2352	0.215400000000000
2353	0.215600000000000
2354	0.215500000000000
2355	0.215700000000000
2356	0.215400000000000
2357	0.215700000000000
2358	0.215500000000000
2359	0.215700000000000
2360	0.215600000000000
2361	0.215600000000000
2362	0.215500000000000
2363	0.215700000000000
2364	0.215600000000000
2365	0.215900000000000

2366	0.215900000000000
2367	0.215300000000000
2368	0.215700000000000
2369	0.215400000000000
2370	0.215800000000000
2371	0.216000000000000
2372	0.216000000000000
2373	0.216000000000000
2374	0.216200000000000
2375	0.216200000000000
2376	0.216200000000000
2377	0.216100000000000
2378	0.216300000000000
2379	0.216000000000000
2380	0.216100000000000
2381	0.216400000000000
2382	0.216200000000000
2383	0.216300000000000
2384	0.216200000000000
2385	0.216400000000000
2386	0.216400000000000
2387	0.216400000000000
2388	0.216400000000000
2389	0.216200000000000
2390	0.217000000000000
2391	0.216900000000000
2392	0.216600000000000
2393	0.216500000000000
2394	0.217100000000000
2395	0.217000000000000
2396	0.216900000000000
2397	0.217100000000000
2398	0.217300000000000
2399	0.217300000000000

2400	0.217500000000000
2401	0.217200000000000
2402	0.217100000000000
2403	0.217200000000000
2404	0.217200000000000
2405	0.217300000000000
2406	0.217100000000000
2407	0.217200000000000
2408	0.217100000000000
2409	0.217400000000000
2410	0.217200000000000
2411	0.217800000000000
2412	0.217300000000000
2413	0.218000000000000
2414	0.217700000000000
2415	0.217900000000000
2416	0.217600000000000
2417	0.217700000000000
2418	0.217700000000000
2419	0.217900000000000
2420	0.217800000000000
2421	0.218000000000000
2422	0.218100000000000
2423	0.218300000000000
2424	0.218600000000000
2425	0.218400000000000
2426	0.218300000000000
2427	0.218300000000000
2428	0.218100000000000
2429	0.218000000000000
2430	0.218200000000000
2431	0.218100000000000
2432	0.218400000000000
2433	0.218400000000000

2434	0.218400000000000
2435	0.218400000000000
2436	0.218600000000000
2437	0.218300000000000
2438	0.218000000000000
2439	0.218400000000000
2440	0.218400000000000
2441	0.218500000000000
2442	0.218300000000000
2443	0.218400000000000
2444	0.218600000000000
2445	0.218900000000000
2446	0.218900000000000
2447	0.218900000000000
2448	0.219100000000000
2449	0.218600000000000
2450	0.218800000000000
2451	0.219100000000000
2452	0.218800000000000
2453	0.219100000000000
2454	0.219100000000000
2455	0.219200000000000
2456	0.219200000000000
2457	0.219200000000000
2458	0.219700000000000
2459	0.219300000000000
2460	0.219500000000000
2461	0.219300000000000
2462	0.219200000000000
2463	0.219300000000000
2464	0.219000000000000
2465	0.219500000000000
2466	0.220000000000000
2467	0.220000000000000

2468	0.220100000000000
2469	0.220200000000000
2470	0.220000000000000
2471	0.219700000000000
2472	0.219500000000000
2473	0.219800000000000
2474	0.219500000000000
2475	0.219700000000000
2476	0.220100000000000
2477	0.219600000000000
2478	0.219900000000000
2479	0.219900000000000
2480	0.220200000000000
2481	0.220100000000000
2482	0.219700000000000
2483	0.220200000000000
2484	0.220200000000000
2485	0.220200000000000
2486	0.219700000000000
2487	0.219800000000000
2488	0.220000000000000
2489	0.220000000000000
2490	0.220000000000000
2491	0.220000000000000
2492	0.220400000000000
2493	0.220000000000000
2494	0.220200000000000
2495	0.219700000000000
2496	0.220300000000000
2497	0.220400000000000
2498	0.220100000000000
2499	0.220400000000000
2500	0.220600000000000
2501	0.220700000000000

2502	0.220200000000000
2503	0.220400000000000
2504	0.220300000000000
2505	0.220200000000000
2506	0.220300000000000
2507	0.220900000000000
2508	0.220400000000000
2509	0.220700000000000
2510	0.220600000000000
2511	0.220900000000000
2512	0.221100000000000
2513	0.220800000000000
2514	0.221100000000000
2515	0.220900000000000
2516	0.221500000000000
2517	0.221100000000000
2518	0.220900000000000
2519	0.221100000000000
2520	0.221500000000000
2521	0.221100000000000
2522	0.221200000000000
2523	0.221100000000000
2524	0.221100000000000
2525	0.221200000000000
2526	0.221300000000000
2527	0.221400000000000
2528	0.220900000000000
2529	0.221300000000000
2530	0.221000000000000
2531	0.221700000000000
2532	0.221400000000000
2533	0.221800000000000
2534	0.221500000000000
2535	0.221100000000000

2536	0.221800000000000
2537	0.221700000000000
2538	0.221700000000000
2539	0.222000000000000
2540	0.222000000000000
2541	0.221800000000000
2542	0.222000000000000
2543	0.221600000000000
2544	0.221300000000000
2545	0.222200000000000
2546	0.222000000000000
2547	0.221600000000000
2548	0.221600000000000
2549	0.221700000000000
2550	0.221600000000000
2551	0.221900000000000
2552	0.221700000000000
2553	0.222100000000000
2554	0.221900000000000
2555	0.221600000000000
2556	0.222000000000000
2557	0.222100000000000
2558	0.221700000000000
2559	0.222000000000000
2560	0.222200000000000
2561	0.222500000000000
2562	0.222200000000000
2563	0.222000000000000
2564	0.222200000000000
2565	0.222200000000000
2566	0.222000000000000
2567	0.222300000000000
2568	0.222500000000000
2569	0.221900000000000

2570	0.222200000000000
2571	0.221900000000000
2572	0.222200000000000
2573	0.222600000000000
2574	0.222600000000000
2575	0.222300000000000
2576	0.222200000000000
2577	0.222000000000000
2578	0.222400000000000
2579	0.222300000000000
2580	0.222700000000000
2581	0.222500000000000
2582	0.222500000000000
2583	0.222700000000000
2584	0.222700000000000
2585	0.222600000000000
2586	0.222400000000000
2587	0.222400000000000
2588	0.223000000000000
2589	0.222600000000000
2590	0.222800000000000
2591	0.222900000000000
2592	0.222900000000000
2593	0.222900000000000
2594	0.222900000000000
2595	0.223000000000000
2596	0.222900000000000
2597	0.223300000000000
2598	0.223200000000000
2599	0.223200000000000
2600	0.222700000000000
2601	0.222900000000000
2602	0.222900000000000
2603	0.222700000000000

2604	0.223100000000000
2605	0.223000000000000
2606	0.223300000000000
2607	0.223500000000000
2608	0.223900000000000
2609	0.223500000000000
2610	0.223100000000000
2611	0.223400000000000
2612	0.223200000000000
2613	0.223400000000000
2614	0.223100000000000
2615	0.223000000000000
2616	0.223600000000000
2617	0.223400000000000
2618	0.223700000000000
2619	0.223800000000000
2620	0.223700000000000
2621	0.223900000000000
2622	0.223900000000000
2623	0.223500000000000
2624	0.223900000000000
2625	0.224300000000000
2626	0.223800000000000
2627	0.223800000000000
2628	0.223900000000000
2629	0.224000000000000
2630	0.224000000000000
2631	0.224400000000000
2632	0.224200000000000
2633	0.224100000000000
2634	0.224200000000000
2635	0.224300000000000
2636	0.223600000000000
2637	0.224300000000000

2638	0.224200000000000
2639	0.224100000000000
2640	0.224200000000000
2641	0.224200000000000
2642	0.224300000000000
2643	0.224000000000000
2644	0.224000000000000
2645	0.224600000000000
2646	0.224200000000000
2647	0.224800000000000
2648	0.224400000000000
2649	0.224400000000000
2650	0.224500000000000
2651	0.224600000000000
2652	0.224600000000000
2653	0.224700000000000
2654	0.224900000000000
2655	0.224800000000000
2656	0.224900000000000
2657	0.224700000000000
2658	0.224600000000000
2659	0.224700000000000
2660	0.224600000000000
2661	0.224900000000000
2662	0.225000000000000
2663	0.224900000000000
2664	0.224700000000000
2665	0.225200000000000
2666	0.225100000000000
2667	0.224900000000000
2668	0.224700000000000
2669	0.225100000000000
2670	0.225200000000000
2671	0.225100000000000

2672	0.225100000000000
2673	0.225200000000000
2674	0.225400000000000
2675	0.225100000000000
2676	0.225500000000000
2677	0.225700000000000
2678	0.225600000000000
2679	0.225500000000000
2680	0.225600000000000
2681	0.225700000000000
2682	0.225800000000000
2683	0.225700000000000
2684	0.226000000000000
2685	0.226300000000000
2686	0.225800000000000
2687	0.225800000000000
2688	0.225900000000000
2689	0.226200000000000
2690	0.226200000000000
2691	0.226100000000000
2692	0.226200000000000
2693	0.225900000000000
2694	0.226300000000000
2695	0.226100000000000
2696	0.226400000000000
2697	0.226500000000000
2698	0.226200000000000
2699	0.226500000000000
2700	0.226500000000000
2701	0.226700000000000
2702	0.226500000000000
2703	0.226800000000000
2704	0.226800000000000
2705	0.226600000000000

2706	0.226900000000000
2707	0.226600000000000
2708	0.226400000000000
2709	0.227100000000000
2710	0.227400000000000
2711	0.227500000000000
2712	0.227200000000000
2713	0.227600000000000
2714	0.227400000000000
2715	0.227700000000000
2716	0.227500000000000
2717	0.227500000000000
2718	0.227600000000000
2719	0.227900000000000
2720	0.227800000000000
2721	0.227600000000000
2722	0.228000000000000
2723	0.227500000000000
2724	0.227700000000000
2725	0.228100000000000
2726	0.228000000000000
2727	0.227800000000000
2728	0.228500000000000
2729	0.227800000000000
2730	0.228700000000000
2731	0.228500000000000
2732	0.229100000000000
2733	0.228500000000000
2734	0.228800000000000
2735	0.229100000000000
2736	0.228900000000000
2737	0.228900000000000
2738	0.229200000000000
2739	0.229000000000000

2740	0.229300000000000
2741	0.229500000000000
2742	0.229500000000000
2743	0.229500000000000
2744	0.229200000000000
2745	0.229700000000000
2746	0.230000000000000
2747	0.229900000000000
2748	0.229400000000000
2749	0.229700000000000
2750	0.229300000000000
2751	0.230000000000000
2752	0.229900000000000
2753	0.229900000000000
2754	0.229900000000000
2755	0.230500000000000
2756	0.230500000000000
2757	0.230600000000000
2758	0.230600000000000
2759	0.230900000000000
2760	0.231000000000000
2761	0.230800000000000
2762	0.230700000000000
2763	0.230800000000000
2764	0.230700000000000
2765	0.230900000000000
2766	0.231100000000000
2767	0.231000000000000
2768	0.231300000000000
2769	0.231500000000000
2770	0.231500000000000
2771	0.231500000000000
2772	0.231800000000000
2773	0.231900000000000

2774	0.231600000000000
2775	0.232000000000000
2776	0.231800000000000
2777	0.232000000000000
2778	0.232100000000000
2779	0.232000000000000
2780	0.231900000000000
2781	0.232100000000000
2782	0.232200000000000
2783	0.232600000000000
2784	0.232800000000000
2785	0.232700000000000
2786	0.232800000000000
2787	0.233000000000000
2788	0.233400000000000
2789	0.233500000000000
2790	0.233200000000000
2791	0.233400000000000
2792	0.233100000000000
2793	0.233400000000000
2794	0.233000000000000
2795	0.233300000000000
2796	0.233300000000000
2797	0.233700000000000
2798	0.233600000000000
2799	0.234100000000000
2800	0.234000000000000
2801	0.234200000000000
2802	0.234400000000000
2803	0.234300000000000
2804	0.234300000000000
2805	0.234700000000000
2806	0.234800000000000
2807	0.234400000000000

2808	0.234700000000000
2809	0.234700000000000
2810	0.235100000000000
2811	0.235000000000000
2812	0.235600000000000
2813	0.235000000000000
2814	0.235000000000000
2815	0.234900000000000
2816	0.235500000000000
2817	0.235800000000000
2818	0.235900000000000
2819	0.236000000000000
2820	0.235800000000000
2821	0.236100000000000
2822	0.236100000000000
2823	0.236100000000000
2824	0.236400000000000
2825	0.236100000000000
2826	0.236700000000000
2827	0.236700000000000
2828	0.236900000000000
2829	0.236900000000000
2830	0.237100000000000
2831	0.237200000000000
2832	0.237100000000000
2833	0.237200000000000
2834	0.237300000000000
2835	0.236900000000000
2836	0.237100000000000
2837	0.237400000000000
2838	0.237600000000000
2839	0.237500000000000
2840	0.237600000000000
2841	0.237200000000000

2842	0.237100000000000
2843	0.237100000000000
2844	0.237100000000000
2845	0.237700000000000
2846	0.237500000000000
2847	0.237500000000000
2848	0.237800000000000
2849	0.237500000000000
2850	0.237900000000000
2851	0.238100000000000
2852	0.238400000000000
2853	0.158800000000000
2854	0.142800000000000
2855	0.141600000000000
2856	0.140100000000000
2857	0.140100000000000
2858	0.140000000000000
2859	0.139500000000000
2860	0.139300000000000
2861	0.137400000000000
2862	0.136600000000000
2863	0.136400000000000
2864	0.136500000000000
2865	0.136200000000000
2866	0.136500000000000
2867	0.136000000000000
2868	0.136500000000000
2869	0.136400000000000
2870	0.135900000000000
2871	0.135900000000000
2872	0.136600000000000
2873	0.136800000000000
2874	0.137300000000000
2875	0.137800000000000

2876	0.137000000000000
2877	0.136800000000000
2878	0.136600000000000
2879	0.137100000000000
2880	0.137200000000000
2881	0.137300000000000
2882	0.136900000000000
2883	0.136700000000000
2884	0.136300000000000
2885	0.136200000000000
2886	0.136400000000000
2887	0.136100000000000
2888	0.136200000000000
2889	0.136200000000000
2890	0.135700000000000
2891	0.135800000000000
2892	0.135800000000000
2893	0.135500000000000
2894	0.135700000000000
2895	0.136200000000000
2896	0.136500000000000
2897	0.136200000000000
2898	0.136200000000000
2899	0.135800000000000
2900	0.135500000000000
2901	0.135300000000000
2902	0.135100000000000
2903	0.135700000000000
2904	0.135900000000000
2905	0.136200000000000
2906	0.136000000000000
2907	0.136000000000000
2908	0.136300000000000
2909	0.136600000000000

2910	0.137100000000000
2911	0.136700000000000
2912	0.136500000000000
2913	0.136400000000000
2914	0.136200000000000
2915	0.136100000000000
2916	0.136500000000000
2917	0.136300000000000
2918	0.136300000000000
2919	0.136100000000000
2920	0.135800000000000
2921	0.135600000000000
2922	0.135200000000000
2923	0.135100000000000
2924	0.135600000000000
2925	0.135600000000000
2926	0.135300000000000
2927	0.135400000000000
2928	0.135600000000000
2929	0.136300000000000
2930	0.136500000000000
2931	0.136500000000000
2932	0.136600000000000
2933	0.136000000000000
2934	0.135600000000000
2935	0.135200000000000
2936	0.135100000000000
2937	0.135100000000000
2938	0.135300000000000
2939	0.135600000000000
2940	0.135200000000000
2941	0.135000000000000
2942	0.134600000000000
2943	0.134600000000000

2944	0.134600000000000
2945	0.134900000000000
2946	0.135400000000000
2947	0.135100000000000
2948	0.135200000000000
2949	0.135400000000000
2950	0.135200000000000
2951	0.135000000000000
2952	0.135000000000000
2953	0.134900000000000
2954	0.135000000000000
2955	0.134900000000000
2956	0.134800000000000
2957	0.135000000000000
2958	0.135000000000000
2959	0.135000000000000
2960	0.134800000000000
2961	0.134700000000000
2962	0.134600000000000
2963	0.134900000000000
2964	0.135000000000000
2965	0.134900000000000
2966	0.134800000000000
2967	0.134800000000000
2968	0.134900000000000
2969	0.134900000000000
2970	0.134700000000000
2971	0.134900000000000
2972	0.134800000000000
2973	0.134400000000000
2974	0.134400000000000
2975	0.134700000000000
2976	0.134400000000000
2977	0.134400000000000

2978	0.134700000000000
2979	0.134300000000000
2980	0.134000000000000
2981	0.133900000000000
2982	0.134400000000000
2983	0.134400000000000
2984	0.134500000000000
2985	0.134400000000000
2986	0.134500000000000
2987	0.134200000000000
2988	0.134200000000000
2989	0.134400000000000
2990	0.134400000000000
2991	0.134300000000000
2992	0.134300000000000
2993	0.134300000000000
2994	0.134300000000000
2995	0.134300000000000
2996	0.134200000000000
2997	0.134600000000000
2998	0.134100000000000
2999	0.134600000000000
3000	0.134400000000000
3001	0.134500000000000
3002	0.135000000000000
3003	0.134900000000000
3004	0.135400000000000
3005	0.135000000000000
3006	0.135500000000000
3007	0.135700000000000
3008	0.136000000000000
3009	0.136400000000000
3010	0.136500000000000
3011	0.136900000000000

3012	0.137300000000000
3013	0.137200000000000
3014	0.137600000000000
3015	0.138000000000000
3016	0.138400000000000
3017	0.138800000000000
3018	0.138900000000000
3019	0.139700000000000
3020	0.140200000000000
3021	0.140100000000000
3022	0.140500000000000
3023	0.140600000000000
3024	0.140900000000000
3025	0.141300000000000
3026	0.141900000000000
3027	0.142100000000000
3028	0.141900000000000
3029	0.142500000000000
3030	0.142900000000000
3031	0.143500000000000
3032	0.143500000000000
3033	0.143700000000000
3034	0.143900000000000
3035	0.144400000000000
3036	0.144500000000000
3037	0.144900000000000
3038	0.145400000000000
3039	0.145500000000000
3040	0.145900000000000
3041	0.146400000000000
3042	0.146300000000000
3043	0.147000000000000
3044	0.147500000000000
3045	0.147600000000000

3046	0.147700000000000
3047	0.147800000000000
3048	0.148000000000000
3049	0.148500000000000
3050	0.149100000000000
3051	0.149100000000000
3052	0.149600000000000
3053	0.149700000000000
3054	0.149700000000000
3055	0.149900000000000
3056	0.149700000000000
3057	0.150300000000000
3058	0.150600000000000
3059	0.150700000000000
3060	0.150700000000000
3061	0.151300000000000
3062	0.151900000000000
3063	0.151800000000000
3064	0.151800000000000
3065	0.152300000000000
3066	0.152900000000000
3067	0.153100000000000
3068	0.153300000000000
3069	0.153500000000000
3070	0.153800000000000
3071	0.154100000000000
3072	0.154300000000000
3073	0.154200000000000
3074	0.154800000000000
3075	0.155200000000000
3076	0.155700000000000
3077	0.155800000000000
3078	0.156100000000000
3079	0.156300000000000

3080	0.156800000000000
3081	0.156800000000000
3082	0.157300000000000
3083	0.157500000000000
3084	0.157400000000000
3085	0.158100000000000
3086	0.158500000000000
3087	0.158700000000000
3088	0.158500000000000
3089	0.158900000000000
3090	0.159000000000000
3091	0.159300000000000
3092	0.159500000000000
3093	0.160000000000000
3094	0.160500000000000
3095	0.160600000000000
3096	0.161200000000000
3097	0.161200000000000
3098	0.161600000000000
3099	0.161700000000000
3100	0.162000000000000
3101	0.162700000000000
3102	0.162700000000000
3103	0.162600000000000
3104	0.163000000000000
3105	0.162800000000000
3106	0.163500000000000
3107	0.163600000000000
3108	0.164300000000000
3109	0.164400000000000
3110	0.165000000000000
3111	0.164900000000000
3112	0.165900000000000
3113	0.165500000000000

3114	0.165800000000000
3115	0.166100000000000
3116	0.166400000000000
3117	0.167000000000000
3118	0.167300000000000
3119	0.167400000000000
3120	0.167600000000000
3121	0.167800000000000
3122	0.168000000000000
3123	0.168400000000000
3124	0.168300000000000
3125	0.169100000000000
3126	0.168900000000000
3127	0.169400000000000
3128	0.169900000000000
3129	0.170300000000000
3130	0.170300000000000
3131	0.170600000000000
3132	0.170600000000000
3133	0.170800000000000
3134	0.171500000000000
3135	0.171800000000000
3136	0.172000000000000
3137	0.172200000000000
3138	0.172000000000000
3139	0.172600000000000
3140	0.172500000000000
3141	0.173100000000000
3142	0.173900000000000
3143	0.174500000000000
3144	0.174300000000000
3145	0.174900000000000
3146	0.174300000000000
3147	0.174500000000000

3148	0.175000000000000
3149	0.174800000000000
3150	0.175900000000000
3151	0.175900000000000
3152	0.175600000000000
3153	0.176100000000000
3154	0.176500000000000
3155	0.176700000000000
3156	0.176500000000000
3157	0.177000000000000
3158	0.177500000000000
3159	0.178100000000000
3160	0.178100000000000
3161	0.178800000000000
3162	0.179300000000000
3163	0.179100000000000
3164	0.179000000000000
3165	0.179700000000000
3166	0.179700000000000
3167	0.179900000000000
3168	0.180000000000000
3169	0.180200000000000
3170	0.180500000000000
3171	0.181100000000000
3172	0.181200000000000
3173	0.181300000000000
3174	0.181400000000000
3175	0.181400000000000
3176	0.181900000000000
3177	0.182500000000000
3178	0.182800000000000
3179	0.182900000000000
3180	0.183200000000000
3181	0.183300000000000

3182	0.183600000000000
3183	0.183500000000000
3184	0.184300000000000
3185	0.184500000000000
3186	0.184900000000000
3187	0.185100000000000
3188	0.185200000000000
3189	0.185200000000000
3190	0.185200000000000
3191	0.185100000000000
3192	0.185900000000000
3193	0.186000000000000
3194	0.186400000000000
3195	0.186800000000000
3196	0.186800000000000
3197	0.187100000000000
3198	0.187400000000000
3199	0.187000000000000
3200	0.187800000000000
3201	0.188000000000000
3202	0.188500000000000
3203	0.188600000000000
3204	0.188700000000000
3205	0.189100000000000
3206	0.189000000000000
3207	0.189100000000000
3208	0.189200000000000
3209	0.189700000000000
3210	0.190000000000000
3211	0.190500000000000
3212	0.190500000000000
3213	0.190600000000000
3214	0.190900000000000
3215	0.190800000000000

3216	0.191200000000000
3217	0.190900000000000
3218	0.191500000000000
3219	0.191700000000000
3220	0.192200000000000
3221	0.192500000000000
3222	0.192900000000000
3223	0.192700000000000
3224	0.192800000000000
3225	0.192900000000000
3226	0.193400000000000
3227	0.194100000000000
3228	0.194100000000000
3229	0.194600000000000
3230	0.194300000000000
3231	0.194200000000000
3232	0.194300000000000
3233	0.194800000000000
3234	0.194800000000000
3235	0.194900000000000
3236	0.195700000000000
3237	0.195700000000000
3238	0.195700000000000
3239	0.195800000000000
3240	0.196100000000000
3241	0.196300000000000
3242	0.196200000000000
3243	0.196900000000000
3244	0.197100000000000
3245	0.197300000000000
3246	0.197200000000000
3247	0.197600000000000
3248	0.197800000000000
3249	0.197800000000000

3250	0.198100000000000
3251	0.198400000000000
3252	0.198500000000000
3253	0.198700000000000
3254	0.198600000000000
3255	0.199200000000000
3256	0.199100000000000
3257	0.199300000000000
3258	0.199400000000000
3259	0.199800000000000
3260	0.200100000000000
3261	0.199900000000000
3262	0.200400000000000
3263	0.201000000000000
3264	0.201400000000000
3265	0.201600000000000
3266	0.201300000000000
3267	0.201700000000000
3268	0.201800000000000
3269	0.202000000000000
3270	0.202400000000000
3271	0.202100000000000
3272	0.202700000000000
3273	0.202400000000000
3274	0.203100000000000
3275	0.203100000000000
3276	0.203400000000000
3277	0.203600000000000
3278	0.203500000000000
3279	0.203900000000000
3280	0.203900000000000
3281	0.204300000000000
3282	0.204200000000000
3283	0.204800000000000

3284	0.204300000000000
3285	0.204300000000000
3286	0.204600000000000
3287	0.205000000000000
3288	0.204900000000000
3289	0.205400000000000
3290	0.205500000000000
3291	0.205800000000000
3292	0.205600000000000
3293	0.205900000000000
3294	0.206000000000000
3295	0.206300000000000
3296	0.206600000000000
3297	0.207000000000000
3298	0.206700000000000
3299	0.207100000000000
3300	0.207400000000000
3301	0.207300000000000
3302	0.207400000000000
3303	0.207100000000000
3304	0.207800000000000
3305	0.207600000000000
3306	0.208100000000000
3307	0.208000000000000
3308	0.208200000000000
3309	0.208600000000000
3310	0.209100000000000
3311	0.209000000000000
3312	0.209400000000000
3313	0.209100000000000
3314	0.209800000000000
3315	0.209600000000000
3316	0.209800000000000
3317	0.209800000000000

3318	0.210200000000000
3319	0.210200000000000
3320	0.210900000000000
3321	0.210500000000000
3322	0.210600000000000
3323	0.210800000000000
3324	0.210800000000000
3325	0.211100000000000
3326	0.211100000000000
3327	0.211300000000000
3328	0.211500000000000
3329	0.211900000000000
3330	0.211900000000000
3331	0.212000000000000
3332	0.212400000000000
3333	0.212800000000000
3334	0.212700000000000
3335	0.212900000000000
3336	0.212600000000000
3337	0.212800000000000
3338	0.212900000000000
3339	0.213200000000000
3340	0.213700000000000
3341	0.213500000000000
3342	0.213700000000000
3343	0.213500000000000
3344	0.214100000000000
3345	0.214200000000000
3346	0.214200000000000
3347	0.214300000000000
3348	0.214400000000000
3349	0.214900000000000
3350	0.214700000000000
3351	0.215200000000000

3352	0.215300000000000
3353	0.215600000000000
3354	0.215400000000000
3355	0.215400000000000
3356	0.216000000000000
3357	0.216400000000000
3358	0.216500000000000
3359	0.216500000000000
3360	0.216400000000000
3361	0.216600000000000
3362	0.216200000000000
3363	0.216700000000000
3364	0.216900000000000
3365	0.216900000000000
3366	0.216800000000000
3367	0.217500000000000
3368	0.217500000000000
3369	0.217900000000000
3370	0.217700000000000
3371	0.217600000000000
3372	0.217500000000000
3373	0.217700000000000
3374	0.218200000000000
3375	0.218000000000000
3376	0.218500000000000
3377	0.218700000000000
3378	0.218700000000000
3379	0.218900000000000
3380	0.218900000000000
3381	0.219000000000000
3382	0.219400000000000
3383	0.219500000000000
3384	0.219200000000000
3385	0.219300000000000

3386	0.219600000000000
3387	0.219800000000000
3388	0.219700000000000
3389	0.219700000000000
3390	0.219800000000000
3391	0.220300000000000
3392	0.220400000000000
3393	0.221000000000000
3394	0.220500000000000
3395	0.221100000000000
3396	0.221100000000000
3397	0.221300000000000
3398	0.220600000000000
3399	0.221000000000000
3400	0.221400000000000
3401	0.221000000000000
3402	0.220900000000000
3403	0.221100000000000
3404	0.221600000000000
3405	0.221500000000000
3406	0.222000000000000
3407	0.221700000000000
3408	0.221700000000000
3409	0.222100000000000
3410	0.222300000000000
3411	0.222100000000000
3412	0.222300000000000
3413	0.222400000000000
3414	0.222600000000000
3415	0.222300000000000
3416	0.222200000000000
3417	0.222300000000000
3418	0.222600000000000
3419	0.222700000000000

3420	0.222800000000000
3421	0.222600000000000
3422	0.223000000000000
3423	0.222600000000000
3424	0.223400000000000
3425	0.223400000000000
3426	0.223500000000000
3427	0.223200000000000
3428	0.223600000000000
3429	0.223600000000000
3430	0.223500000000000
3431	0.223500000000000
3432	0.222900000000000
3433	0.223600000000000
3434	0.223600000000000
3435	0.223500000000000
3436	0.223500000000000
3437	0.223800000000000
3438	0.223900000000000
3439	0.224300000000000
3440	0.224100000000000
3441	0.224300000000000
3442	0.224300000000000
3443	0.224200000000000
3444	0.224200000000000
3445	0.224200000000000
3446	0.224700000000000
3447	0.224500000000000
3448	0.224700000000000
3449	0.224900000000000
3450	0.225200000000000
3451	0.225000000000000
3452	0.224700000000000
3453	0.224600000000000

3454	0.224700000000000
3455	0.224800000000000
3456	0.225200000000000
3457	0.225100000000000
3458	0.225800000000000
3459	0.225200000000000
3460	0.225700000000000
3461	0.225300000000000
3462	0.225500000000000
3463	0.225800000000000
3464	0.225700000000000
3465	0.225800000000000
3466	0.225800000000000
3467	0.226100000000000
3468	0.225700000000000
3469	0.225900000000000
3470	0.226200000000000
3471	0.226200000000000
3472	0.226200000000000
3473	0.226200000000000
3474	0.226500000000000
3475	0.226200000000000
3476	0.226700000000000
3477	0.226300000000000
3478	0.226400000000000
3479	0.226600000000000
3480	0.226700000000000
3481	0.226900000000000
3482	0.226800000000000
3483	0.227000000000000
3484	0.226700000000000
3485	0.226900000000000
3486	0.226900000000000
3487	0.226800000000000

3488	0.227400000000000
3489	0.227500000000000
3490	0.227600000000000
3491	0.227800000000000
3492	0.227900000000000
3493	0.227300000000000
3494	0.227400000000000
3495	0.227300000000000
3496	0.227600000000000
3497	0.227600000000000
3498	0.227800000000000
3499	0.227500000000000
3500	0.227300000000000
3501	0.227600000000000
3502	0.228000000000000
3503	0.228000000000000
3504	0.227900000000000
3505	0.227800000000000
3506	0.228100000000000
3507	0.227900000000000
3508	0.228100000000000
3509	0.228300000000000
3510	0.228100000000000
3511	0.228500000000000
3512	0.228500000000000
3513	0.228300000000000
3514	0.228500000000000
3515	0.228700000000000
3516	0.228700000000000
3517	0.228600000000000
3518	0.229000000000000
3519	0.229000000000000
3520	0.229000000000000
3521	0.228400000000000

3522	0.229000000000000
3523	0.229100000000000
3524	0.229200000000000
3525	0.228900000000000
3526	0.229100000000000
3527	0.229300000000000
3528	0.229400000000000
3529	0.229700000000000
3530	0.229400000000000
3531	0.229200000000000
3532	0.229100000000000
3533	0.229700000000000
3534	0.229600000000000
3535	0.229200000000000
3536	0.229400000000000
3537	0.229700000000000
3538	0.229500000000000
3539	0.229800000000000
3540	0.229500000000000
3541	0.230100000000000
3542	0.229900000000000
3543	0.230000000000000
3544	0.230200000000000
3545	0.230200000000000
3546	0.230400000000000
3547	0.230600000000000
3548	0.230300000000000
3549	0.230600000000000
3550	0.230900000000000
3551	0.230800000000000
3552	0.230800000000000
3553	0.230600000000000
3554	0.230700000000000
3555	0.230500000000000

3556	0.230700000000000
3557	0.230800000000000
3558	0.230900000000000
3559	0.231000000000000
3560	0.231100000000000
3561	0.230700000000000
3562	0.231000000000000
3563	0.231400000000000
3564	0.231500000000000
3565	0.231500000000000
3566	0.231600000000000
3567	0.231700000000000
3568	0.232400000000000
3569	0.231900000000000
3570	0.231600000000000
3571	0.232200000000000
3572	0.232300000000000
3573	0.232400000000000
3574	0.232000000000000
3575	0.231800000000000
3576	0.232100000000000
3577	0.231700000000000
3578	0.232100000000000
3579	0.232200000000000
3580	0.232200000000000
3581	0.232100000000000
3582	0.232500000000000
3583	0.232800000000000
3584	0.232500000000000
3585	0.232400000000000
3586	0.232600000000000
3587	0.232900000000000
3588	0.232700000000000
3589	0.232600000000000

3590	0.233400000000000
3591	0.232900000000000
3592	0.233300000000000
3593	0.233100000000000
3594	0.233300000000000
3595	0.232900000000000
3596	0.233300000000000
3597	0.233700000000000
3598	0.233400000000000
3599	0.233800000000000
3600	0.234100000000000
3601	0.233600000000000
3602	0.233700000000000
3603	0.233400000000000
3604	0.233400000000000
3605	0.233800000000000
3606	0.233700000000000
3607	0.233600000000000
3608	0.233900000000000
3609	0.234200000000000
3610	0.234200000000000
3611	0.233900000000000
3612	0.234400000000000
3613	0.233700000000000
3614	0.234100000000000
3615	0.234100000000000
3616	0.234500000000000
3617	0.234800000000000
3618	0.234500000000000
3619	0.234700000000000
3620	0.234500000000000
3621	0.234700000000000
3622	0.234500000000000
3623	0.234700000000000

3624	0.234400000000000
3625	0.234500000000000
3626	0.234800000000000
3627	0.235400000000000
3628	0.235300000000000
3629	0.235300000000000
3630	0.235200000000000
3631	0.234900000000000
3632	0.235200000000000
3633	0.235600000000000
3634	0.235400000000000
3635	0.235200000000000
3636	0.235200000000000
3637	0.235800000000000
3638	0.235600000000000
3639	0.235900000000000
3640	0.236300000000000
3641	0.235800000000000
3642	0.235800000000000
3643	0.235900000000000
3644	0.235500000000000
3645	0.235700000000000
3646	0.236200000000000
3647	0.236300000000000
3648	0.236200000000000
3649	0.236000000000000
3650	0.236100000000000
3651	0.236100000000000
3652	0.236300000000000
3653	0.236700000000000
3654	0.236700000000000
3655	0.236600000000000
3656	0.236400000000000
3657	0.237000000000000

3658	0.237200000000000
3659	0.236600000000000
3660	0.236700000000000
3661	0.237000000000000
3662	0.236800000000000
3663	0.236500000000000
3664	0.236800000000000
3665	0.237000000000000
3666	0.236700000000000
3667	0.236800000000000
3668	0.236800000000000
3669	0.236800000000000
3670	0.237000000000000
3671	0.237100000000000
3672	0.237000000000000
3673	0.237200000000000
3674	0.237400000000000
3675	0.237600000000000
3676	0.237400000000000
3677	0.237700000000000
3678	0.237400000000000
3679	0.237400000000000
3680	0.237500000000000
3681	0.237500000000000
3682	0.237500000000000
3683	0.237900000000000
3684	0.237600000000000
3685	0.237700000000000
3686	0.237400000000000
3687	0.237700000000000
3688	0.237900000000000
3689	0.237800000000000
3690	0.237900000000000
3691	0.238100000000000

3692	0.238000000000000
3693	0.237800000000000
3694	0.238100000000000
3695	0.238200000000000
3696	0.238100000000000
3697	0.238100000000000
3698	0.238700000000000
3699	0.238300000000000
3700	0.238400000000000
3701	0.238800000000000
3702	0.238500000000000
3703	0.238400000000000
3704	0.238200000000000
3705	0.238600000000000
3706	0.238600000000000
3707	0.238900000000000
3708	0.238900000000000
3709	0.238900000000000
3710	0.238900000000000
3711	0.239000000000000
3712	0.239400000000000
3713	0.239300000000000
3714	0.239300000000000
3715	0.239200000000000
3716	0.239300000000000
3717	0.239300000000000
3718	0.239100000000000
3719	0.239200000000000
3720	0.239400000000000
3721	0.239100000000000
3722	0.239500000000000
3723	0.239200000000000
3724	0.239100000000000
3725	0.239600000000000

3726	0.239900000000000
3727	0.239500000000000
3728	0.239800000000000
3729	0.239500000000000
3730	0.240100000000000
3731	0.240100000000000
3732	0.240400000000000
3733	0.240100000000000
3734	0.240000000000000
3735	0.240100000000000
3736	0.240300000000000
3737	0.240100000000000
3738	0.240300000000000
3739	0.239900000000000
3740	0.240100000000000
3741	0.239900000000000
3742	0.240500000000000
3743	0.240300000000000
3744	0.240100000000000
3745	0.240200000000000
3746	0.240300000000000
3747	0.240500000000000
3748	0.240400000000000
3749	0.240400000000000
3750	0.240500000000000
3751	0.240500000000000
3752	0.240600000000000
3753	0.240700000000000
3754	0.241000000000000
3755	0.241200000000000
3756	0.241000000000000
3757	0.241100000000000
3758	0.241100000000000
3759	0.241100000000000

3760	0.241200000000000
3761	0.241000000000000
3762	0.241300000000000
3763	0.241500000000000
3764	0.241300000000000
3765	0.241100000000000
3766	0.241300000000000
3767	0.241300000000000
3768	0.241200000000000
3769	0.241200000000000
3770	0.241300000000000
3771	0.241600000000000
3772	0.241600000000000
3773	0.242000000000000
3774	0.241700000000000
3775	0.242100000000000
3776	0.242000000000000
3777	0.241800000000000
3778	0.242000000000000
3779	0.241700000000000
3780	0.241700000000000
3781	0.242200000000000
3782	0.241900000000000
3783	0.241800000000000
3784	0.242100000000000
3785	0.242000000000000
3786	0.242100000000000
3787	0.242100000000000
3788	0.242100000000000
3789	0.241700000000000
3790	0.242000000000000
3791	0.242500000000000
3792	0.242200000000000
3793	0.242400000000000

3794	0.242500000000000
3795	0.242400000000000
3796	0.242300000000000
3797	0.242700000000000
3798	0.242200000000000
3799	0.242700000000000
3800	0.242600000000000
3801	0.242800000000000
3802	0.242500000000000
3803	0.242700000000000
3804	0.243100000000000
3805	0.243000000000000
3806	0.242700000000000
3807	0.242900000000000
3808	0.242700000000000
3809	0.243000000000000
3810	0.242800000000000
3811	0.243100000000000
3812	0.243000000000000
3813	0.243100000000000
3814	0.243100000000000
3815	0.242800000000000
3816	0.243200000000000
3817	0.243100000000000
3818	0.243000000000000
3819	0.243100000000000
3820	0.243000000000000
3821	0.242900000000000
3822	0.243400000000000
3823	0.243400000000000
3824	0.243300000000000
3825	0.243700000000000
3826	0.243500000000000
3827	0.243500000000000

3828	0.243300000000000
3829	0.243100000000000
3830	0.243500000000000
3831	0.243500000000000
3832	0.243500000000000
3833	0.243400000000000
3834	0.243900000000000
3835	0.243700000000000
3836	0.243800000000000
3837	0.244000000000000
3838	0.243900000000000
3839	0.244100000000000
3840	0.243900000000000
3841	0.243900000000000
3842	0.244100000000000
3843	0.244000000000000
3844	0.243900000000000
3845	0.244100000000000
3846	0.244200000000000
3847	0.244200000000000
3848	0.243800000000000
3849	0.244100000000000
3850	0.244100000000000
3851	0.243600000000000
3852	0.244200000000000
3853	0.243900000000000
3854	0.244000000000000
3855	0.244200000000000
3856	0.244500000000000
3857	0.244600000000000
3858	0.244500000000000
3859	0.244700000000000
3860	0.244300000000000
3861	0.244500000000000

3862	0.244400000000000
3863	0.244400000000000
3864	0.244200000000000
3865	0.244800000000000
3866	0.244600000000000
3867	0.244700000000000
3868	0.245000000000000
3869	0.244500000000000
3870	0.244800000000000
3871	0.244800000000000
3872	0.244500000000000
3873	0.244800000000000
3874	0.244800000000000
3875	0.244900000000000
3876	0.245100000000000
3877	0.244900000000000
3878	0.244800000000000
3879	0.245200000000000
3880	0.245300000000000
3881	0.245200000000000
3882	0.245300000000000
3883	0.245200000000000
3884	0.245500000000000
3885	0.245400000000000
3886	0.245600000000000
3887	0.245400000000000
3888	0.245500000000000
3889	0.245600000000000
3890	0.245600000000000
3891	0.245400000000000
3892	0.245600000000000
3893	0.245600000000000
3894	0.245500000000000
3895	0.245600000000000

3896	0.245200000000000
3897	0.245700000000000
3898	0.245600000000000
3899	0.245600000000000
3900	0.245500000000000
3901	0.245600000000000
3902	0.245700000000000
3903	0.245900000000000
3904	0.245700000000000
3905	0.246300000000000
3906	0.246000000000000
3907	0.245900000000000
3908	0.245900000000000
3909	0.245800000000000
3910	0.246300000000000
3911	0.246100000000000
3912	0.246100000000000
3913	0.246100000000000
3914	0.246300000000000
3915	0.246400000000000
3916	0.246300000000000
3917	0.246300000000000
3918	0.246400000000000
3919	0.246300000000000
3920	0.246300000000000
3921	0.246400000000000
3922	0.246700000000000
3923	0.246400000000000
3924	0.246400000000000
3925	0.246400000000000
3926	0.246400000000000
3927	0.246600000000000
3928	0.246600000000000
3929	0.246800000000000

3930	0.246900000000000
3931	0.246400000000000
3932	0.246800000000000
3933	0.246800000000000
3934	0.247200000000000
3935	0.246900000000000
3936	0.247000000000000
3937	0.247000000000000
3938	0.246900000000000
3939	0.247100000000000
3940	0.247200000000000
3941	0.247100000000000
3942	0.247100000000000
3943	0.247300000000000
3944	0.247400000000000
3945	0.246900000000000
3946	0.247300000000000
3947	0.247200000000000
3948	0.247300000000000
3949	0.247700000000000
3950	0.247600000000000
3951	0.247100000000000
3952	0.247800000000000
3953	0.247700000000000
3954	0.248000000000000
3955	0.247900000000000
3956	0.248000000000000
3957	0.248000000000000
3958	0.247800000000000
3959	0.248200000000000
3960	0.248200000000000
3961	0.247800000000000
3962	0.248000000000000
3963	0.248300000000000

3964	0.248100000000000
3965	0.248100000000000
3966	0.248000000000000
3967	0.248200000000000
3968	0.248400000000000
3969	0.248400000000000
3970	0.248900000000000
3971	0.248800000000000
3972	0.248500000000000
3973	0.248700000000000
3974	0.248900000000000
3975	0.248700000000000
3976	0.248500000000000
3977	0.249000000000000
3978	0.249000000000000
3979	0.249100000000000
3980	0.248700000000000
3981	0.249000000000000
3982	0.248900000000000
3983	0.249200000000000
3984	0.248900000000000
3985	0.249000000000000
3986	0.249300000000000
3987	0.249300000000000
3988	0.249400000000000
3989	0.249400000000000
3990	0.249600000000000
3991	0.249300000000000
3992	0.249400000000000
3993	0.249700000000000
3994	0.249400000000000
3995	0.249300000000000
3996	0.249500000000000
3997	0.249700000000000

3998	0.249500000000000
3999	0.249600000000000
4000	0.249700000000000
4001	0.249800000000000
4002	0.249700000000000
4003	0.249900000000000
4004	0.250200000000000
4005	0.250200000000000
4006	0.249800000000000
4007	0.250200000000000
4008	0.250200000000000
4009	0.250100000000000
4010	0.250400000000000
4011	0.250300000000000
4012	0.250500000000000
4013	0.250500000000000
4014	0.250500000000000
4015	0.250500000000000
4016	0.250700000000000
4017	0.250500000000000
4018	0.250600000000000
4019	0.250500000000000
4020	0.250500000000000
4021	0.250700000000000
4022	0.250500000000000
4023	0.250700000000000
4024	0.250700000000000
4025	0.250700000000000
4026	0.251600000000000
4027	0.251200000000000
4028	0.250900000000000
4029	0.250900000000000
4030	0.251300000000000
4031	0.251100000000000

4032	0.251200000000000
4033	0.251500000000000
4034	0.251400000000000
4035	0.251200000000000
4036	0.251200000000000
4037	0.251400000000000
4038	0.251300000000000
4039	0.251300000000000
4040	0.251500000000000
4041	0.251700000000000
4042	0.252200000000000
4043	0.251600000000000
4044	0.251600000000000
4045	0.252000000000000
4046	0.251800000000000
4047	0.251900000000000
4048	0.251600000000000
4049	0.252000000000000
4050	0.252000000000000
4051	0.252000000000000
4052	0.252500000000000
4053	0.252200000000000
4054	0.252400000000000
4055	0.252400000000000
4056	0.252200000000000
4057	0.252500000000000
4058	0.252400000000000
4059	0.252200000000000
4060	0.252800000000000
4061	0.252500000000000
4062	0.252500000000000
4063	0.252700000000000
4064	0.252000000000000
4065	0.252400000000000

4066	0.252500000000000
4067	0.252700000000000
4068	0.252900000000000
4069	0.253100000000000
4070	0.253200000000000
4071	0.252800000000000
4072	0.252800000000000
4073	0.253200000000000
4074	0.253300000000000
4075	0.253100000000000
4076	0.252900000000000
4077	0.253200000000000
4078	0.253100000000000
4079	0.253200000000000
4080	0.253300000000000
4081	0.253300000000000
4082	0.253200000000000
4083	0.252800000000000
4084	0.253300000000000
4085	0.253300000000000
4086	0.253100000000000
4087	0.253000000000000
4088	0.253400000000000
4089	0.253500000000000
4090	0.253600000000000
4091	0.253500000000000
4092	0.253800000000000
4093	0.254000000000000
4094	0.254200000000000
4095	0.253900000000000
4096	0.253800000000000
4097	0.254100000000000
4098	0.253700000000000
4099	0.253800000000000

4100	0.253800000000000
4101	0.253800000000000
4102	0.253800000000000
4103	0.254400000000000
4104	0.253800000000000
4105	0.254100000000000
4106	0.253900000000000
4107	0.254400000000000
4108	0.253900000000000
4109	0.254500000000000
4110	0.254300000000000
4111	0.254500000000000
4112	0.254600000000000
4113	0.255200000000000
4114	0.255000000000000
4115	0.254900000000000
4116	0.255000000000000
4117	0.255300000000000
4118	0.255200000000000
4119	0.255400000000000
4120	0.255200000000000
4121	0.255600000000000
4122	0.255300000000000
4123	0.255300000000000
4124	0.255500000000000
4125	0.255400000000000
4126	0.255500000000000
4127	0.255600000000000
4128	0.255900000000000
4129	0.256100000000000
4130	0.256000000000000
4131	0.256100000000000
4132	0.256200000000000
4133	0.256000000000000

4134	0.256100000000000
4135	0.256200000000000
4136	0.256600000000000
4137	0.256600000000000
4138	0.256700000000000
4139	0.257000000000000
4140	0.256600000000000
4141	0.256800000000000
4142	0.257000000000000
4143	0.256800000000000
4144	0.257100000000000
4145	0.257100000000000
4146	0.257600000000000
4147	0.257500000000000
4148	0.257300000000000
4149	0.257300000000000
4150	0.257900000000000
4151	0.258100000000000
4152	0.257800000000000
4153	0.257700000000000
4154	0.258200000000000
4155	0.258100000000000
4156	0.258100000000000
4157	0.258100000000000
4158	0.258400000000000
4159	0.258800000000000
4160	0.258600000000000
4161	0.258900000000000
4162	0.258700000000000
4163	0.258700000000000
4164	0.258600000000000
4165	0.259000000000000
4166	0.259000000000000
4167	0.259100000000000

4168	0.259300000000000
4169	0.259200000000000
4170	0.259000000000000
4171	0.258800000000000
4172	0.259000000000000
4173	0.259000000000000
4174	0.259500000000000
4175	0.259400000000000
4176	0.259400000000000
4177	0.259400000000000
4178	0.259600000000000
4179	0.259800000000000
4180	0.259700000000000
4181	0.260400000000000
4182	0.260000000000000
4183	0.260400000000000
4184	0.260000000000000
4185	0.260200000000000
4186	0.260500000000000
4187	0.260600000000000
4188	0.260500000000000
4189	0.260400000000000
4190	0.260700000000000
4191	0.260300000000000
4192	0.260400000000000
4193	0.260600000000000
4194	0.260800000000000
4195	0.260600000000000
4196	0.260500000000000
4197	0.260900000000000
4198	0.260800000000000
4199	0.261200000000000
4200	0.261100000000000
4201	0.260800000000000

4202	0.261200000000000
4203	0.261500000000000
4204	0.261500000000000
4205	0.261400000000000
4206	0.261000000000000
4207	0.261400000000000
4208	0.261600000000000
4209	0.261600000000000
4210	0.261100000000000
4211	0.261300000000000
4212	0.261600000000000
4213	0.261600000000000
4214	0.261800000000000
4215	0.262200000000000
4216	0.262000000000000
4217	0.261700000000000
4218	0.261600000000000
4219	0.261600000000000
4220	0.262200000000000
4221	0.261800000000000
4222	0.262000000000000
4223	0.262100000000000
4224	0.261900000000000
4225	0.262900000000000
4226	0.262600000000000
4227	0.262300000000000
4228	0.262700000000000
4229	0.262200000000000
4230	0.262400000000000
4231	0.262600000000000
4232	0.263000000000000
4233	0.262800000000000
4234	0.262700000000000
4235	0.262600000000000

4236	0.263200000000000
4237	0.263200000000000
4238	0.263200000000000
4239	0.263100000000000
4240	0.263300000000000
4241	0.263000000000000
4242	0.263100000000000
4243	0.263200000000000
4244	0.263500000000000
4245	0.263400000000000
4246	0.263600000000000
4247	0.263200000000000
4248	0.263200000000000
4249	0.263600000000000
4250	0.263700000000000
4251	0.263400000000000
4252	0.264000000000000
4253	0.263900000000000
4254	0.263700000000000
4255	0.264000000000000
4256	0.263900000000000
4257	0.263800000000000
4258	0.264100000000000
4259	0.264300000000000
4260	0.264100000000000
4261	0.264200000000000
4262	0.264700000000000
4263	0.264500000000000
4264	0.264600000000000
4265	0.264600000000000
4266	0.264400000000000
4267	0.264200000000000
4268	0.264600000000000
4269	0.264600000000000

4270	0.264600000000000
4271	0.264600000000000
4272	0.265000000000000
4273	0.265000000000000
4274	0.265200000000000
4275	0.265000000000000
4276	0.265700000000000
4277	0.265300000000000
4278	0.265500000000000
4279	0.265000000000000
4280	0.265600000000000
4281	0.265600000000000

1-Cu^{II} 1.0 mM, K_2CO_3 10 mM, HPNP 0.20 mM, TCA 5mM

t(min)	A_{400nm} (a.u.)
0	0.312500000000000
1	0.293000000000000
2	0.289400000000000
3	0.289800000000000
4	0.290300000000000
5	0.290500000000000
6	0.291600000000000
7	0.290800000000000
8	0.290900000000000
9	0.291600000000000
10	0.293100000000000
11	0.293500000000000
12	0.294000000000000
13	0.294800000000000
14	0.295000000000000
15	0.296100000000000
16	0.296800000000000
17	0.297100000000000

18	0.297100000000000
19	0.298100000000000
20	0.299300000000000
21	0.299400000000000
22	0.299800000000000
23	0.300100000000000
24	0.300200000000000
25	0.300800000000000
26	0.301600000000000
27	0.302200000000000
28	0.302500000000000
29	0.303400000000000
30	0.304000000000000
31	0.304300000000000
32	0.304800000000000
33	0.305400000000000
34	0.305900000000000
35	0.306500000000000
36	0.307700000000000
37	0.308200000000000
38	0.308500000000000
39	0.309600000000000
40	0.310200000000000
41	0.310700000000000
42	0.311200000000000
43	0.311700000000000
44	0.312200000000000
45	0.312700000000000
46	0.312900000000000
47	0.313900000000000
48	0.314500000000000
49	0.315200000000000
50	0.316100000000000
51	0.316700000000000

52	0.317000000000000
53	0.317600000000000
54	0.318200000000000
55	0.318800000000000
56	0.319200000000000
57	0.320100000000000
58	0.320900000000000
59	0.321600000000000
60	0.322000000000000
61	0.322500000000000
62	0.323200000000000
63	0.324100000000000
64	0.324400000000000
65	0.325000000000000
66	0.325700000000000
67	0.326300000000000
68	0.326800000000000
69	0.327600000000000
70	0.328400000000000
71	0.329100000000000
72	0.329700000000000
73	0.330200000000000
74	0.330900000000000
75	0.331200000000000
76	0.332000000000000
77	0.332400000000000
78	0.333000000000000
79	0.333900000000000
80	0.334300000000000
81	0.334700000000000
82	0.335600000000000
83	0.335700000000000
84	0.336900000000000
85	0.336900000000000

86	0.337900000000000
87	0.338100000000000
88	0.339000000000000
89	0.339200000000000
90	0.339900000000000
91	0.340900000000000
92	0.341700000000000
93	0.342000000000000
94	0.223500000000000
95	0.216200000000000
96	0.217700000000000
97	0.219100000000000
98	0.221500000000000
99	0.223300000000000
100	0.224900000000000
101	0.226100000000000
102	0.228200000000000
103	0.229900000000000
104	0.231700000000000
105	0.234000000000000
106	0.235500000000000
107	0.237500000000000
108	0.239200000000000
109	0.241200000000000
110	0.242700000000000
111	0.244500000000000
112	0.246900000000000
113	0.248600000000000
114	0.250100000000000
115	0.252000000000000
116	0.253300000000000
117	0.254700000000000
118	0.256300000000000
119	0.257700000000000

120	0.259100000000000
121	0.260800000000000
122	0.262800000000000
123	0.264300000000000
124	0.265600000000000
125	0.267600000000000
126	0.268800000000000
127	0.269800000000000
128	0.271400000000000
129	0.272800000000000
130	0.274100000000000
131	0.275400000000000
132	0.276400000000000
133	0.277100000000000
134	0.278800000000000
135	0.280100000000000
136	0.281500000000000
137	0.282600000000000
138	0.283900000000000
139	0.285200000000000
140	0.286500000000000
141	0.288200000000000
142	0.289200000000000
143	0.290700000000000
144	0.291500000000000
145	0.292600000000000
146	0.293100000000000
147	0.294600000000000
148	0.295200000000000
149	0.296200000000000
150	0.297600000000000
151	0.298500000000000
152	0.299900000000000
153	0.301000000000000

154	0.302300000000000
155	0.303000000000000
156	0.304100000000000
157	0.305000000000000
158	0.306300000000000
159	0.307300000000000
160	0.308300000000000
161	0.308900000000000
162	0.310500000000000
163	0.311200000000000
164	0.312000000000000
165	0.312900000000000
166	0.313700000000000
167	0.314700000000000
168	0.316200000000000
169	0.316100000000000
170	0.317600000000000
171	0.318000000000000
172	0.319600000000000
173	0.320000000000000
174	0.321100000000000
175	0.322300000000000
176	0.323100000000000
177	0.323700000000000
178	0.324500000000000
179	0.325300000000000
180	0.326500000000000
181	0.326500000000000
182	0.327800000000000
183	0.328500000000000
184	0.329700000000000
185	0.330200000000000
186	0.330800000000000
187	0.331700000000000

188	0.332200000000000
189	0.333500000000000
190	0.334500000000000
191	0.335500000000000
192	0.336300000000000
193	0.336700000000000
194	0.337900000000000
195	0.338300000000000
196	0.339000000000000
197	0.339500000000000
198	0.340400000000000
199	0.341100000000000
200	0.341600000000000
201	0.342500000000000
202	0.343400000000000
203	0.344000000000000
204	0.344700000000000
205	0.345600000000000
206	0.346200000000000
207	0.346700000000000
208	0.347700000000000
209	0.348100000000000
210	0.348700000000000
211	0.349700000000000
212	0.350100000000000
213	0.350600000000000
214	0.351500000000000
215	0.352300000000000
216	0.353100000000000
217	0.353300000000000
218	0.354200000000000
219	0.354900000000000
220	0.355600000000000
221	0.356100000000000

222	0.357200000000000
223	0.357400000000000
224	0.358000000000000
225	0.358600000000000
226	0.359500000000000
227	0.360500000000000
228	0.360200000000000
229	0.360900000000000
230	0.361400000000000
231	0.362400000000000
232	0.363000000000000
233	0.363600000000000
234	0.364200000000000
235	0.364900000000000
236	0.365700000000000
237	0.366300000000000
238	0.366900000000000
239	0.367100000000000
240	0.368100000000000
241	0.368700000000000
242	0.369000000000000
243	0.369800000000000
244	0.370300000000000
245	0.371100000000000
246	0.371200000000000
247	0.372000000000000
248	0.372700000000000
249	0.373300000000000
250	0.374200000000000
251	0.374600000000000
252	0.374900000000000
253	0.375700000000000
254	0.376500000000000
255	0.376800000000000

256	0.376700000000000
257	0.377500000000000
258	0.377800000000000
259	0.379000000000000
260	0.379500000000000
261	0.380000000000000
262	0.380800000000000
264	0.235900000000000
265	0.237300000000000
266	0.240300000000000
267	0.243600000000000
268	0.245300000000000
269	0.248500000000000
270	0.251000000000000
271	0.253500000000000
272	0.256200000000000
273	0.259000000000000
274	0.261100000000000
275	0.263200000000000
276	0.265200000000000
277	0.267700000000000
278	0.269800000000000
279	0.271600000000000
280	0.274300000000000
281	0.276400000000000
282	0.279000000000000
283	0.281400000000000
284	0.283600000000000
285	0.285600000000000
286	0.287500000000000
287	0.289900000000000
288	0.290900000000000
289	0.292200000000000
290	0.294300000000000

291	0.296000000000000
292	0.297800000000000
293	0.299500000000000
294	0.300900000000000
295	0.302700000000000
296	0.304600000000000
297	0.306400000000000
298	0.308300000000000
299	0.310000000000000
300	0.311200000000000
301	0.313000000000000
302	0.313600000000000
303	0.315400000000000
304	0.316400000000000
305	0.317700000000000
306	0.319100000000000
307	0.321000000000000
308	0.323100000000000
309	0.324400000000000
310	0.325500000000000
311	0.327000000000000
312	0.328000000000000
313	0.329500000000000
314	0.331400000000000
315	0.332300000000000
316	0.333100000000000
317	0.333900000000000
318	0.334500000000000
319	0.335300000000000
320	0.336600000000000
321	0.338000000000000
322	0.339200000000000
323	0.340100000000000
324	0.341700000000000

325	0.343400000000000
326	0.343800000000000
327	0.345000000000000
328	0.345500000000000
329	0.346300000000000
330	0.347500000000000
331	0.348500000000000
332	0.349100000000000
333	0.350400000000000
334	0.351400000000000
335	0.353100000000000
336	0.353700000000000
337	0.354100000000000
338	0.355100000000000
339	0.356200000000000
340	0.357300000000000
341	0.358600000000000
342	0.359400000000000
343	0.360800000000000
344	0.362000000000000
345	0.362100000000000
346	0.362600000000000
347	0.363500000000000
348	0.364600000000000
349	0.365300000000000
350	0.366300000000000
351	0.366700000000000
352	0.367100000000000
353	0.368000000000000
354	0.369300000000000
355	0.369900000000000
356	0.370400000000000
357	0.371100000000000
358	0.372000000000000

359	0.373500000000000
360	0.373600000000000
361	0.374400000000000
362	0.375100000000000
363	0.376100000000000
364	0.376900000000000
365	0.377200000000000
366	0.378300000000000
367	0.379300000000000
368	0.379500000000000
369	0.379900000000000
370	0.380700000000000
371	0.381300000000000
372	0.382400000000000
373	0.383200000000000
374	0.383700000000000
375	0.384300000000000
376	0.384500000000000
377	0.385300000000000
378	0.385900000000000
379	0.386900000000000
380	0.387100000000000
381	0.388200000000000
382	0.389200000000000
383	0.389500000000000
384	0.390400000000000
385	0.390700000000000
386	0.391200000000000
387	0.391300000000000
388	0.392000000000000
389	0.392900000000000
390	0.393700000000000
391	0.394200000000000
392	0.394700000000000

393	0.395400000000000
394	0.396000000000000
395	0.396900000000000
396	0.397100000000000
397	0.397400000000000
398	0.397900000000000
399	0.398300000000000
400	0.399000000000000
401	0.399800000000000
402	0.400000000000000
403	0.400700000000000
404	0.401100000000000
405	0.401300000000000
406	0.402800000000000
407	0.403000000000000
408	0.403300000000000
409	0.404100000000000
410	0.404400000000000
411	0.404600000000000
412	0.405100000000000
413	0.405600000000000
414	0.406500000000000
415	0.407700000000000
416	0.407900000000000
417	0.407800000000000
418	0.408000000000000
419	0.409100000000000
420	0.409100000000000
421	0.409900000000000
422	0.410200000000000
423	0.410300000000000
424	0.411100000000000
425	0.411600000000000
426	0.411400000000000

427	0.412500000000000
428	0.413200000000000
429	0.413500000000000
430	0.413900000000000
431	0.414100000000000
432	0.414700000000000
433	0.414900000000000
434	0.415300000000000
435	0.415600000000000
436	0.416200000000000
437	0.417300000000000
438	0.417000000000000
439	0.417700000000000
440	0.417900000000000
441	0.418600000000000
442	0.419400000000000
443	0.419100000000000
444	0.419900000000000
445	0.419800000000000
446	0.420100000000000
447	0.420400000000000
448	0.421300000000000
449	0.421900000000000
450	0.422200000000000
451	0.422200000000000
452	0.422700000000000
453	0.423400000000000
454	0.423900000000000
455	0.424100000000000
456	0.424500000000000
457	0.425100000000000
458	0.425400000000000
459	0.426200000000000
460	0.426100000000000

461	0.426600000000000
462	0.427100000000000
463	0.427400000000000
464	0.427200000000000
465	0.428100000000000
466	0.428000000000000
467	0.428500000000000
468	0.429300000000000
469	0.428900000000000
470	0.429600000000000
471	0.429700000000000
472	0.430200000000000
473	0.430300000000000
474	0.431400000000000
475	0.431300000000000
476	0.431900000000000
477	0.432600000000000
478	0.432700000000000
479	0.432700000000000
480	0.433400000000000
481	0.433400000000000
482	0.434100000000000
483	0.434600000000000
484	0.434000000000000
485	0.434700000000000
486	0.435500000000000
487	0.435600000000000
488	0.436200000000000
489	0.436500000000000
490	0.436900000000000
491	0.436900000000000
492	0.437600000000000
493	0.437900000000000
494	0.438200000000000

495	0.438500000000000
496	0.439100000000000
497	0.439100000000000
498	0.439000000000000
499	0.439300000000000
500	0.439700000000000
501	0.440200000000000
502	0.440700000000000
503	0.441000000000000
504	0.441000000000000
505	0.441300000000000
506	0.441400000000000
507	0.441800000000000
508	0.442400000000000
509	0.442500000000000
510	0.443200000000000
511	0.443000000000000
512	0.443600000000000
513	0.443800000000000
514	0.443900000000000
515	0.444500000000000
516	0.444800000000000
517	0.445100000000000
518	0.445500000000000
519	0.445400000000000
520	0.446100000000000
521	0.446300000000000
522	0.446600000000000
523	0.446700000000000
524	0.447400000000000
525	0.447300000000000
526	0.447400000000000
527	0.448500000000000
528	0.448100000000000

529	0.448200000000000
530	0.448800000000000
531	0.448500000000000
532	0.449500000000000
533	0.449900000000000
534	0.449600000000000
535	0.450100000000000
536	0.450500000000000
537	0.451200000000000
538	0.451400000000000
539	0.451400000000000
540	0.451300000000000
541	0.452200000000000
542	0.452200000000000
543	0.452500000000000
544	0.452700000000000
545	0.453100000000000
546	0.453600000000000
547	0.453800000000000
548	0.454400000000000
549	0.454100000000000
550	0.454200000000000
551	0.454300000000000
552	0.455200000000000
553	0.455500000000000
554	0.455400000000000
555	0.455800000000000
556	0.456300000000000
557	0.456700000000000
558	0.457200000000000
559	0.456800000000000
560	0.457200000000000
561	0.457900000000000
562	0.458100000000000

563	0.458100000000000
564	0.458500000000000
565	0.458900000000000
566	0.459400000000000
567	0.459200000000000
568	0.459400000000000
569	0.459800000000000
570	0.459900000000000
571	0.460400000000000
572	0.460600000000000
573	0.460400000000000
574	0.461100000000000
575	0.461800000000000
576	0.461500000000000
577	0.462000000000000
578	0.462500000000000
579	0.462500000000000
580	0.463100000000000
581	0.462700000000000
582	0.463300000000000
583	0.463200000000000
584	0.463600000000000
585	0.463500000000000
586	0.464100000000000
587	0.464400000000000
588	0.464200000000000
589	0.465400000000000
590	0.465000000000000
591	0.465600000000000
592	0.465900000000000
593	0.465800000000000
594	0.465900000000000
595	0.466800000000000
596	0.466700000000000

597	0.467100000000000
598	0.467500000000000
599	0.467600000000000
600	0.467900000000000
601	0.468100000000000
602	0.468300000000000
603	0.468400000000000
604	0.468900000000000
605	0.468800000000000
606	0.469200000000000
607	0.469500000000000
608	0.469700000000000
609	0.470300000000000
610	0.470200000000000
611	0.470300000000000
612	0.470700000000000
613	0.471500000000000
614	0.471800000000000
615	0.471600000000000
616	0.472000000000000
617	0.472400000000000
618	0.472400000000000
619	0.472700000000000
620	0.473300000000000
621	0.473500000000000
622	0.473500000000000
623	0.473700000000000
624	0.473800000000000
625	0.473800000000000
626	0.473900000000000
627	0.474400000000000
628	0.474700000000000
629	0.475000000000000
630	0.475700000000000

631	0.475800000000000
632	0.476000000000000
633	0.475900000000000
634	0.476300000000000
635	0.476700000000000
636	0.477000000000000
637	0.477300000000000
638	0.478000000000000
639	0.478000000000000
640	0.478000000000000
641	0.478100000000000
642	0.477900000000000
643	0.478300000000000
644	0.478800000000000
645	0.478900000000000
646	0.479200000000000
647	0.479500000000000
648	0.479500000000000
649	0.480300000000000
650	0.480500000000000
651	0.480100000000000
652	0.480900000000000
653	0.481100000000000
654	0.481200000000000
655	0.481800000000000
656	0.481800000000000
657	0.482200000000000
658	0.482100000000000
659	0.482200000000000
660	0.482600000000000
661	0.483000000000000
662	0.482800000000000
663	0.483600000000000
664	0.483400000000000

665	0.483400000000000
666	0.484400000000000
667	0.484100000000000
668	0.484600000000000
669	0.484800000000000
670	0.484800000000000
671	0.485200000000000
672	0.485200000000000
673	0.485800000000000
674	0.486200000000000
675	0.486500000000000
676	0.487000000000000
677	0.486700000000000
678	0.487100000000000
679	0.487200000000000
680	0.487300000000000
681	0.487700000000000
682	0.488000000000000
683	0.487800000000000
684	0.488200000000000
685	0.488300000000000
686	0.488800000000000
687	0.488600000000000
688	0.488900000000000
689	0.488700000000000
690	0.489300000000000
691	0.489800000000000
692	0.490100000000000
693	0.490000000000000
694	0.490800000000000
695	0.490500000000000
696	0.490600000000000
697	0.491100000000000
698	0.490800000000000

699	0.491200000000000
700	0.491200000000000
701	0.491700000000000
702	0.491700000000000
703	0.492000000000000
704	0.492400000000000
705	0.492600000000000
706	0.492800000000000
707	0.492600000000000
708	0.493200000000000
709	0.493700000000000
710	0.493800000000000
711	0.493700000000000
712	0.494400000000000
713	0.494600000000000
714	0.495200000000000
715	0.494600000000000
716	0.495000000000000
717	0.495100000000000
718	0.495300000000000
719	0.495300000000000
720	0.495900000000000
721	0.496000000000000
722	0.495700000000000
723	0.496100000000000
724	0.496400000000000
725	0.496600000000000
726	0.497100000000000
727	0.497300000000000
728	0.497300000000000
729	0.497800000000000
730	0.498300000000000
731	0.498300000000000
732	0.498600000000000

733	0.498800000000000
734	0.499300000000000
735	0.499400000000000
736	0.499400000000000
737	0.499400000000000
738	0.499600000000000
739	0.499600000000000
740	0.499700000000000
741	0.500500000000000
742	0.500400000000000
743	0.500600000000000
744	0.500700000000000
745	0.500900000000000
746	0.501400000000000
747	0.501600000000000
748	0.501900000000000
749	0.502300000000000
750	0.502600000000000
751	0.502700000000000
752	0.502900000000000
753	0.503100000000000
754	0.503400000000000
755	0.503600000000000
756	0.503300000000000
757	0.503700000000000
758	0.503600000000000
759	0.504400000000000
760	0.504100000000000
761	0.504300000000000
762	0.504700000000000
763	0.505000000000000
764	0.505200000000000
765	0.505800000000000
766	0.505500000000000

767	0.506000000000000
768	0.506300000000000
769	0.506900000000000
770	0.506800000000000
771	0.506500000000000
772	0.507100000000000
773	0.506900000000000
774	0.507100000000000
775	0.507200000000000
776	0.507200000000000
777	0.507800000000000
778	0.507900000000000
779	0.507900000000000
780	0.508100000000000
781	0.508500000000000
782	0.508700000000000
783	0.508700000000000
784	0.509300000000000
785	0.509200000000000
786	0.509700000000000
787	0.509900000000000
788	0.510000000000000
789	0.510300000000000
790	0.510400000000000
791	0.511300000000000
792	0.511100000000000
793	0.511200000000000
794	0.511600000000000
795	0.511300000000000
796	0.511400000000000
797	0.512300000000000
798	0.512400000000000
799	0.512300000000000
800	0.512300000000000

801	0.512900000000000
802	0.512900000000000
803	0.513500000000000
804	0.513400000000000
805	0.513800000000000
806	0.514100000000000
807	0.514400000000000
808	0.513600000000000
809	0.514100000000000
810	0.513800000000000
811	0.514400000000000
812	0.514700000000000
813	0.515200000000000
814	0.514700000000000
815	0.515600000000000
816	0.515600000000000
817	0.515700000000000
818	0.515500000000000
819	0.515800000000000
820	0.516200000000000
821	0.516400000000000
822	0.517000000000000
823	0.517000000000000
824	0.517200000000000
825	0.517600000000000
826	0.517800000000000
827	0.517600000000000
828	0.517700000000000
829	0.517900000000000
830	0.518000000000000
831	0.518400000000000
832	0.518400000000000
833	0.518600000000000
834	0.518700000000000

835	0.519100000000000
836	0.519100000000000
837	0.519700000000000
838	0.519500000000000
839	0.520100000000000
840	0.520200000000000
841	0.520600000000000
842	0.521100000000000
843	0.520800000000000
844	0.520800000000000
845	0.521400000000000
846	0.521300000000000
847	0.521000000000000
848	0.521900000000000
849	0.521500000000000
850	0.522000000000000
851	0.522700000000000
852	0.522700000000000
853	0.522800000000000
854	0.523100000000000
855	0.523400000000000
856	0.523700000000000
857	0.523700000000000
858	0.524000000000000
859	0.524000000000000
860	0.524400000000000
861	0.524200000000000
862	0.524800000000000
863	0.524600000000000
864	0.525100000000000
865	0.525400000000000
866	0.525400000000000
867	0.525600000000000
868	0.525400000000000

869	0.525700000000000
870	0.526100000000000
871	0.526300000000000
872	0.526300000000000
873	0.526700000000000
874	0.526900000000000
875	0.526900000000000
876	0.527200000000000
877	0.527300000000000
878	0.527700000000000
879	0.527700000000000
880	0.528200000000000
881	0.528700000000000
882	0.528300000000000
883	0.528400000000000
884	0.529300000000000
885	0.529000000000000
886	0.529600000000000
887	0.529400000000000
888	0.529600000000000
889	0.529900000000000
890	0.530400000000000
891	0.530100000000000
892	0.530100000000000
893	0.530600000000000
894	0.531400000000000
895	0.531000000000000
896	0.531000000000000
897	0.531100000000000
898	0.531300000000000
899	0.531600000000000
900	0.531900000000000
901	0.532100000000000
902	0.532300000000000

903	0.532700000000000
904	0.532500000000000
905	0.532600000000000
906	0.533200000000000
907	0.533200000000000
908	0.533200000000000
909	0.534100000000000
910	0.533800000000000
911	0.534000000000000
912	0.534200000000000
913	0.534500000000000
914	0.534200000000000
915	0.535000000000000
916	0.534600000000000
917	0.535200000000000
918	0.535000000000000
919	0.535300000000000
920	0.535600000000000
921	0.535500000000000
922	0.535700000000000
923	0.536000000000000
924	0.536800000000000
925	0.536900000000000
926	0.537100000000000
927	0.537000000000000
928	0.537700000000000
929	0.537800000000000
930	0.537500000000000
931	0.537700000000000
932	0.537800000000000
933	0.538500000000000
934	0.538200000000000
935	0.539000000000000
936	0.538700000000000

937	0.538800000000000
938	0.539000000000000
939	0.539300000000000
940	0.539200000000000
941	0.539900000000000
942	0.539600000000000
943	0.539900000000000
944	0.540400000000000
945	0.540300000000000
946	0.540700000000000
947	0.540900000000000
948	0.541200000000000
949	0.541300000000000
950	0.541500000000000
951	0.541900000000000
952	0.542000000000000
953	0.542200000000000
954	0.542400000000000
955	0.542100000000000
956	0.542400000000000
957	0.543200000000000
958	0.543000000000000
959	0.543000000000000
960	0.543200000000000
961	0.543400000000000
962	0.543900000000000
963	0.544100000000000
964	0.544300000000000
965	0.544600000000000
966	0.544900000000000
967	0.545000000000000
968	0.545200000000000
969	0.545400000000000
970	0.545500000000000

971	0.546200000000000
972	0.545900000000000
973	0.546300000000000
974	0.546200000000000
975	0.546300000000000
976	0.546500000000000
977	0.546800000000000
978	0.547000000000000
979	0.546900000000000
980	0.547100000000000
981	0.548000000000000
982	0.547900000000000
983	0.547900000000000
984	0.548500000000000
985	0.548200000000000
986	0.548700000000000
987	0.548000000000000
988	0.548300000000000
989	0.548800000000000
990	0.549000000000000
991	0.549100000000000
992	0.549300000000000
993	0.550000000000000
994	0.549800000000000
995	0.550100000000000
996	0.550200000000000
997	0.550600000000000
998	0.550700000000000
999	0.550600000000000
1000	0.550500000000000
1001	0.550900000000000
1002	0.551100000000000
1003	0.551700000000000
1004	0.551800000000000

1005	0.552000000000000
1006	0.552100000000000
1007	0.552400000000000
1008	0.552200000000000
1009	0.552400000000000
1010	0.552500000000000
1011	0.552700000000000
1012	0.553500000000000
1013	0.553500000000000
1014	0.553700000000000
1015	0.554100000000000
1016	0.553600000000000
1017	0.553900000000000
1018	0.554300000000000
1019	0.554500000000000
1020	0.554500000000000
1021	0.554600000000000
1022	0.554500000000000
1023	0.554800000000000
1024	0.555600000000000
1025	0.555800000000000
1026	0.555700000000000
1027	0.555700000000000
1028	0.555800000000000
1029	0.555900000000000
1030	0.556000000000000
1031	0.556300000000000
1032	0.556700000000000
1033	0.556900000000000
1034	0.557600000000000
1035	0.557300000000000
1036	0.557700000000000
1037	0.557600000000000
1038	0.558200000000000

1039	0.558000000000000
1040	0.558200000000000
1041	0.558400000000000
1042	0.558800000000000
1043	0.559100000000000
1044	0.558800000000000
1045	0.558800000000000
1046	0.559400000000000
1047	0.559700000000000
1048	0.560100000000000
1049	0.559800000000000
1050	0.560000000000000
1051	0.560100000000000
1052	0.560000000000000
1053	0.560400000000000
1054	0.560700000000000
1055	0.560600000000000
1056	0.560900000000000
1057	0.560800000000000
1058	0.561300000000000
1059	0.561700000000000
1060	0.562000000000000
1061	0.562100000000000
1062	0.562300000000000
1063	0.562300000000000
1064	0.562300000000000
1065	0.562800000000000
1066	0.562900000000000
1067	0.562700000000000
1068	0.563300000000000
1069	0.563400000000000
1070	0.563600000000000
1071	0.563700000000000
1072	0.564000000000000

1073	0.563800000000000
1074	0.564500000000000
1075	0.564300000000000
1076	0.564100000000000
1077	0.564700000000000
1078	0.564900000000000
1079	0.565000000000000
1080	0.565600000000000
1081	0.565600000000000
1082	0.565600000000000
1083	0.566100000000000
1084	0.566200000000000
1085	0.566000000000000
1086	0.566300000000000
1087	0.566100000000000
1088	0.566500000000000
1089	0.566800000000000
1090	0.566800000000000
1091	0.567400000000000
1092	0.567500000000000
1093	0.567800000000000
1094	0.567300000000000
1095	0.568500000000000
1096	0.568700000000000
1097	0.568600000000000
1098	0.568300000000000
1099	0.568300000000000
1100	0.568700000000000
1101	0.568800000000000
1102	0.569200000000000
1103	0.569500000000000
1104	0.569400000000000
1105	0.569500000000000
1106	0.570100000000000

1107	0.569800000000000
1108	0.570400000000000
1109	0.570000000000000
1110	0.570700000000000
1111	0.571000000000000
1112	0.571500000000000
1113	0.571300000000000
1114	0.571300000000000
1115	0.571300000000000
1116	0.571400000000000
1117	0.572200000000000
1118	0.572100000000000
1119	0.572500000000000
1120	0.572700000000000
1121	0.572300000000000
1122	0.572500000000000
1123	0.573000000000000
1124	0.573100000000000
1125	0.573200000000000
1126	0.573700000000000
1127	0.573700000000000
1128	0.574000000000000
1129	0.574500000000000
1130	0.574300000000000
1131	0.574600000000000
1132	0.574900000000000
1133	0.575200000000000
1134	0.575200000000000
1135	0.575300000000000
1136	0.575200000000000
1137	0.575200000000000
1138	0.576500000000000
1139	0.576100000000000
1140	0.576400000000000

1141	0.576400000000000
1142	0.576400000000000
1143	0.576600000000000
1144	0.576500000000000
1145	0.577100000000000
1146	0.577500000000000
1147	0.577200000000000
1148	0.577800000000000
1149	0.577700000000000
1150	0.578300000000000
1151	0.578800000000000
1152	0.578300000000000
1153	0.578600000000000
1154	0.578900000000000
1155	0.579000000000000
1156	0.578800000000000
1157	0.579200000000000
1158	0.579500000000000
1159	0.579700000000000
1160	0.579600000000000
1161	0.580000000000000
1162	0.580200000000000
1163	0.580500000000000
1164	0.580700000000000
1165	0.580700000000000
1166	0.581200000000000
1167	0.581000000000000
1168	0.581600000000000
1169	0.581900000000000
1170	0.581700000000000
1171	0.581800000000000
1172	0.582200000000000
1173	0.582200000000000
1174	0.582700000000000

1175	0.582700000000000
1176	0.583300000000000
1177	0.583500000000000
1178	0.583700000000000
1179	0.583700000000000
1180	0.584200000000000
1181	0.584200000000000
1182	0.584700000000000
1183	0.584700000000000
1184	0.585100000000000
1185	0.585100000000000
1186	0.585300000000000
1187	0.585500000000000
1188	0.586300000000000
1189	0.585700000000000
1190	0.586200000000000
1191	0.586900000000000
1192	0.586700000000000
1193	0.587300000000000
1194	0.587300000000000
1195	0.587300000000000
1196	0.587800000000000
1197	0.588000000000000
1198	0.588300000000000
1199	0.588600000000000
1200	0.588400000000000
1201	0.588700000000000
1202	0.588700000000000
1203	0.589300000000000
1204	0.589300000000000
1205	0.589700000000000
1206	0.590100000000000
1207	0.590800000000000
1208	0.590300000000000

1209	0.591200000000000
1210	0.591700000000000
1211	0.591200000000000
1212	0.591300000000000
1213	0.592000000000000
1214	0.591600000000000
1215	0.592300000000000
1216	0.592300000000000
1217	0.593000000000000
1218	0.592800000000000
1219	0.593600000000000
1220	0.593300000000000
1221	0.593400000000000
1222	0.594000000000000
1223	0.594000000000000
1224	0.594100000000000
1225	0.594500000000000
1226	0.594900000000000
1227	0.595200000000000
1228	0.595300000000000
1229	0.595800000000000
1230	0.596000000000000
1231	0.596000000000000
1232	0.596500000000000
1233	0.596500000000000
1234	0.596100000000000
1235	0.597100000000000
1236	0.597200000000000

1-Cu^{II} 2.0 mM, K_2CO_3 10 mM, HPNP 0.20 mM, TCA 10 mM, no pH adjustment.
See Fig. 5 in the main text.

t(min) A_{400nm} (a.u.)

0	0.126400000000000
1	0.116900000000000
2	0.121600000000000
3	0.125000000000000
4	0.129600000000000
5	0.133200000000000
6	0.137100000000000
7	0.141000000000000
8	0.144600000000000
9	0.148100000000000
10	0.151800000000000
11	0.155200000000000
12	0.158400000000000
13	0.161900000000000
14	0.165300000000000
15	0.169200000000000
16	0.172200000000000
17	0.176100000000000
18	0.180100000000000
19	0.182700000000000
20	0.186400000000000
21	0.189900000000000
22	0.193000000000000
23	0.196100000000000
24	0.199900000000000
25	0.203200000000000
26	0.206700000000000
27	0.209600000000000
28	0.212600000000000
29	0.215800000000000
30	0.218800000000000
31	0.221900000000000

1-Cu^{II} 2.0 mM, K_2CO_3 10 mM, HPNP 0.20 mM, TCA 15 mM, no pH adjustment.
See Fig. 5 in the main text.

t(min)	A_{400nm} (a.u.)
0	0.116500000000000
1	0.103600000000000
2	0.103200000000000
3	0.103400000000000
4	0.102700000000000
5	0.102300000000000
6	0.102800000000000
7	0.102200000000000
8	0.102600000000000
9	0.102600000000000
10	0.102400000000000
11	0.101900000000000
12	0.101300000000000
13	0.102000000000000
14	0.102300000000000
15	0.102400000000000
16	0.102300000000000
17	0.102200000000000
18	0.102500000000000
19	0.102100000000000
20	0.102800000000000
21	0.102200000000000
22	0.101900000000000
23	0.102800000000000
24	0.104700000000000
25	0.110400000000000
26	0.114800000000000
27	0.119300000000000
28	0.123200000000000
29	0.127000000000000

30	0.131100000000000
31	0.134400000000000
32	0.138100000000000
33	0.141900000000000
34	0.145700000000000
35	0.148500000000000
36	0.151800000000000
37	0.155600000000000
38	0.158600000000000
39	0.161900000000000
40	0.165600000000000
41	0.168400000000000
42	0.171400000000000
43	0.175000000000000
44	0.178100000000000
45	0.181100000000000
46	0.183600000000000
47	0.186100000000000
48	0.189100000000000
49	0.191800000000000
50	0.194200000000000
51	0.196700000000000
52	0.199600000000000
53	0.202100000000000
54	0.205000000000000
55	0.207500000000000

1-Cu^{II} 2.0 mM, K_2CO_3 10 mM, HPNP 0.20 mM, TCA 20 mM, no pH adjustment.
See Fig. 5 in the main text.

t(min)	A_{400nm} (a.u.)
0	0.125400000000000

1	0.103800000000000
2	0.102600000000000
3	0.102800000000000
4	0.102200000000000
5	0.102000000000000
6	0.102000000000000
7	0.102400000000000
8	0.101900000000000
9	0.102100000000000
10	0.101800000000000
11	0.101800000000000
12	0.102100000000000
13	0.101800000000000
14	0.101700000000000
15	0.102200000000000
16	0.101900000000000
17	0.102100000000000
18	0.101900000000000
19	0.101900000000000
20	0.101900000000000
21	0.101500000000000
22	0.101300000000000
23	0.102000000000000
24	0.101800000000000
25	0.101400000000000
26	0.101700000000000
27	0.101900000000000
28	0.101900000000000
29	0.101500000000000
30	0.101600000000000
31	0.101900000000000
32	0.101500000000000
33	0.101600000000000
34	0.101500000000000

35	0.101300000000000
36	0.101200000000000
37	0.101000000000000
38	0.101700000000000
39	0.101400000000000
40	0.101900000000000
41	0.103800000000000
42	0.108300000000000
43	0.112900000000000
44	0.117400000000000
45	0.120400000000000
46	0.124500000000000
47	0.128000000000000
48	0.131100000000000
49	0.134100000000000
50	0.137600000000000
51	0.141600000000000
52	0.145000000000000
53	0.148500000000000
54	0.151100000000000
55	0.153700000000000
56	0.156200000000000
57	0.159300000000000
58	0.162300000000000
59	0.165400000000000
60	0.167800000000000
61	0.171200000000000
62	0.173900000000000
63	0.176500000000000
64	0.179400000000000
65	0.181800000000000
66	0.184400000000000
67	0.187100000000000
68	0.189500000000000

69	0.191700000000000
70	0.194000000000000
71	0.196900000000000

2-Cu^{II} xxxxxx mM, K_2CO_3 10 mM, HPNP 50 μM, TCA 10 mM, no prior pH adjustment. See Fig. 6 in the main text.

t(min)	A_{400nm} (a.u.)
0	0.165000000000000
1	0.163000000000000
2	0.167000000000000
3	0.169000000000000
4	0.175000000000000
5	0.180000000000000
6	0.192000000000000
7	0.205000000000000
8	0.228000000000000
9	0.242000000000000
10	0.254000000000000
11	0.269000000000000
12	0.289000000000000
13	0.309000000000000
14	0.324000000000000
15	0.342000000000000
16	0.353000000000000
17	0.374000000000000
18	0.384000000000000
19	0.396000000000000
20	0.408000000000000
21	0.413000000000000
22	0.415000000000000
23	0.153000000000000
24	0.153000000000000

25	0.153000000000000
26	0.153000000000000
27	0.153000000000000
28	0.153000000000000
29	0.155000000000000
30	0.158000000000000
31	0.159000000000000
32	0.159000000000000
33	0.161000000000000
34	0.160000000000000
35	0.161000000000000
36	0.162000000000000
37	0.162000000000000
38	0.163000000000000
39	0.163000000000000
40	0.164000000000000
41	0.164000000000000
42	0.165000000000000
43	0.162000000000000
44	0.163000000000000
45	0.162000000000000
46	0.168000000000000
47	0.170000000000000
48	0.172000000000000
49	0.176000000000000
50	0.178000000000000
51	0.183000000000000
52	0.186000000000000
53	0.189000000000000
54	0.198000000000000
55	0.215000000000000
56	0.229000000000000
57	0.236000000000000
58	0.249000000000000

59	0.258000000000000
60	0.287000000000000
61	0.331000000000000
62	0.372000000000000
63	0.389000000000000
64	0.412000000000000
65	0.423000000000000
66	0.429000000000000
67	0.438000000000000
68	0.446000000000000
69	0.459000000000000
70	0.465000000000000
71	0.474000000000000
72	0.480000000000000
73	0.486000000000000
74	0.492000000000000
75	0.501000000000000
76	0.506000000000000
77	0.512000000000000
78	0.515000000000000
79	0.517000000000000
80	0.519000000000000
81	0.523000000000000
82	0.527000000000000
83	0.531000000000000
84	0.532000000000000
85	0.537000000000000
86	0.541000000000000
87	0.544000000000000
88	0.159000000000000
89	0.161000000000000
90	0.161000000000000
91	0.162000000000000
92	0.161000000000000

93	0.161000000000000
94	0.160000000000000
95	0.162000000000000
96	0.162000000000000
97	0.161000000000000
98	0.164000000000000
99	0.163000000000000
100	0.163000000000000
101	0.162000000000000
102	0.162000000000000
103	0.164000000000000
104	0.165000000000000
105	0.165000000000000
106	0.165000000000000
107	0.166000000000000
108	0.166000000000000
109	0.167000000000000
110	0.168000000000000
111	0.170000000000000
112	0.171000000000000
113	0.172000000000000
114	0.173000000000000
115	0.174000000000000
116	0.175000000000000
117	0.176000000000000
118	0.177000000000000
119	0.178000000000000
120	0.178000000000000
121	0.179000000000000
122	0.180000000000000
123	0.182000000000000
124	0.184000000000000
125	0.187000000000000
126	0.191000000000000

127	0.196000000000000
128	0.204000000000000
129	0.212000000000000
130	0.221000000000000
131	0.231000000000000
132	0.242000000000000
133	0.254000000000000
134	0.267000000000000
135	0.281000000000000
136	0.296000000000000
137	0.312000000000000
138	0.330000000000000
139	0.350000000000000
140	0.373000000000000
141	0.398000000000000
142	0.426000000000000
143	0.456000000000000
144	0.488000000000000
145	0.521000000000000
146	0.552000000000000
147	0.584000000000000
148	0.615000000000000
149	0.644000000000000
150	0.673000000000000
151	0.698000000000000
152	0.718000000000000
153	0.736000000000000
154	0.746000000000000
155	0.754000000000000
156	0.761000000000000
157	0.767000000000000
158	0.773000000000000
159	0.778000000000000
160	0.781000000000000

161	0.784000000000000
162	0.787000000000000
163	0.790000000000000
164	0.793000000000000
165	0.795000000000000
166	0.797000000000000
167	0.799000000000000
168	0.801000000000000
169	0.803000000000000
170	0.805000000000000
171	0.807000000000000
172	0.809000000000000
173	0.811000000000000
174	0.812000000000000
175	0.813000000000000

2-Cu^{II} xxxxx mM, K_2CO_3 10 mM, HPNP 50 μM, TCA 20 mM, no prior pH adjustment. See Fig. 6 in the main text.

t(min)	A_{400nm} (a.u.)
0	0.163000000000000
1	0.149000000000000
2	0.148000000000000
3	0.148000000000000
4	0.148000000000000
5	0.148000000000000
6	0.149000000000000
7	0.148000000000000
8	0.148000000000000
9	0.144000000000000
10	0.147000000000000
11	0.149000000000000

12	0.148000000000000
13	0.148000000000000
14	0.148000000000000
15	0.148000000000000
16	0.148000000000000
17	0.148000000000000
18	0.148000000000000
19	0.148000000000000
20	0.148000000000000
21	0.148000000000000
22	0.148000000000000
23	0.148000000000000
24	0.149000000000000
25	0.145000000000000
26	0.148000000000000
27	0.151000000000000
28	0.148000000000000
29	0.148000000000000
30	0.148000000000000
31	0.148000000000000
32	0.154000000000000
33	0.159000000000000
34	0.163000000000000
35	0.165000000000000
36	0.169000000000000
37	0.181000000000000
38	0.186000000000000
39	0.192000000000000
40	0.201000000000000
41	0.212000000000000
42	0.223000000000000
43	0.232000000000000
44	0.244000000000000
45	0.251000000000000

46	0.263000000000000
47	0.274000000000000
48	0.285000000000000
49	0.297000000000000
50	0.309000000000000
51	0.322000000000000
52	0.336000000000000
53	0.349000000000000
54	0.362000000000000
55	0.375000000000000
56	0.387000000000000
57	0.397000000000000
58	0.406000000000000
59	0.415000000000000
60	0.425000000000000
61	0.434000000000000
62	0.443000000000000
63	0.452000000000000
64	0.461000000000000
65	0.470000000000000
66	0.479000000000000
67	0.488000000000000
68	0.497000000000000
69	0.507000000000000
70	0.518000000000000
71	0.525000000000000
72	0.533000000000000
73	0.541000000000000
74	0.174000000000000
75	0.174000000000000
76	0.174000000000000
77	0.175000000000000
78	0.174000000000000
79	0.176000000000000

80	0.174000000000000
81	0.175000000000000
82	0.173000000000000
83	0.174000000000000
84	0.175000000000000
85	0.175000000000000
86	0.176000000000000
87	0.177000000000000
88	0.177000000000000
89	0.178000000000000
90	0.177000000000000
91	0.178000000000000
92	0.176000000000000
93	0.179000000000000
94	0.181000000000000
95	0.182000000000000
96	0.183000000000000
97	0.182000000000000
98	0.183000000000000
99	0.184000000000000
100	0.185000000000000
101	0.186000000000000
102	0.185000000000000
103	0.184000000000000
104	0.185000000000000
105	0.186000000000000
106	0.186000000000000
107	0.187000000000000
108	0.186000000000000
109	0.188000000000000
110	0.187000000000000
111	0.189000000000000
112	0.191000000000000
113	0.190000000000000

114	0.191000000000000
115	0.193000000000000
116	0.192000000000000
117	0.193000000000000
118	0.194000000000000
119	0.193000000000000
120	0.193000000000000
121	0.194000000000000
122	0.195000000000000
123	0.196000000000000
124	0.197000000000000
125	0.198000000000000
126	0.199000000000000
127	0.201000000000000
128	0.200000000000000
129	0.201000000000000
130	0.202000000000000
131	0.203000000000000
132	0.205000000000000
133	0.206000000000000
134	0.208000000000000
135	0.210000000000000
136	0.212000000000000
137	0.215000000000000
138	0.218000000000000
139	0.221000000000000
140	0.224000000000000
141	0.227000000000000
142	0.230000000000000
143	0.234000000000000
144	0.238000000000000
145	0.241000000000000
146	0.244000000000000
147	0.247000000000000

148	0.250000000000000
149	0.254000000000000
150	0.259000000000000
151	0.265000000000000
152	0.271000000000000
153	0.276000000000000
154	0.283000000000000
155	0.291000000000000
156	0.298000000000000
157	0.307000000000000
158	0.317000000000000
159	0.327000000000000
160	0.338000000000000
161	0.349000000000000
162	0.360000000000000
163	0.372000000000000
164	0.384000000000000
165	0.396000000000000
166	0.409000000000000
167	0.423000000000000
168	0.438000000000000
169	0.454000000000000
170	0.471000000000000
171	0.488000000000000
172	0.506000000000000
173	0.524000000000000
174	0.542000000000000
175	0.559000000000000
176	0.575000000000000
177	0.590000000000000
178	0.605000000000000
179	0.619000000000000
180	0.632000000000000
181	0.644000000000000

182	0.656000000000000
183	0.668000000000000
184	0.680000000000000
185	0.692000000000000
186	0.703000000000000
187	0.713000000000000
188	0.722000000000000
189	0.731000000000000
190	0.744000000000000
191	0.751000000000000
192	0.757000000000000
193	0.763000000000000
194	0.768000000000000
195	0.773000000000000
196	0.777000000000000
197	0.781000000000000
198	0.785000000000000
199	0.789000000000000
200	0.792000000000000
201	0.794000000000000
202	0.796000000000000
203	0.798000000000000
204	0.800000000000000
205	0.802000000000000
206	0.804000000000000
207	0.805000000000000
208	0.806000000000000
209	0.807000000000000
210	0.808000000000000
211	0.809000000000000
212	0.810000000000000
213	0.812000000000000
214	0.814000000000000
215	0.816000000000000

216	0.817000000000000
217	0.818000000000000
218	0.819000000000000
219	0.820000000000000
220	0.821000000000000
221	0.822000000000000

S6 Potentiometric data from previous investigations

The following data are taken from our previous investigation *J. Org. Chem* 2015, 80, 5887 and *J. Org. Chem* 2011, 76, 5438 (cited as references 43 and 65 in the main text). A reviewer asked details about the species present in solution. Therefore, we report these data for the reader convenience.

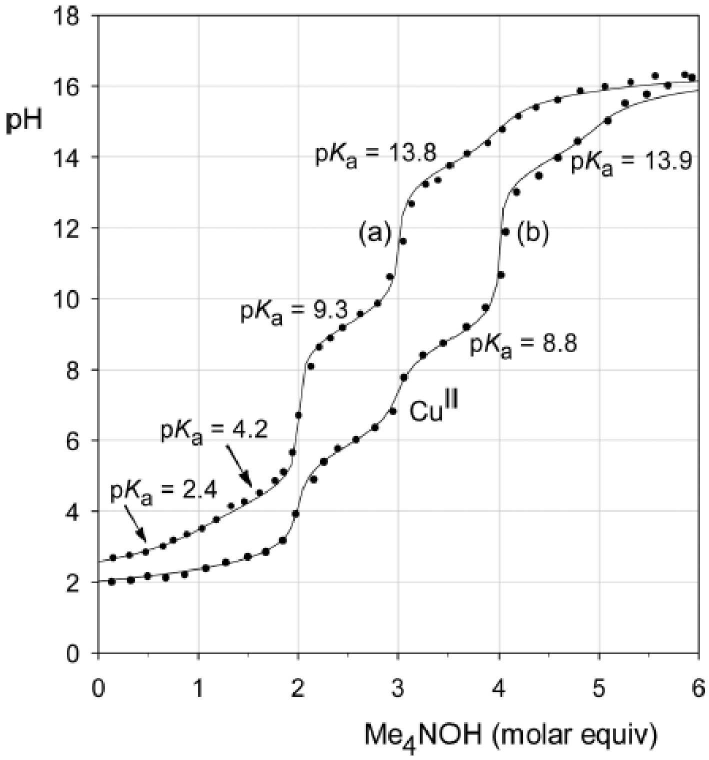

Figure S6.1 Potentiometric titration of 1.0 mM **2** with Me_4NOH in 80% DMSO in the absence (a) and presence (b) of 1 molar equiv of Cu^{II}. Data points are experimental and the lines are calculated. The Log K_{Cu} = 9.2

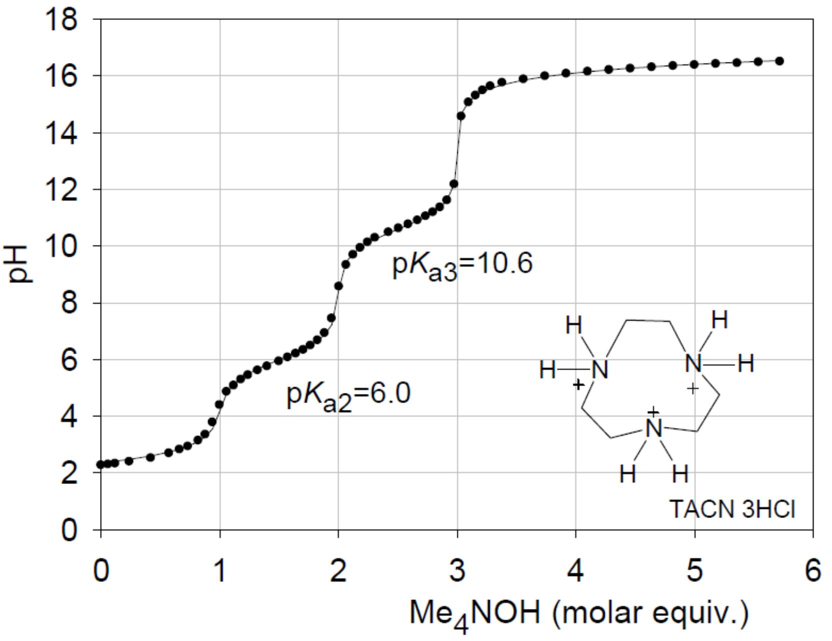

Figure S6.2 Potentiometric titration of 1.0 mM TACN with Me_4NOH in 80% DMSO

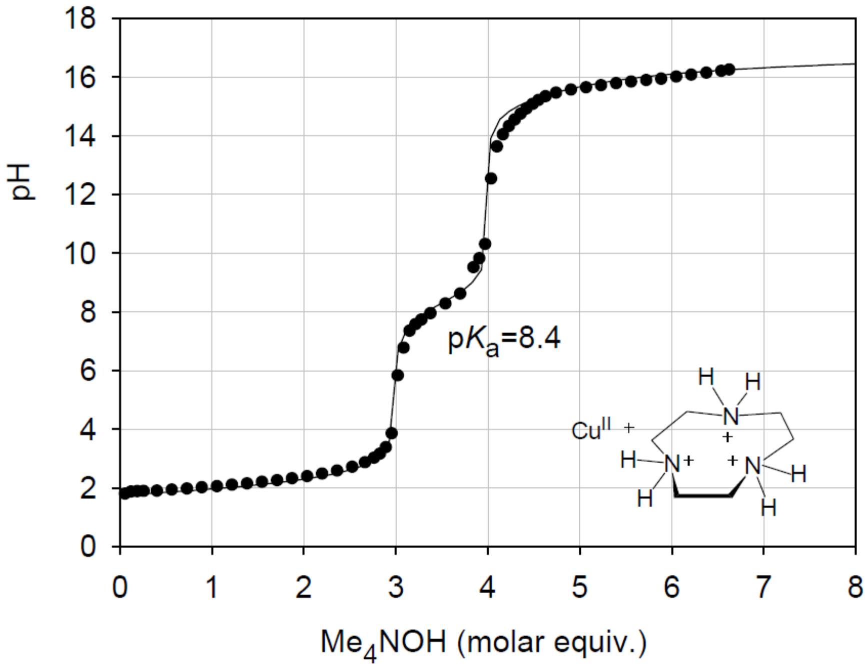

Figure S6.3 Potentiometric titration of 1.0 mM TACN with Me_4NOH in 80% DMSO in the presence of equimolar amount of Cu^{II}.Log K_{Cu} = 11.3
